# Migraine care in the digital era: social listening insights and neurologists’ perspectives from Türkiye

**DOI:** 10.3389/fdgth.2026.1891640

**Published:** 2026-09-14

**Authors:** Derya Uluduz, Pınar Yalınay Dikmen, Burcin Yazici, Aynur Ozge

**Affiliations:** 1 School of Medicine, Department of Neurology, Istanbul Atlas University, Istanbul, Türkiye; 2 School of Medicine, Department of Neurology, Acıbadem University, Istanbul, Türkiye; 3 Internal Medicine, Pfizer Biopharmaceuticals, Istanbul, Türkiye; 4 Neurology Clinic, Brain Health Clinic, Mersin, Türkiye; 5 Neurology Clinic, NOROM Neuroscience and Excellence Center, Ankara, Türkiye

**Keywords:** digital era, digital health literacy, migraine, patient journey, physician perspectives, social listening, social media, Türkiye

## Abstract

**Objective:**

To explore migraine-related concerns expressed in user-generated digital entries from a Turkish online health platform and contextualize them through neurologists’ expert perspectives. Background: Digital platforms may shape symptom interpretation, treatment expectations, and communication; however, social-listening findings require cautious interpretation given platform-related and methodological limitations.

**Methods:**

This exploratory study combined secondary interpretation of a pre-existing proprietary Kimola social-listening report with a survey of 10 neurologists with headache expertise. The report included analytical outputs from 2,500 migraine-related user-generated entries posted on Doktorsitesi.com between 2007 and 2023. Because raw entries, preprocessing records, and coding files were unavailable to the authors, findings were interpreted descriptively as source-report outputs. Neurologist responses were summarized as raw counts and narratively contextualized with entry-derived themes, without formal mixed-methods integration.

**Results:**

According to the source report, entries were categorized into four migraine-journey stages: pre-treatment, treatment phase, disease experience, and post-treatment. These stages reflected symptom interpretation and diagnostic uncertainty, treatment effectiveness and safety concerns, broader disease burden, and discontinuation or interest in non-oral, procedural, or complementary options after treatment. In the survey, 7 of 10 neurologists agreed or strongly agreed that many people with migraine use social media for migraine-related information, and 9 of 10 agreed or strongly agreed that misinformation complicates physician–patient communication.

**Conclusions:**

Among digitally engaged platform users, migraine-related entries revealed unmet information needs and treatment-related concerns. Neurologists highlighted the need for expert-led, evidence-based digital education. Although exploratory and not statistically generalizable, these findings may inform communication and digital education strategies in migraine care.

## 1 Introduction

Migraine is a highly prevalent and disabling neurological disorder, affecting more than one billion individuals worldwide and ranking among the leading causes of years lived with disability, particularly in young adults and women under the age of 50 (1–3). Despite this substantial burden, migraine remains underdiagnosed and undertreated, reflecting persistent gaps in timely diagnosis, access to appropriate care, optimal treatment, and patient education (1, 4, 5). These gaps contribute not only to impaired quality of life but also to a considerable socioeconomic burden for patients, healthcare systems, and society (1, 4, 5). The impact of migraine may be further amplified in country-specific healthcare settings, where differences in awareness, healthcare access, referral pathways, and resource allocation influence the patient journey (5, 6). In Türkiye, the prevalence of migraine is approximately 16%, broadly comparable with global estimates, and the condition disproportionately affects women and individuals aged 25–45 years, underscoring its relevance as both a public health and health services issue (5, 6).

In parallel, the way patients seek and interpret health information has changed substantially. Digital platforms have become common sources of symptom interpretation, treatment information, and peer support, often preceding or supplementing contact with healthcare professionals (7–9). This shift is particularly relevant in migraine, a disorder characterized by recurrent, subjective, and heterogeneous symptoms that may lead individuals to search repeatedly for explanations, reassurance, and treatment options outside the clinical encounter (7–9). Digital environments can support patient engagement by improving access to experiential knowledge and practical information; however, their value is constrained by variable digital health literacy, inconsistent quality of online content, and widespread exposure to misinformation (10–12). As a result, online information may shape expectations and complicate physician–patient communication when it is not aligned with evidence-based care (11, 12). In Türkiye, these challenges may be reinforced by limited migraine awareness at the first point of care, delayed specialist referral, and continuing gaps in care utilization (6, 13, 14).

The growing role of digital media in health-information seeking highlights the need to examine migraine care not only through clinical data, but also through user-generated digital entries. Social listening offers an opportunity to capture real-world concerns, misconceptions, information needs, and treatment expectations that may not be fully expressed during clinical consultations. However, user-generated online data require clinical contextualization, particularly when they are used to understand care-related concerns or inform patient-centered strategies. Contextualizing themes derived from digital entries through neurologists’ expert-informed perspectives may therefore help clarify how digitally shaped concerns intersect with diagnosis, treatment decisions, expectation management, and continuity of care.

The present study aimed to explore how migraine-related concerns, uncertainties, and treatment expectations are expressed in user-generated digital entries from a Turkish online health platform, and to contextualize these findings through neurologists’ expert-informed perspectives. The objective was not to provide an epidemiological estimate of migraine-related online behavior or a representative assessment of migraine care in Türkiye. Rather, the study focused on the digital migraine care journey among online platform users and on how these digitally expressed concerns may intersect with clinical communication, treatment expectation management, and patient education. By narratively juxtaposing social listening source-report outputs with neurologists’ perspectives, the study sought to identify clinically relevant areas in which digitally expressed migraine-related concerns may inform clinical communication, expectation management, and patient education.

## 2 Methods

### 2.1 Study design

This study was conducted as an exploratory secondary analysis combining qualitative interpretation of a pre-existing social listening report with descriptive survey data from neurologists in Türkiye. The aim was to examine digitally expressed migraine-related concerns among users of a Turkish online health platform and to contextualize these themes through neurologists’ expert-informed perspectives. As the social listening component was based on pre-processed secondary data without direct access to the raw dataset, the findings should be interpreted as exploratory and hypothesis-generating. The study was designed to examine digitally expressed migraine-related concerns rather than to estimate the prevalence of migraine behaviors or treatment patterns in the general population. The physician survey was used as an expert-informed contextualization component to interpret the clinical relevance of the user-generated digital themes.

### 2.2 Social listening data

Within the scope of this study, the social listening component represents the structured analysis of publicly accessible, migraine-related user-generated entries from a single online health platform, as reported in a pre-existing proprietary Kimola source report. It was not based on real-time monitoring or independent primary collection of multi-platform social media data by the study authors. The source-report process involved the systematic identification, filtering, classification, and interpretation of publicly available online user-generated content to identify recurring themes, concerns, expectations, and information needs expressed by platform users. Unlike conventional surveys, this source material consisted of unsolicited user-generated entries posted in naturalistic online settings. In the present study, this content included entries posted by individuals writing about their own migraine-related concerns or, where explicitly stated, those of a relative or another person. Social listening findings are inherently shaped by the characteristics of the selected platform and its users, the search strategy and time frame, source-report data-cleaning decisions, coding procedures, and the analytical framework used to organize the content. Accordingly, these findings should be interpreted as exploratory, context-dependent patterns in migraine-related online entries rather than as representative estimates of migraine prevalence, disease burden, treatment behavior, or preferences among the broader population of people with migraine in Türkiye.

#### 2.2.1 Data source and dataset development

User-generated digital data were derived from a pre-existing proprietary social listening report prepared in 2023 by Kimola Inc, a multilingual customer feedback analytics and social listening platform that supports the organization of large volumes of user-generated feedback into structured analytical outputs. For this project, Kimola prepared a proprietary report based on publicly available user-generated content from Doktorsitesi.com, a widely used Turkish online health platform where users can submit health-related questions and receive responses from physicians (15). According to the source-report inclusion criteria, only content that was publicly accessible without user authentication or restricted access was included. The source search covered content posted between 2007 and 2023 and identified user-generated questions and comments containing the Turkish keyword “migren” (“migraine”). The analysis was restricted to user-generated content; physician responses were not included in the analytical dataset. No individuals were prospectively recruited, contacted, or selected by the study authors.

The initial dataset included approximately 2,800 migraine-related entries. During preprocessing, duplicate entries and entries that were too short, incomplete, unclear, or insufficiently informative for analysis were excluded. Following this cleaning process, 2,500 entries were retained and constituted the final source-report dataset. The unit of analysis was the individual online entry or post, rather than the individual platform user. Each entry was treated as an independent analytical unit. Personal identifiers and user-level linkage data were unavailable; therefore, multiple entries potentially originating from the same platform user could not be identified or matched and were analyzed separately. Consequently, the dataset represents 2,500 migraine-related entries and should not be interpreted as representing 2,500 unique individuals.

The dataset comprised migraine-related questions, concerns, and experiences posted by digitally engaged platform users, including individuals writing about their own or another person's migraine-related concerns. Migraine diagnoses were not independently verified. Accordingly, the findings should be interpreted as reflecting content posted by users of this specific platform rather than as representative of the broader population of people with migraine in Türkiye. The original Turkish source report and its English summary are provided as Supplementary 1 and 2, respectively.

#### 2.2.2 Data classification and analytical workflow

The user-generated component of the present study was based on secondary interpretation of the aggregated analytical outputs reported by Kimola rather than direct analysis of the raw Doktorsitesi.com dataset. The study authors did not participate in the original retrieval, cleaning, or coding of the online entries and did not have access to the raw entry-level dataset. Therefore, the social listening component of this study should be interpreted as a secondary analysis of preprocessed source-report outputs. Accordingly, the overall analytical process comprised two successive levels: development of the source-report coding outputs by Kimola and clinically informed secondary interpretation of the aggregated outputs by the study authors.

At the time the Kimola source report was prepared in 2023, the source-report analytical workflow combined conventional machine-learning–assisted screening with analyst-led qualitative coding. This machine-learning–assisted step was limited to preliminary author-role screening; it was not used to generate the thematic categories or replace manual content coding. After completion of the two preceding dataset-development steps—content identification and preprocessing/eligibility assessment, as described above—the subsequent Kimola analytical workflow comprised the following five sequential stages:

1. *Machine-learning–assisted author-role screening and manual verification***.** Supervised machine-learning models were used specifically to support the initial distinction between entries concerning the author's own migraine-related concerns and those concerning a relative or another person. All preliminary author-role assignments were subsequently reviewed manually by the Kimola research team and corrected where necessary. Entries in which authors clearly referred to their own migraine-related concerns were coded as self-related entries, whereas entries explicitly concerning a relative or another person were coded as other-person-related entries. The latter were further categorized according to the explicitly stated relationship, including parent, child, spouse, sibling, or friend. No inferential determination of user identity, relationship, diagnosis, or clinical status was made beyond the information explicitly provided in the entry.
2. *Manual content review and inductive framework development.* The retained entries were manually reviewed by the Kimola research team using qualitative content analysis and inductive thematic coding. No fixed, fully prespecified category structure was applied at the outset. Instead, recurring themes, information needs, question types, and experience domains were identified from the dataset. The emerging patterns were organized into a coherent analytical framework. The research objectives guided the structuring and naming of the resulting themes but did not determine the themes themselves.
3. *Hierarchical category assignment.* Based on the primary thematic focus of each entry, every entry was assigned to one main category and one corresponding subcategory. The four main categories generated through this process were pre-treatment phase, treatment phase, disease experience, and post-treatment phase. Assignment at both hierarchical levels was mutually exclusive; each entry was assigned to only one main category and one corresponding subcategory.
4. *Coding of additional variables and content tags.* In addition to its main category and subcategory assignment, an entry could receive supplementary codes for clinically or analytically relevant features, including specific medications, treatment modalities, health products, symptoms, adverse effects, or other descriptive themes mentioned in the text. Unlike the mutually exclusive main-category assignment, these additional tags were not necessarily mutually exclusive. More than one supplementary tag could be assigned to the same entry when relevant, and the same medication, treatment, or descriptive tag could occur across entries assigned to different main categories.
5. *Aggregation and reporting.* Main-category percentages were reported with reference to the final dataset of 2,500 entries as the denominator. Subcategory percentages were reported with reference to the number of entries assigned to the corresponding parent category as the denominator. Percentages for lower-level themes, treatment references, or descriptive tags were likewise reported within their relevant parent category or subcategory, unless otherwise specified in the source report. Because supplementary tags could co-occur within an entry, tag-level distributions should be interpreted as descriptive coded patterns and may not represent mutually exclusive proportions.

No formal assessment of inter-coder reliability was performed. The study authors did not independently reproduce, recode, or validate the original entry-level classifications. Percentages derived from the Kimola source report were retained as descriptive source-report outputs and should not be interpreted as independently verified prevalence estimates, proportions of unique individuals, or representative estimates of the broader population of people with migraine.

At the second analytical level, the study authors reviewed and reinterpreted the aggregated source-report categories and coded patterns through a clinically informed lens, focusing on their relevance to migraine literacy, diagnostic uncertainty, treatment expectations, treatment safety concerns, treatment continuation, and physician–patient communication. This process did not involve access to or reclassification of individual entries and did not modify the original Kimola coding framework.

### 2.3 Physician survey

#### 2.3.1 Participant selection

A structured expert survey was conducted among 10 neurologists practicing in Türkiye. Participants were selected through purposive sampling, whereby neurologists with specific expertise and clinical experience in headache and migraine care were invited to participate. This approach was considered appropriate for the exploratory objective of the study, as the aim was to obtain clinically informed perspectives from neurologists with substantial experience in migraine care rather than to generate statistically representative estimates. To reflect diversity as much as possible within this small expert sample, neurologists from different geographical regions of Türkiye and different healthcare settings, including university hospitals, training and research hospitals, and city hospitals, were included. Priority was given to centers with high headache and migraine patient volume and recognized clinical experience in this field. Participating centers serve as tertiary or regional referral hospitals and receive patients from surrounding provinces and less-resourced areas, which may broaden the clinical spectrum encountered by the participating neurologists. However, rural-specific neurologist representation was not systematically targeted. Eligible participants were neurologists actively involved in headache outpatient practice and had scientific experience in headache disorders, including at least one publication related to headache or migraine. The physician survey was therefore used as an expert-informed contextualization component and should not be interpreted as representative of all neurologists in Türkiye or as sufficient to support definite national conclusions.

#### 2.3.2 Survey design

The physician survey was structured around themes considered clinically relevant for contextualizing the major entry-derived domains identified in the Kimola social listening report. These included platform users’ reliance on online information, perceived misinformation, treatment expectations, medication safety and adverse-effect concerns, treatment dissatisfaction, interest in non-pharmacological or alternative approaches, and the perceived influence of social media on treatment expectations, symptom management, and physician–patient communication. The survey also assessed neurologists’ views on the perceived benefits and risks of social media in migraine care and the need for expert-led digital education. The final questionnaire included Likert-scale items, multiple-choice questions, and open-ended questions. The full questionnaire and aggregated responses are provided in Supplementary 3.

#### 2.3.3 Data analysis

Survey responses were analyzed descriptively. Because of the small expert sample, physician survey findings were reported as raw counts rather than percentages and were interpreted as expert-informed clinical perspectives rather than representative quantitative estimates. Likert-scale items were summarized by response counts, and multiple-choice responses were reported as selection frequencies. Open-ended responses were reviewed qualitatively and used to contextualize the descriptive findings.

### 2.4 Narrative synthesis of user-generated and physician-derived findings

The two data sources were not statistically integrated, and no formal mixed-methods integration framework was applied. Instead, user-generated themes derived from the Kimola source report were narratively juxtaposed with neurologists’ survey responses to explore how digitally expressed concerns could be clinically contextualized by expert perspectives.

### 2.5 Ethical considerations

The social listening component was based on aggregated analytical outputs from a pre-existing Kimola source report derived from publicly accessible user-generated content and involved no direct patient interaction. No identifiable personal data were accessed or processed by the authors. The physician survey was voluntary and anonymous, and responses were analyzed in a de-identified manner. Under local regulatory practice, this anonymous, non-interventional professional opinion survey and secondary analysis of aggregated publicly available user-generated content were considered outside the scope of interventional or patient-based clinical research requiring formal clinical trials ethics committee approval. Therefore, separate ethics committee approval or a formal exemption determination was not obtained. Any illustrative text was used in a non-identifying manner to minimize reidentification risk.

## 3 Results

### 3.1 Social listening findings

According to the Kimola source report, the final source-report dataset included 2,500 migraine-related user-generated entries. Entries were categorized into four main source-report stages of the digital migraine journey: pre-treatment phase, reported as 56% (*n* = 1,379); treatment phase, 27% (*n* = 648); disease experience, 13% (*n* = 347); and post-treatment phase, 4% (*n* = 126). Most entries were classified in the source report as written by users describing their own migraine-related concerns (91%), while 9% were classified as caregiver-generated entries, primarily from parents, children, or spouses. Key stages, user-reported experiences, core user concerns, and their clinical implications are summarized in Table 1. All counts and percentages in the social listening findings are retained as descriptive outputs from the Kimola source report and were not independently recalculated or verified by the authors. Unless otherwise specified, subcategory and tag-level percentages refer to distributions within the corresponding parent category rather than to the total dataset.

**Table 1:** Key stages and clinical implications of the digital migraine journey.

Stage	Key user-reported experience	Digital manifestations	Clinical implications
Pre-treatment phase	• Online symptom research • Headache-types confusion • Self-interpretation of symptoms • Diagnostic uncertainty • MRI or testing concerns	• Searches for migraine symptoms, triggers, severity, and causes • Repeated online checking of diagnostic criteria, MRI* indications, and test explanations	• Assess pre-consultation beliefs • Address misconceptions • Clarify diagnostic criteria • Explain imaging indications • Provide reliable educational resources
Treatment phase	• Side effects concerns • Perceived lack of benefit • Uncertainty about continuation or switching	• Searches comparing treatments, adverse effects, perceived inefficacy, and discontinuation or switching	• Set realistic expectations • Address side effects • Support treatment continuity • Use shared decision-making
Disease experience	• Physical burden, including visual symptoms and severe pain • Psychological distress and fear of recurrence • Social and functional burden	• Entries describing aura-like symptoms, menstrual triggers, pain burden, psychological distress, and functional limitations	• Assess migraine-related disability • Address psychological and functional burden • Consider psychological comorbidity when appropriate • Provide individualized support
Post-treatment phase	• Discontinuation after insufficient benefit or symptom improvement • Recurrent symptoms after stopping treatment • Search for longer-lasting solutions • Interest in complementary or procedural options	• Searches about stopping treatment, recurrence, and non-oral, procedural or complementary options	• Explore reasons for discontinuation • Reassess diagnosis and treatment goals • Discuss evidence-based alternatives • Differentiate evidence-based procedural or adjunctive options from non-evidence-based approaches • Maintain continuity of care

*MRI, magnetic resonance imaging.

#### 3.1.1 Pre-treatment phase

The pre-treatment phase comprised the largest proportion of user-generated content reported in the Kimola source report as 56% of the final dataset (*n* = 1,379/2,500). Within this phase, entries were grouped into two main thematic domains: an information-seeking domain reported as 71% of pre-treatment entries (*n* = 979/1,379), primarily centered on symptom interpretation and treatment-related inquiries, and a diagnostic skepticism domain, reported as 29% of pre-treatment entries (*n* = 400/1,379), involving diagnostic test interpretation and questioning of prior diagnoses (Figure 1).

**Figure 1:**
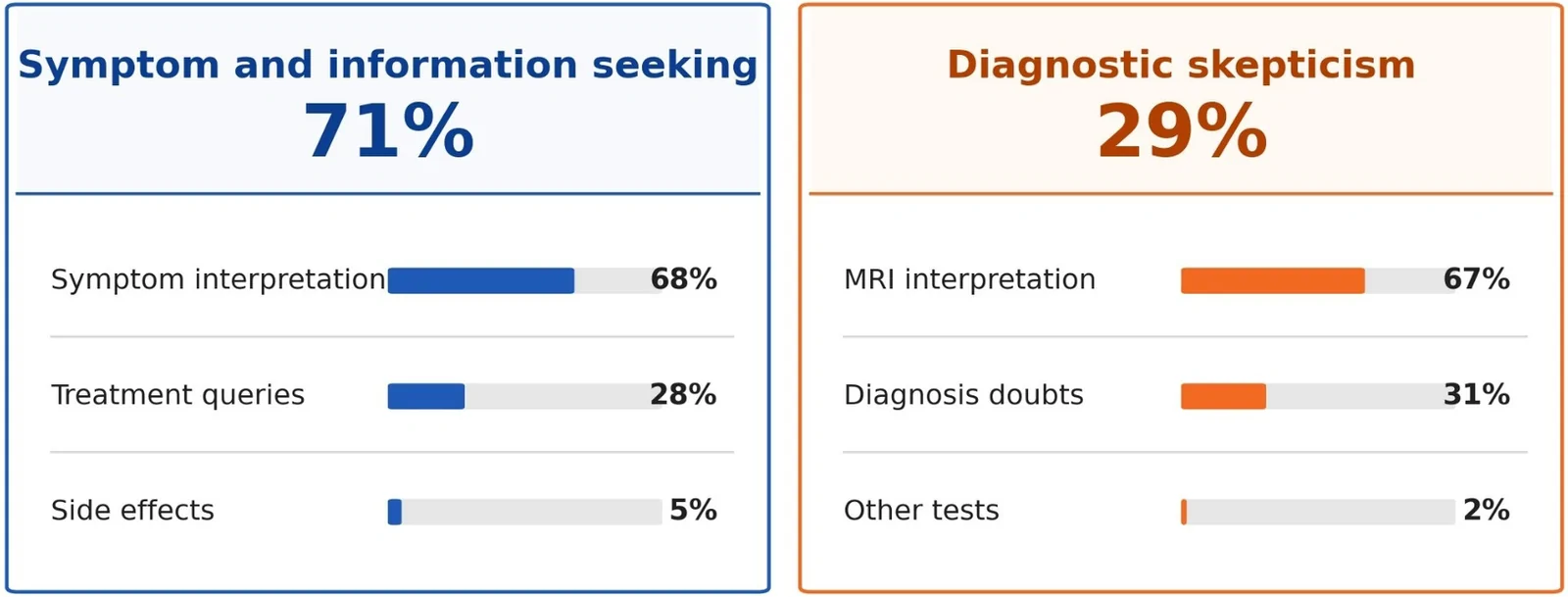
Pre-treatment phase: distribution of user-generated entries. Percentages may not sum to 100% because of rounding. MRI, magnetic resonance imaging.

Within the information-seeking domain (*n* = 979), most entries involved exploratory online searches related to migraine symptoms, reported as 68%. Users frequently attempted to determine whether their symptoms were consistent with migraine and often expressed uncertainty about whether migraine was a distinct neurological condition or a more general type of headache. Misinterpretation of symptoms—particularly visual and sensory features—appeared as a recurring theme. This uncertainty was reflected in an illustrative translated excerpt included in the Kimola source report: “Is migraine a disease or just a sign of something worse?”

Treatment-related concerns were also observed within the information-seeking domain. In this domain, 28% of entries explored available treatment or medication options, while 5% focused more specifically on potential side effects. Concerns about cognitive effects, weight gain, and long-term safety were repeatedly mentioned. In addition, reluctance toward pharmacological treatments and a preference for non-drug approaches emerged as recurring patterns. Some entries also described seeking medical care only after symptoms became more severe.

Within the diagnostic skepticism domain (*n* = 400), 67% of entries involved magnetic resonance imaging (MRI) result interpretation, 31% questioned the accuracy of physician diagnosis, and 2% concerned other diagnostic test results. These entries suggest that online platforms were often used by users to seek reassurance or a second opinion regarding migraine-related symptoms or diagnostic information.

#### 3.1.2 Treatment phase

The treatment phase accounted for approximately 27% of the final source-report dataset (*n* = 648/2,500) and captured user-reported experiences related to treatment effectiveness, safety, and tolerability (Figure 2). Entries in this category reflected interactions between treatment expectations, perceived treatment outcomes, and the use of digital sources for clarification.

**Figure 2:**
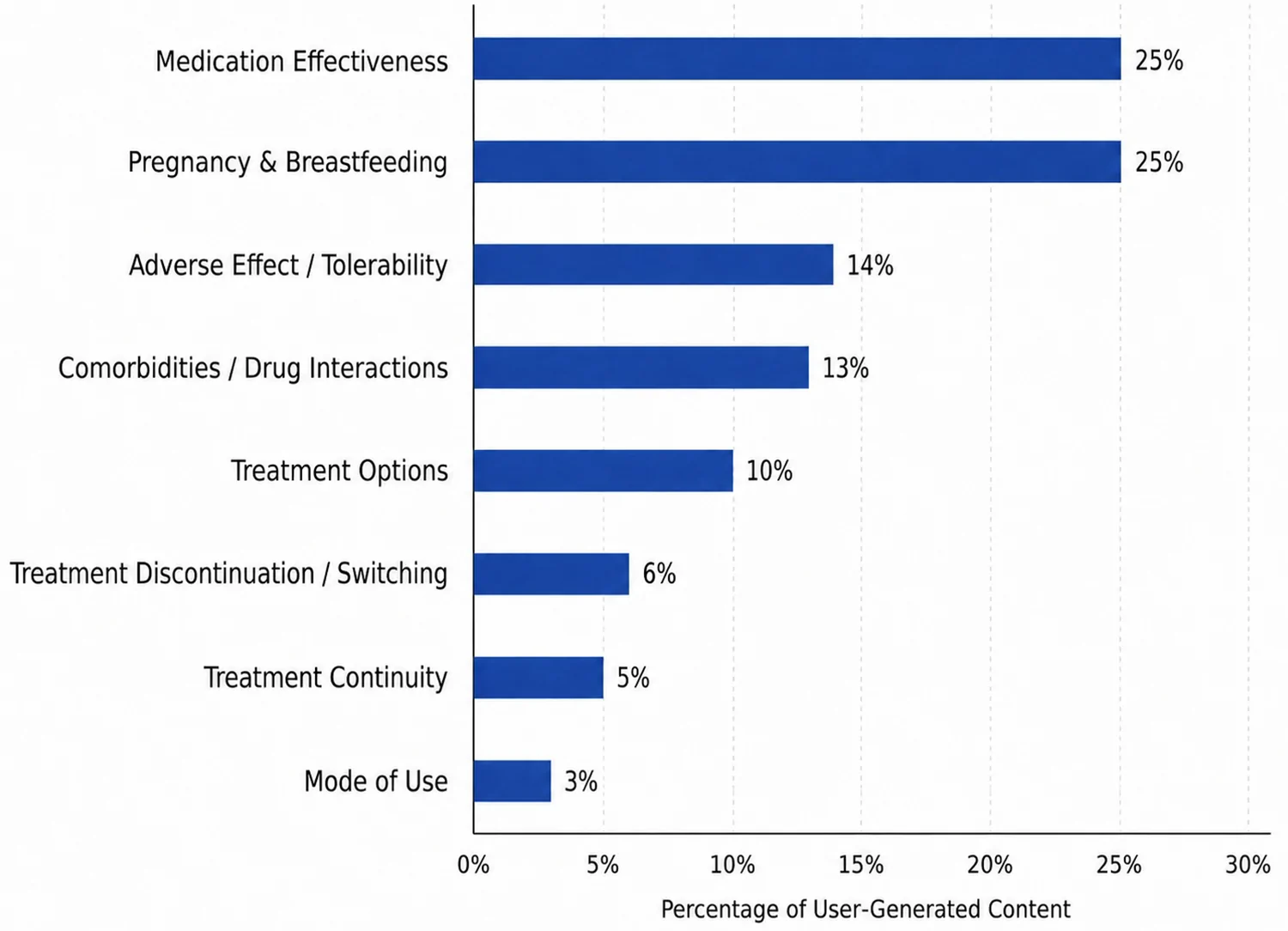
Treatment phase: distribution of user-generated entries. Percentages may not sum to 100% because of rounding.

Medication-effectiveness concerns were one of the most frequent treatment-phase topics, reported as 25% of treatment-phase entries (*n* = 158). Within this subcategory, the source report indicated that most entries referred to perceived ineffectiveness. Many users described persistent symptoms despite treatment, and partial or delayed improvement was often interpreted as treatment failure. This pattern suggested that expectations of rapid or complete relief were common among users discussing treatment response. This uncertainty was reflected in an illustrative translated excerpt included in the Kimola source report: “How long should I wait for the medication to take effect?”

Treatment safety was another prominent concern within the treatment phase category. Pregnancy and breastfeeding discussions were reported as 25% of treatment-phase entries and most frequently concerned medication use during pregnancy, postpartum, or breastfeeding periods, with users seeking clarification about which treatments could be used safely. Some entries also linked reproductive health considerations with migraine treatment concerns, including questions around contraceptive choices and migraine-specific treatment options.

Adverse effects and tolerability concerns were reported as 14% of entries. Reported problems included fatigue, cognitive slowing, impaired concentration, and reduced daily functioning. These concerns were particularly relevant in entries from working-age users, for whom cognitive performance and functional capacity appeared central to treatment acceptability. Overall, medication-effectiveness concerns, pregnancy- or breastfeeding-related safety concerns, adverse effects, comorbidities or drug interactions, treatment discontinuation or switching, treatment continuity, and treatment options were recurring treatment-phase topics in the source report.

#### 3.1.3 Disease experience

The disease experience phase accounted for approximately 13% of the final source-report dataset (*n* = 347/2,500) and reflected the broader burden of migraine beyond treatment-specific questions (Figure 3). Within this phase, most entries focused on physical burden, reported as 69% of disease experience entries. The physical domain included visual disturbances, menstrual triggers, severe pain, muscle contraction or tremor, sensory or hearing disturbances, and other physical concerns. Psychological burden was reported in 23% of entries within this phase and included stress and anxiety, sleep or balance disturbances, and cognitive difficulties. A smaller proportion of entries, reported as 8%, described social and functional consequences, including reduced work performance, limitations in daily activities, and disruption of family responsibilities.

**Figure 3:**
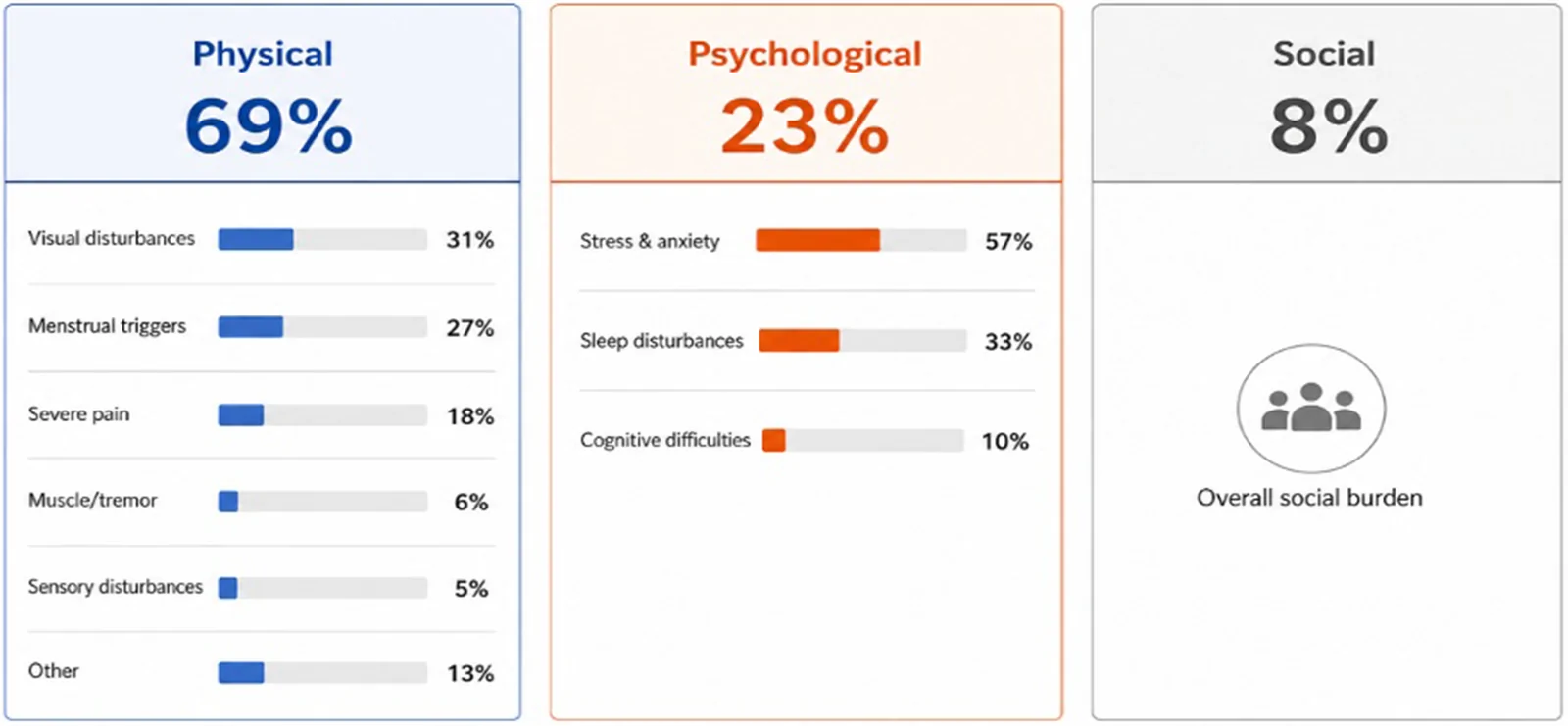
Disease experience: distribution of user-generated entries. Percentages within subthemes are shown within each domain.

Menstrual triggers were among the recurring physical themes and were frequently discussed in relation to professional, domestic, or daily responsibilities. Source-report insights also described fear of severe neurological consequences and concerns related to recurrent or unpredictable attacks. Overall, migraine-related entries in this phase captured not only episodic pain, but also ongoing functional and psychological vulnerability.

#### 3.1.4 Post-treatment phase

The post-treatment phase represented a small proportion of the final source-report dataset, presented in the Kimola source- report as approximately 4% of entries (*n* = 126/2,500), and provided insight into longer-term treatment-related discussions (Figure 4). Within this phase, the most frequent pattern was interest in options beyond conventional oral pharmacotherapy, reported in approximately 60% of post-treatment entries. These entries included interest in complementary approaches, such as acupuncture and neural therapy, as well as evidence-based procedural or adjunctive options used in migraine care, including onabotulinumtoxinA. According to the source-report interpretation, this pattern was mainly observed in entries describing insufficient benefit from conventional treatments or recurrence after partial improvement. The source-report also noted references to social networks, anecdotal recommendations, or digital content when users discussed treatment options.

**Figure 4:**
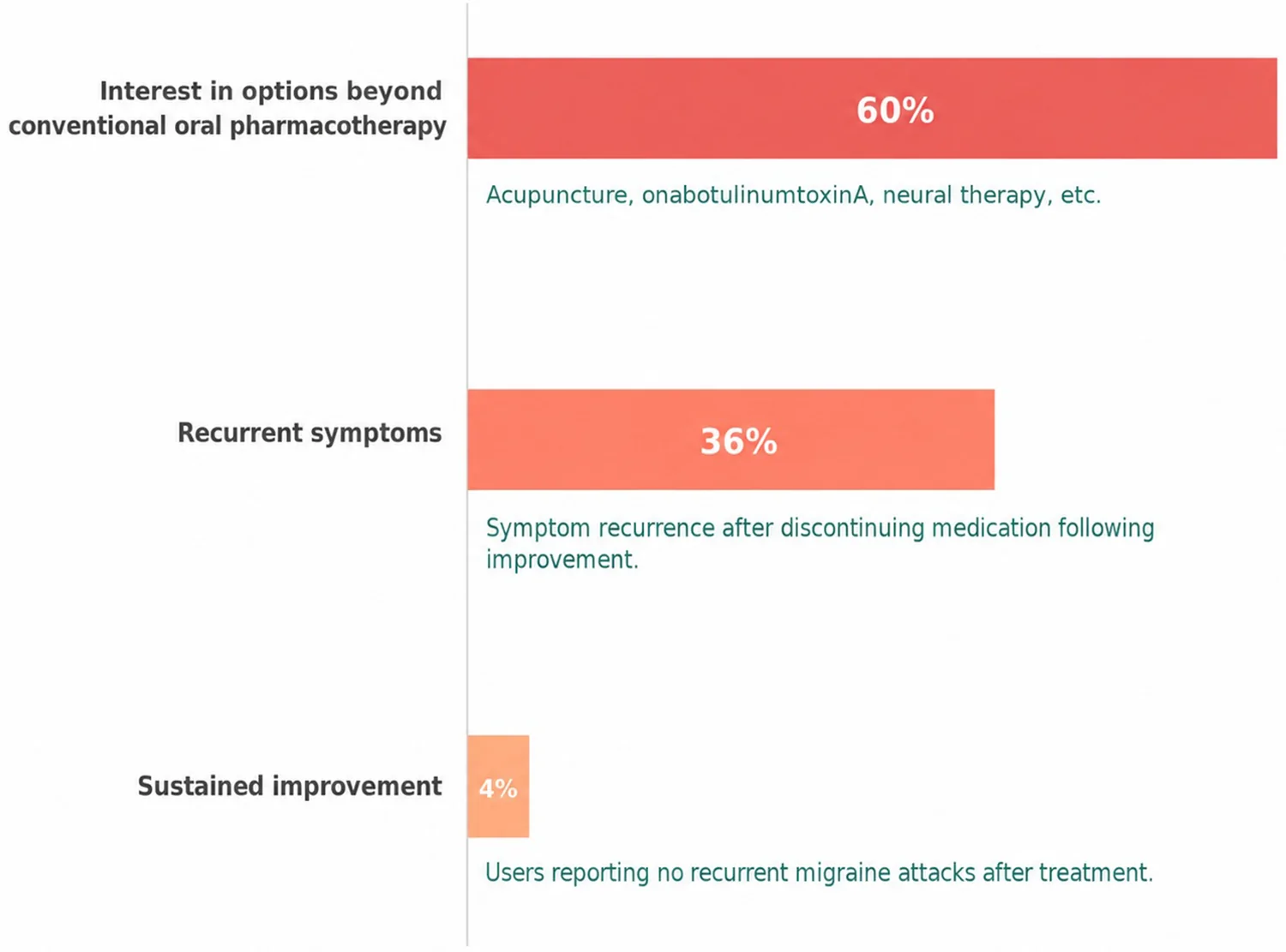
Post-treatment phase: distribution of user-generated entries. Percentages show the distribution of post-treatment entries by theme.

In addition, 36% of post-treatment entries described recurrent symptoms after discontinuing medication following symptom improvement. These entries suggested uncertainty about treatment continuation after short-term symptom relief rather than providing evidence of objectively measured discontinuation behavior. This was reflected in a source-report insight such as: “Once the headaches stopped, I quit the medication, but now they are back.” A small proportion of post-treatment entries, reported as 4%, described sustained improvement, with users reporting no recurrent migraine attacks after treatment.

### 3.2 Physician survey findings

A total of 10 neurologists with expertise in migraine and headache disorders participated in the survey. Given the small expert sample, physician survey findings are reported as raw counts rather than percentages and should be interpreted as expert-informed clinical perspectives, not as representative estimates of neurologists’ views in Türkiye. Seven of 10 neurologists agreed or strongly agreed that many of their patients use social media as a major source of migraine-related information. Seven of 10 agreed that migraine-related information shared on social media is often inaccurate or misleading, while 3 of 10 were neutral. All 10 neurologists agreed or strongly agreed that structured, evidence-based digital education platforms led by headache experts are needed.

All 10 neurologists agreed or strongly agreed that social media may shape patients’ expectations regarding treatment outcomes. When asked which aspects of the migraine experience were perceived to be most affected by social media content, all 10 selected the use of non-pharmacological or alternative therapies, 6 of 10 selected expectations regarding treatment efficacy, 5 of 10 selected awareness of newer treatment options, and 3 of 10 selected choice of preventive treatment. When asked to estimate the proportion of their patients whose treatment expectations, adherence, or symptom management may be affected by social media, 4 of 10 selected 26%–50% and 3 of 10 selected 51%–75%.

Misinformation was identified as a key perceived challenge. Nine of 10 neurologists agreed or strongly agreed that misinformation on social media complicates physician–patient communication and poses a barrier to appropriate patient care. The open-ended responses further emphasized that misleading information, particularly when presented by non-expert sources, may shape patients’ perceptions and highlighted the need for greater involvement of headache specialists in digital spaces.

Neurologists acknowledged both benefits and risks of social media in migraine care. The leading perceived benefits were reducing stigma and misconceptions, selected by all 10 neurologists; increasing public awareness, selected by 9 of 10; providing peer support and community, selected by 9 of 10; helping patients identify reliable treatment information, selected by 8 of 10; and facilitating physician–patient communication, selected by 7 of 10. The main perceived risks were the spread of misinformation regarding migraine treatments, increased interest in non-evidence-based treatment recommendations or unsupported alternative approaches, and delayed professional care-seeking, each selected by all 10 neurologists. Heightened anxiety and misperceptions were selected by 8 of 10 neurologists.

A consolidated summary linking the main themes derived from user-generated digital entries, corresponding neurologist survey findings, and clinical relevance is provided in Table 2.

**Table 2:** Summary of themes derived from user-generated digital entries, neurologist survey findings, and clinical relevance.

Main domain	Themes derived from user-generated digital entries	Neurologist survey finding	Clinical relevance
Online information-seeking	Pre-treatment entries frequently reflected symptom interpretation, treatment inquiries, and diagnostic uncertainty.	7/10 neurologists agreed or strongly agreed that many patients rely on social media as a major source of migraine information.	Online information-seeking should be proactively addressed during consultations.
Misinformation and communication	User-generated entries included uncertainty about diagnosis, treatment effects, safety, and alternative approaches.	7/10 neurologists agreed that migraine-related information on social media is often inaccurate or misleading; 9/10 agreed or strongly agreed that misinformation complicates physician–patient communication.	Clinicians may need to identify and correct misinformation during patient encounters.
Treatment expectations	Treatment-related entries included perceived lack of efficacy, adverse effects, safety concerns, and uncertainty about continuation or switching.	10/10 neurologists agreed or strongly agreed that social media may shape patients’ expectations regarding treatment outcomes.	Expectation management should be included in migraine treatment discussions.
Non-pharmacological or alternative approaches	Post-treatment and treatment-related entries included interest in options beyond conventional oral pharmacotherapy, including complementary or procedural approaches.	10/10 neurologists selected use of non-pharmacological or alternative therapies as an aspect commonly perceived to be shaped by social media.	Digital education should clearly distinguish evidence-based, adjunctive, procedural, and non-evidence-based approaches.
Benefits of social media	Digital platforms may create opportunities for awareness, peer support, and patient engagement.	10/10 neurologists selected reducing stigma and misconceptions; 9/10 selected increasing public awareness and peer support as potential benefits.	Expert-informed digital content may support awareness and reduce stigma.
Risks of social media	Online content may reinforce uncertainty, unrealistic expectations, misinformation, and delayed care-seeking.	10/10 neurologists selected misinformation, increased use of non-evidence-based/ alternative therapies, and delayed professional care-seeking as major risks.	Structured, evidence-based, expert-led digital education is needed.
Need for expert-led digital education	User-generated entries reflected unmet information needs across the migraine care journey.	10/10 neurologists agreed or strongly agreed that structured, evidence-based digital education platforms led by headache experts are needed.	Digital migraine education should be embedded in patient-centered care pathways.

## 4 Discussion

This exploratory study narratively juxtaposed social listening findings with neurologists’ expert-informed perspectives to examine digitally expressed migraine-related concerns among users of a Turkish online health platform. The social listening findings and neurologist survey findings are interpreted together at the level of clinical meaning rather than as formally integrated datasets. Entry-derived themes primarily revealed unmet information needs, diagnostic uncertainty, treatment-related concerns, safety questions, discussions around treatment discontinuation, and interest in non-pharmacological or alternative approaches. Responses from the participating neurologists provided expert-informed clinical contextualization for these themes, particularly by highlighting the perceived role of online information in treatment expectations, the perceived relevance of misinformation to physician–patient communication, and the need for structured, expert-led digital education. Given the small purposive expert sample, these responses should not be interpreted as validation of the source-report findings or as representative evidence of neurologists’ views nationally.

Taken together, these findings suggest a perception gap in digitally mediated migraine care: platform users may use digital spaces to interpret symptoms, evaluate treatments, and seek reassurance, while clinicians may encounter digitally shaped beliefs during communication and treatment planning. In this context, digital environments may function as influential spaces within the migraine care journey among digitally engaged users. Figure 5 contextualizes these patterns through a conceptual model derived from social listening findings, translating themes derived from user-generated entries into a structured, clinically interpretable framework that highlights key care-pathway inflection points and potential clinical opportunities. Therefore, the following sections discuss the findings across clinically relevant domains: pre-treatment information needs, treatment expectation management, treatment continuation and discontinuation, misinformation, physician–patient communication, and implications for digital migraine education in Türkiye.

**Figure 5:**
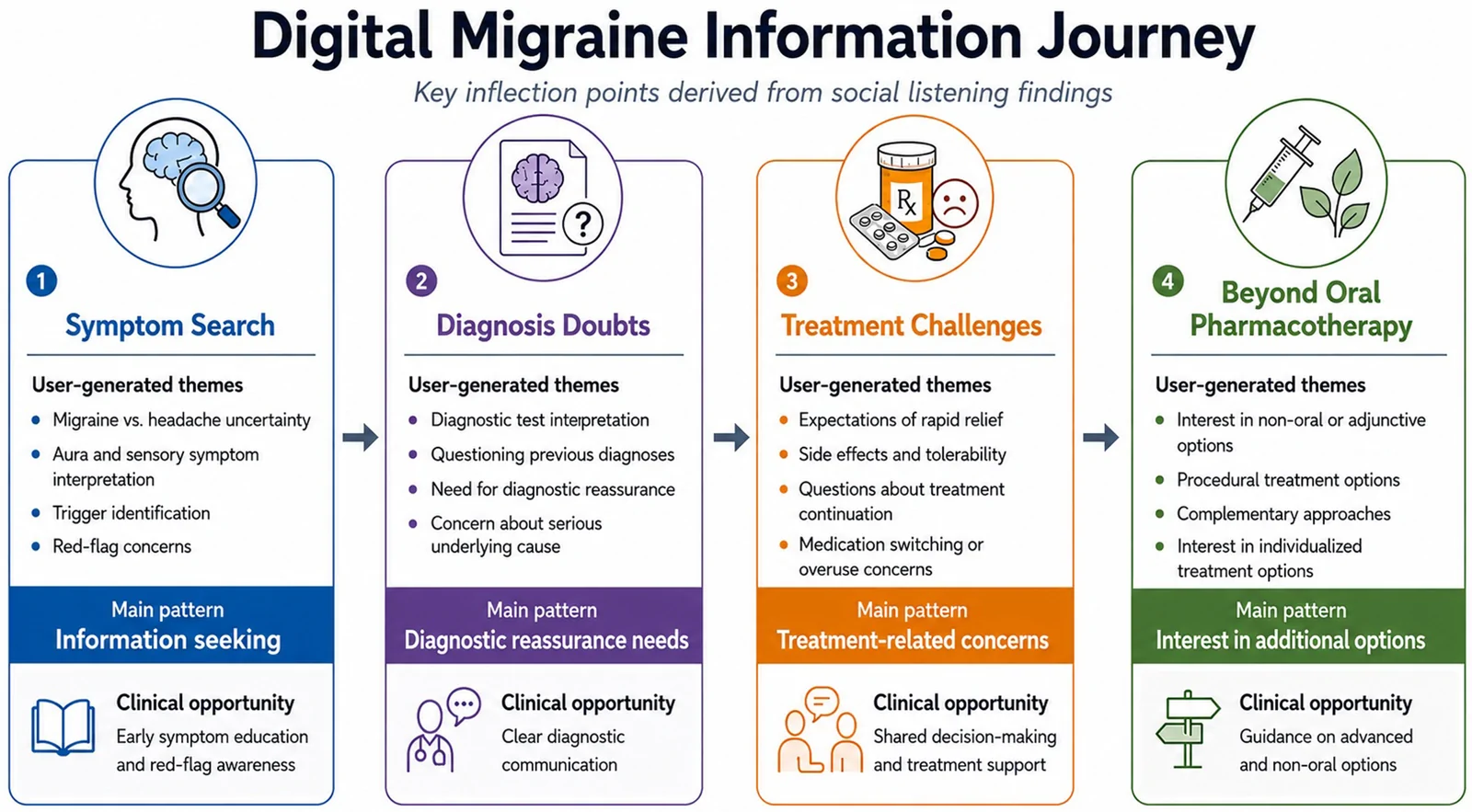
Conceptual model of the digital migraine journey derived from social listening findings.

### 4.1 The digital pre-treatment gap: high information demand and low health literacy

The pre-treatment phase was marked by intensive online information seeking, indicating a substantial unmet need for accessible and reliable migraine information. Users frequently attempted to interpret symptoms and distinguish migraine from other headache disorders, yet many entries reflected limited understanding of migraine as a distinct neurological disease. This is consistent with previous evidence showing that people commonly seek health information online before consulting healthcare professionals and that migraine-related digital discussions often focus on symptoms, triggers, and treatment options (9, 12, 16, 17).

In the present analysis, visual and sensory symptoms were sometimes interpreted as ophthalmologic problems or as indicators of more serious neurological disease. Such unguided online interpretation may contribute to uncertainty, anxiety, and delayed help-seeking (18, 19). Concerns about hereditary risk were also evident. Although such concerns are biologically plausible, given the heritable component of migraine and the identification of multiple susceptibility loci, beliefs about heredity may also shape illness perception and reinforce worry or maladaptive interpretations of recurrent symptoms (20–24).

These findings suggest a gap between information availability and health literacy. Digital environments increase access to migraine-related content, but may not consistently support accurate symptom recognition or timely consultation. In Türkiye, this gap may be amplified by limited migraine awareness at the first point of care, potentially increasing reliance on unfiltered digital sources early in the care pathway (13, 25). Targeted digital health literacy interventions are therefore needed to improve symptom recognition and guide appropriate medical consultation (26).

### 4.2 Treatment expectations and clinical realities

A central theme was the gap between treatment expectations expressed in user-generated entries and the clinical realities of migraine treatment. Entries frequently described delayed, partial, or fluctuating treatment responses as perceived treatment failure, suggesting an expectation of rapid and complete symptom resolution among some users. This aligns with previous work showing that patients often highly value rapid pain reduction, pain freedom, and durable benefit, but may have limited understanding of variability in treatment response and the iterative nature of migraine management (27–31).

Digital environments may amplify this expectation gap when treatment information is presented in simplified, anecdotal, or poorly contextualized forms. Such content may shape beliefs about how quickly therapies should work, what constitutes treatment success, and when treatment should be changed or stopped (9, 17, 30–32). Clinically, these expectations may complicate satisfaction and shared decision-making (31, 33).

### 4.3 Treatment continuation, discontinuation, and interest beyond conventional care

The post-treatment phase included entries describing treatment discontinuation after symptom improvement, often followed by recurrence. Rather than indicating a simple rejection of treatment, these entries may reflect the difficulty of sustaining long-term preventive care in a chronic disorder that requires persistence, treatment adjustment, and realistic expectation-setting. This interpretation is supported by real-world data showing poor persistence with preventive migraine therapies, particularly within the first 6–12 months; in the PERSEC study, only 32.4% and 30.4% of patients remained persistent at 6 and 12 months, respectively, while European OVERCOME data similarly showed limited satisfaction and problematic preventive treatment patterns (34, 35). Turkish real-world data evaluating continuity and withdrawal among patients receiving onabotulinumtoxinA for chronic migraine are consistent with this pattern (36).

In parallel, many entries described interest in options beyond conventional oral pharmacological treatment, including complementary or non-pharmacological approaches as well as procedural or adjunctive therapies, when conventional treatments were perceived as ineffective or poorly tolerated. This is consistent with evidence showing frequent use of complementary and integrative approaches in migraine and with observations that peer narratives in digital communities may reinforce perceptions of these approaches as more natural or safer than prescription therapies (37–39). These findings suggest not necessarily rejection of conventional care, but an ongoing search for options perceived as effective, tolerable, and sustainable.

This ambiguity has practical implications. Patient education should clearly distinguish evidence-based migraine-specific and procedural therapies from complementary approaches with limited or uncertain evidence. However, patient-facing information about migraine treatment options remains uneven online, where treatment discourse is often shaped by personal experience rather than balanced explanation of indications, benefits, limitations, and access considerations (9). This highlights the importance of balanced counseling on the evidence level, safety profile, expected benefit, and appropriate place of each treatment option within migraine care.

### 4.4 Digital environments as a double-edged information ecosystem

Entry-derived findings and neurologist survey responses suggest that digital environments may play a dual role in migraine care. They may improve access to information, increase awareness, and provide peer support for a condition that is often invisible and episodic. At the same time, they may amplify misinformation, oversimplified explanations, and non-evidence-based treatment messages, thereby complicating symptom interpretation and care discussions (7, 11, 17).

This issue extends beyond a single platform and reflects a heterogeneous digital ecosystem in which migraine-related perceptions may be shaped by websites, online forums, social media platforms, videos, and patient-facing health portals (9). Prior studies show that the quality of migraine-related online videos, websites, and Turkish-language resources varies substantially, ranging from authoritative educational material to incomplete or misleading content (25, 40–42). Short-form visual content may further shape disease perception in ways that are not always clinically accurate (43–45). Overall, these findings support addressing digital information quality as part of migraine care, rather than treating online information seeking as separate from clinical practice.

### 4.5 The patient–physician perception gap

These findings collectively point to a patient–physician perception gap in digitally mediated migraine care. Some patients may enter the clinical encounter feeling informed and proactive, while physicians may encounter expectations shaped by incomplete, inaccurate, or poorly contextualized information. The challenge is therefore not only misinformation itself, but also the way digitally formed beliefs may affect trust, communication, and treatment alignment.

Previous studies suggest that online health information may support engagement and shared decision-making, but may also increase anxiety, confusion, and tension when health literacy is limited or online findings are not discussed during consultation (18, 46, 47). This dynamic is particularly relevant in migraine, where management depends on careful discussion of symptoms, disability, treatment goals, comorbidities, and iterative treatment optimization. Migraine-specific studies suggest that such conversations are not always sufficiently developed in routine care, with chronic migraine, treatment planning, and patient concerns sometimes under-discussed (48–50).

In Türkiye, this gap may be reinforced by limited migraine awareness at the primary-care level and by variable reliability and readability of online migraine-treatment information (13, 46). However, digital exposure does not necessarily undermine the physician–patient relationship. When addressed constructively, it may create opportunities for clearer expectation management, more explicit discussion of treatment goals, and more collaborative decision-making. Bridging this gap requires clinicians to explicitly invite discussion of online information, clarify treatment goals, and address misconceptions within a shared decision-making framework (47, 51).

### 4.6 Digital health in migraine care: barriers and practical implications

The present findings suggest that digital environments should not be considered entirely separate from the migraine care journey, particularly among digitally engaged users. They may intersect with symptom interpretation, treatment expectations, medication-related concerns, continuity of care, and physician–patient communication. Digital platforms should also not be viewed solely as sources of misinformation. When clinically guided and appropriately structured, they may support education, symptom monitoring, care coordination, and engagement among people seeking migraine-related information. Their value, however, depends on content quality, clinical oversight, user health literacy, and alignment with clinical care pathways.

Several barriers remain important in the digital era. First, migraine may still be perceived as a nonspecific or benign headache rather than a disabling neurological disease, which may be associated with delayed help-seeking or delayed access to appropriate care. Second, unguided online information seeking may reinforce diagnostic uncertainty, medication-related concerns, unrealistic expectations, and interest in non-evidence-based or insufficiently supported approaches. Third, limited access to headache expertise, complex referral pathways, inadequate follow-up, and insufficient attention to comorbidities such as anxiety, depression, and sleep disorders may further compromise care. Evidence from referral data, telemedicine reviews, and studies of migraine comorbidity supports the need for more coordinated and multidisciplinary care models (14, 52, 53). These issues may be particularly relevant in Türkiye, where internet use is widespread but migraine awareness, first-contact care, and the reliability of online information vary (13, 25, 54). Therefore, digital influences should be considered not only as individual information-seeking behaviors, but also in relation to health-system capacity, referral structures, and clinical communication.

In response to these barriers, practical priorities include developing expert-led and locally adapted digital content to improve migraine literacy, and support informed treatment expectations. Stronger engagement of neurologists and professional societies on digital health platforms may help improve the quality of migraine-related information and reduce the visibility of unverified or misleading recommendations. Practical approaches may include evidence-based frequently asked question modules, user-facing explanations of diagnosis and red-flag symptoms, balanced treatment summaries, digital health literacy materials, and clear differentiation between evidence-based migraine therapies and non-evidence-based or insufficiently supported approaches (7, 10, 11, 25, 40–45). For platforms that allow user questions and medical responses, credential verification for responding clinicians, periodic content review, and evidence-based user-facing responses may help improve information quality. Universal expert review before publication of every individual user entry may not be feasible; however, additional expert oversight may be particularly important for clinically sensitive topics such as medication safety, pregnancy or lactation, treatment discontinuation, off-label treatment discussions, red-flag symptoms, and non-evidence-based interventions.

These strategies should complement, not replace, individualized clinical decision-making and may be integrated with telemedicine, electronic headache diaries, digital intake tools, remote monitoring, and app-supported follow-up to improve engagement, continuity, and communication in migraine care (55–60). The proposed action domains summarized in Figure 6 may therefore be considered components of a coordinated, person-centered strategy to improve migraine care in the digital era.

**Figure 6:**
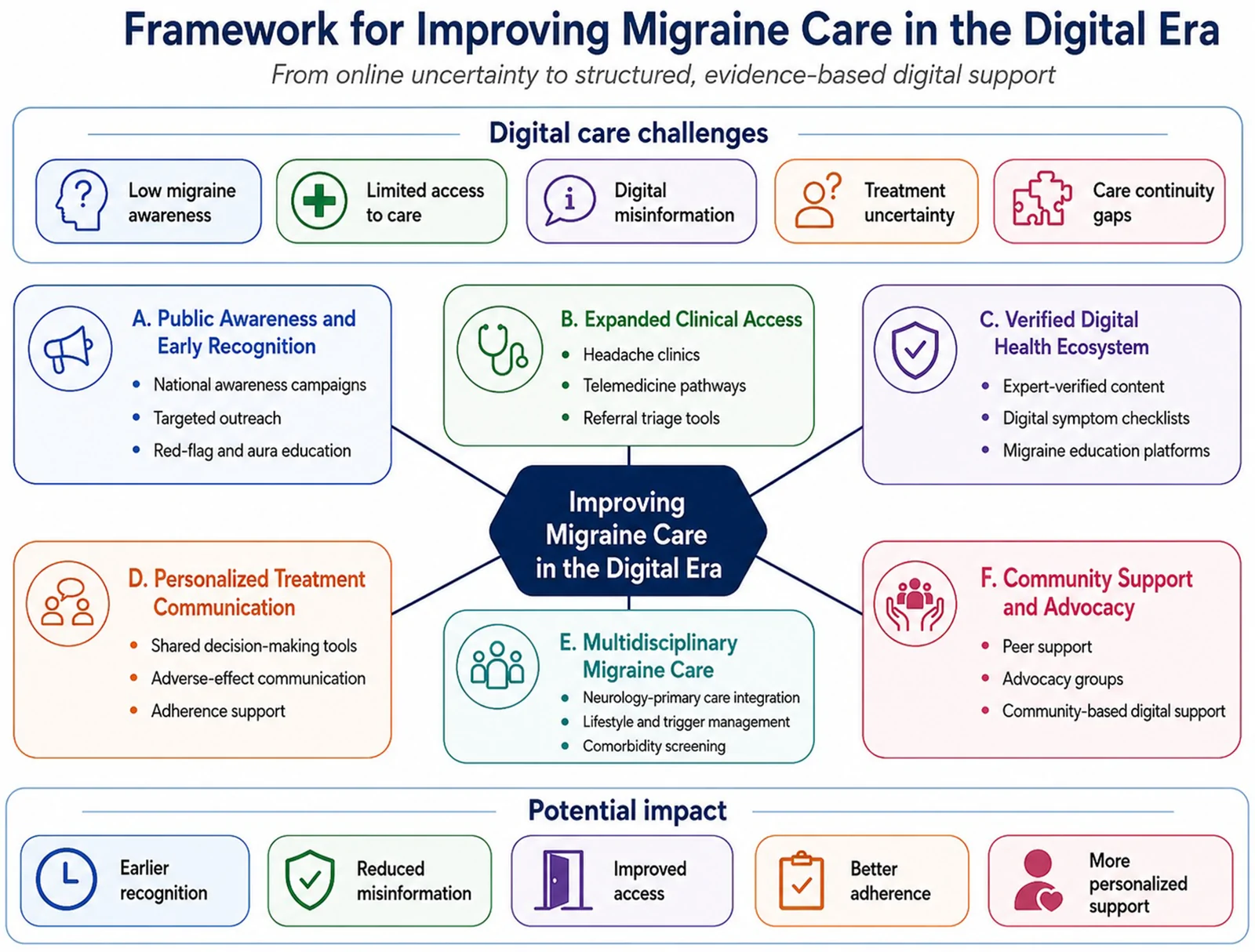
Framework for improving migraine care in the digital era. Note: Percentages shown in Figures 1–4 are descriptive source-report outputs from the Kimola social listening report and were not independently verified by the authors.

### 4.7 Implications for migraine care in Türkiye

In the Turkish context, these findings should be considered alongside increasing national efforts to improve migraine awareness, education, and care pathways. Recent initiatives, including national migraine awareness activities, patient-oriented educational resources, workplace-focused awareness programs, pharmacist education initiatives, and the development of migraine-related clinical guidance, suggest that migraine is increasingly being addressed not only as a neurological disorder but also as a public health, communication, and socioeconomic issue (61–68). In this setting, themes derived from user-generated digital entries may provide complementary insight into unmet information needs, treatment expectations, and misconceptions that are not always fully captured in clinical encounters. Importantly, this national contextual relevance should not be interpreted as population-level representativeness. Rather, the findings highlight how digitally expressed migraine-related concerns may inform patient education, digital health communication, and clinical expectation management in Türkiye. The next step is to evaluate national and digital education initiatives systematically, with measurable outcomes including public awareness, access to care, quality of clinical management, treatment persistence, and patient-reported outcomes.

### 4.8 Strengths and limitations

This study has several strengths. First, it contextualizes user-generated digital entries through neurologists’ expert-informed perspectives, enabling a clinically contextualized assessment of digitally expressed migraine-related concerns and their potential relevance to migraine care. Second, the inclusion of neurologists from different geographical regions and healthcare settings in Türkiye increased the contextual relevance and diversity of the expert input. Third, the social listening component captured concerns, expectations, and treatment-related discussions expressed in naturalistic online settings, which may not be fully identified through conventional surveys.

Several limitations should be acknowledged. The social listening data were obtained from a single Turkish online health platform and reflect the concerns of digitally engaged users who posted migraine-related questions or experiences. Therefore, the findings should not be interpreted as representative of all individuals with migraine in Türkiye, particularly those who do not seek health information online, those with limited digital literacy, or those using other online environments. The unit of analysis was the individual online entry/post rather than the individual platform user; therefore, distinct posts or questions submitted by the same platform user, if present, may have contributed separately to the entry-level distributions. Accordingly, the reported distributions should be interpreted as entry-level patterns rather than proportions of unique individuals or unique platform users. The social listening component was based on secondary interpretation of a proprietary source report. Although additional methodological clarification was obtained from Kimola, the authors did not have direct access to the raw online entries, original preprocessing records, or original coding files. Therefore, independent validation of data cleaning, coding decisions, and theme generation was not possible. In addition, no formal inter-coder reliability assessment was available for the source-report coding process. The long source-report time window from 2007 to 2023 also introduces temporal heterogeneity, because migraine treatment options, patterns of platform use, and the broader digital health information environment changed substantially during this period. Therefore, findings should be interpreted as aggregated source-report patterns across the available time window rather than as evidence of temporal trends. Overall, the social listening findings should be interpreted as exploratory and hypothesis-generating.

The physician survey included a small purposive sample of neurologists and should be considered an expert-informed perspective rather than a generalizable representation of all clinicians involved in migraine care. Selection bias may also be present, as both platform users who posted online content and survey participants may represent more engaged or motivated individuals. Although the neurologist sample included participants from different geographical regions and healthcare settings, rural-specific neurologist representation was not systematically targeted. Several participating centers serve as tertiary or regional referral hospitals and may therefore reflect clinical experience with patients referred from surrounding and less-resourced areas, including rural or semi-rural settings; nevertheless, the survey may not fully capture the perspectives of neurologists practicing primarily in rural settings, where digital access, health-information behavior, referral pathways, and access to headache expertise may differ.

Although the findings are contextually grounded in Türkiye, their national relevance should not be interpreted as national representativeness. Nevertheless, several themes identified in this study, including online symptom interpretation, diagnostic reassurance-seeking, medication-related concerns, treatment expectations discussed in relation to online content, interest in non-pharmacological or alternative approaches, and the perceived relevance of misinformation to clinical communication, may be relevant to similar digitally mediated healthcare contexts beyond Türkiye. The extent of such transferability is likely to depend on healthcare access, specialist availability, reimbursement structures, digital literacy, and the credibility of online health information platforms. Despite these limitations, the study offers an exploratory framework for understanding how digital environments may intersect with migraine care and patient–clinician communication in Türkiye.

## 5 Conclusion

By narratively juxtaposing themes derived from user-generated digital entries with neurologists’ expert-informed perspectives, this study provides an exploratory and clinically contextualized framework for understanding how digital environments may intersect with migraine care beyond traditional healthcare settings. The findings suggest that digital platforms may support awareness, access to information, peer support, and patient engagement, but may also be associated with misinformation, unrealistic treatment expectations, medication-related concerns, and challenges in care continuity.

This digitally mediated perception gap highlights the need for a more patient-centered and digitally informed approach to migraine management. Priorities include strengthening digital health literacy, improving access to evidence-based migraine information, expanding expert-led digital education, and aligning digital tools with clinical care pathways. When clinically guided and appropriately structured, digital ecosystems may help make migraine care more accessible, informed, collaborative, and sustainable.

## Data Availability

The original contributions presented in the study are included in the article/Supplementary Material, further inquiries can be directed to the corresponding author.

## Supplementary material

Supplementary Data Sheet 1 Original Turkish Kimola social listening report.

Supplementary Data Sheet 2 English summary of the Kimola social listening report.

Supplementary Data Sheet 3 Physician survey questionnaire and aggregated responses.
