# Supplementary material for: Migraine care in the digital era: social listening insights and neurologists’ perspectives from Türkiye
Source: Front Digit Health. 2026 Sep 14;8:1891640. doi: 10.3389/fdgth.2026.1891640 (PMC13617366; doi:10.3389/fdgth.2026.1891640)
Supplement: Supplementary file 1 [file Datasheet1.pdf]

Migren Araştırması

Haziran, 2023

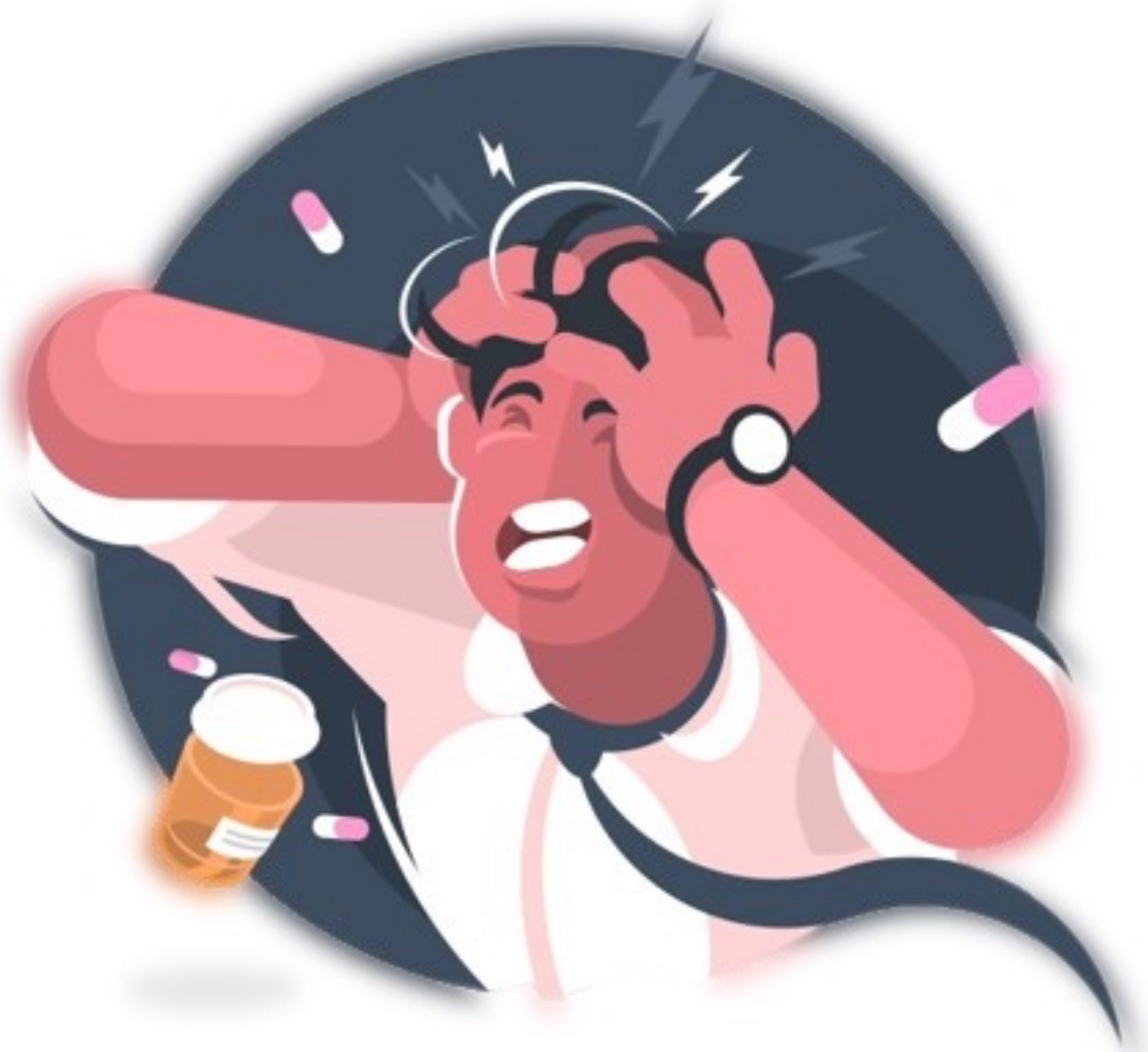

VERİNİN TOPLANMASI

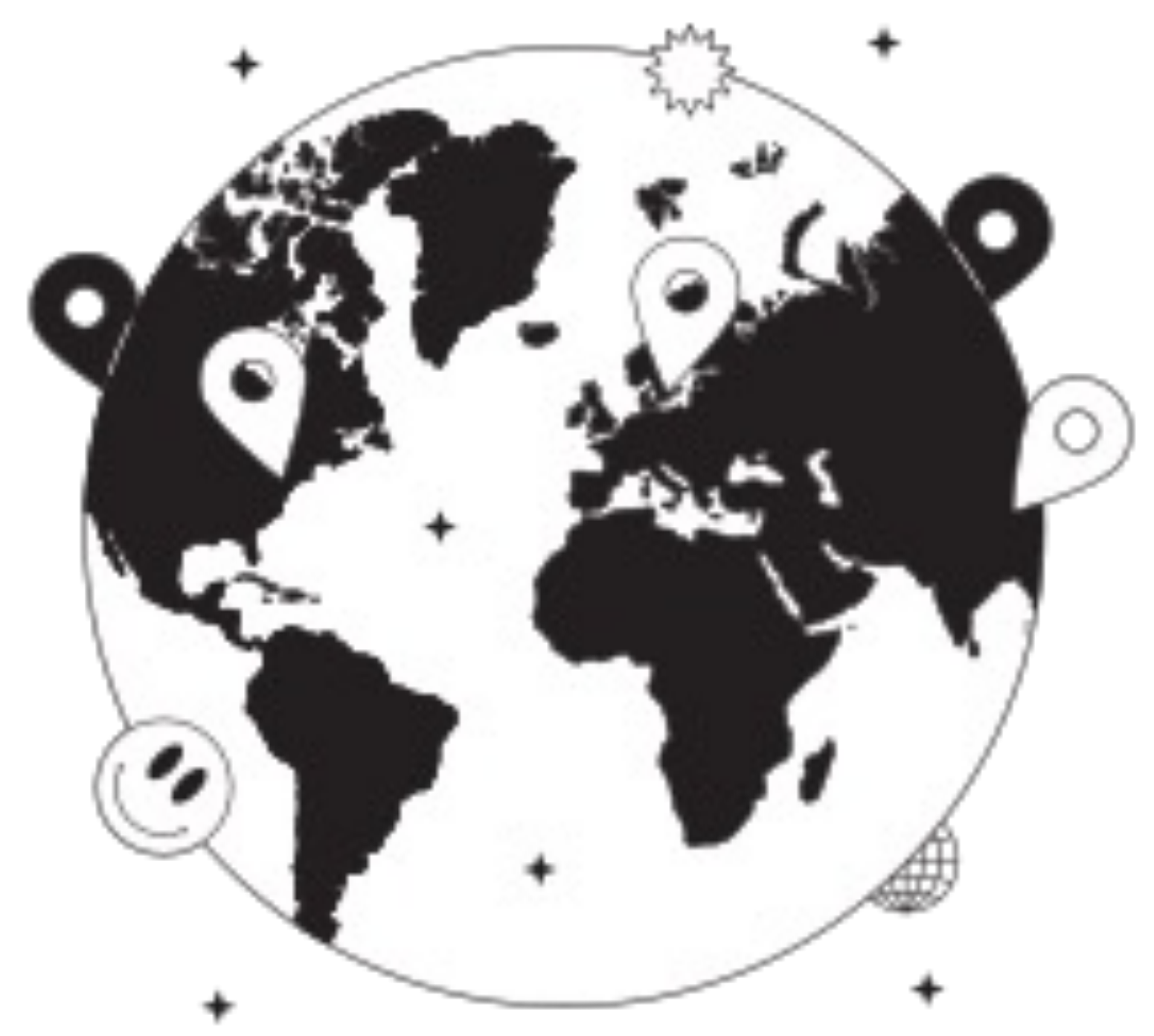

Pfizer için Kimola tarafından hazırlanan migren odaklı araştırma için hastaların doğrudan hekimlere soru sorup cevap alabildikleri bir site olan doktorsitesi.com üzerinde, 11 farklı branşta migren hakkında sorulan soruları içeren 2.500 veri toplandı. Bu veriler, sosyal araştırma çerçevesinde sınıflandırılarak analiz edildi.

VERİLERİN ELENMESİ

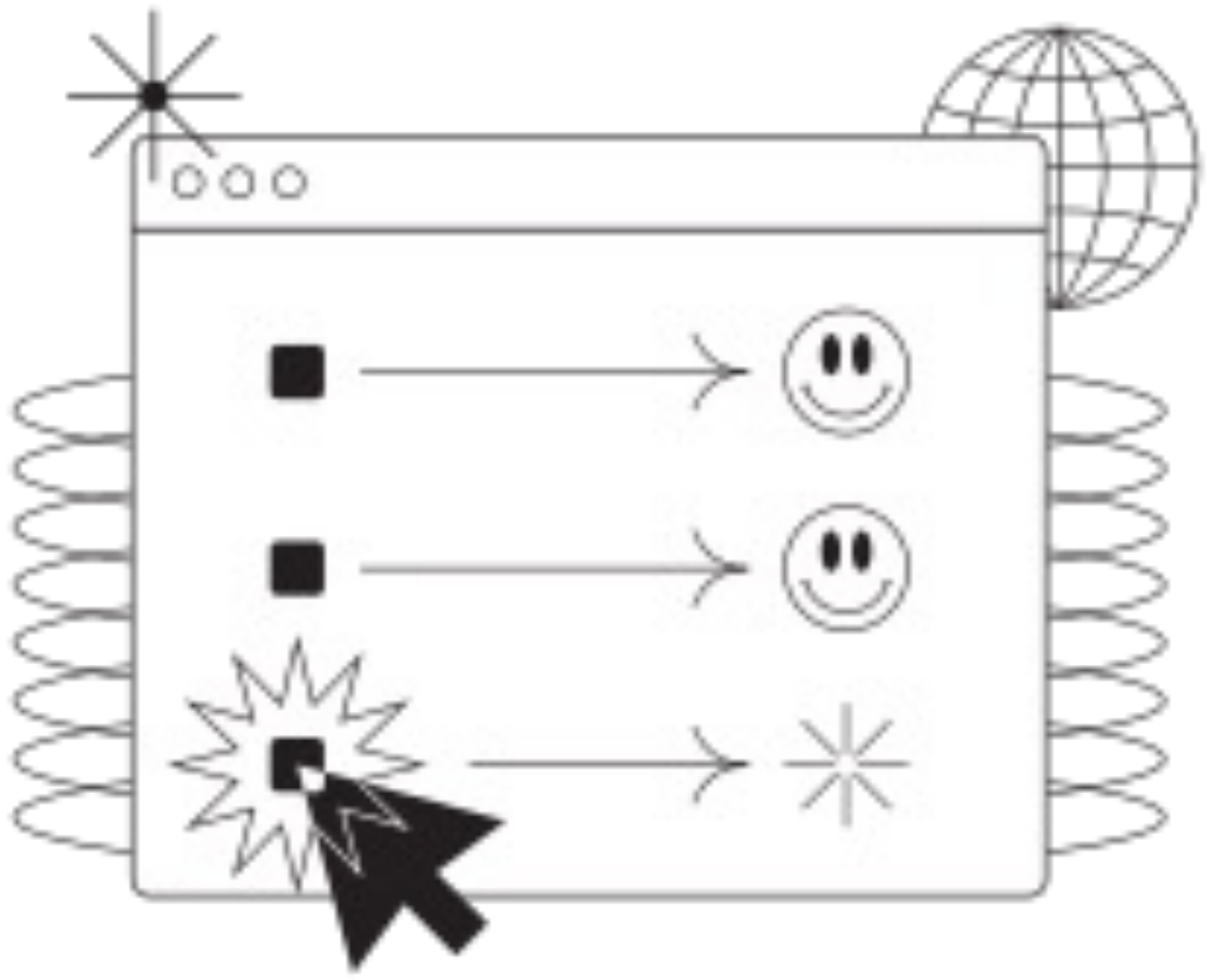

Elde edilen büyük veriden kullanılabilir bilgiye erişmek için ilgisiz verilerin elenmesinin ardından,
Pfizer Migren Araştırması 2500 veri, Kimola'nın İçgörü ve Araşatırma Ekibi tarafından sınıflandırıldı.

VERİNİN ANALİZİ

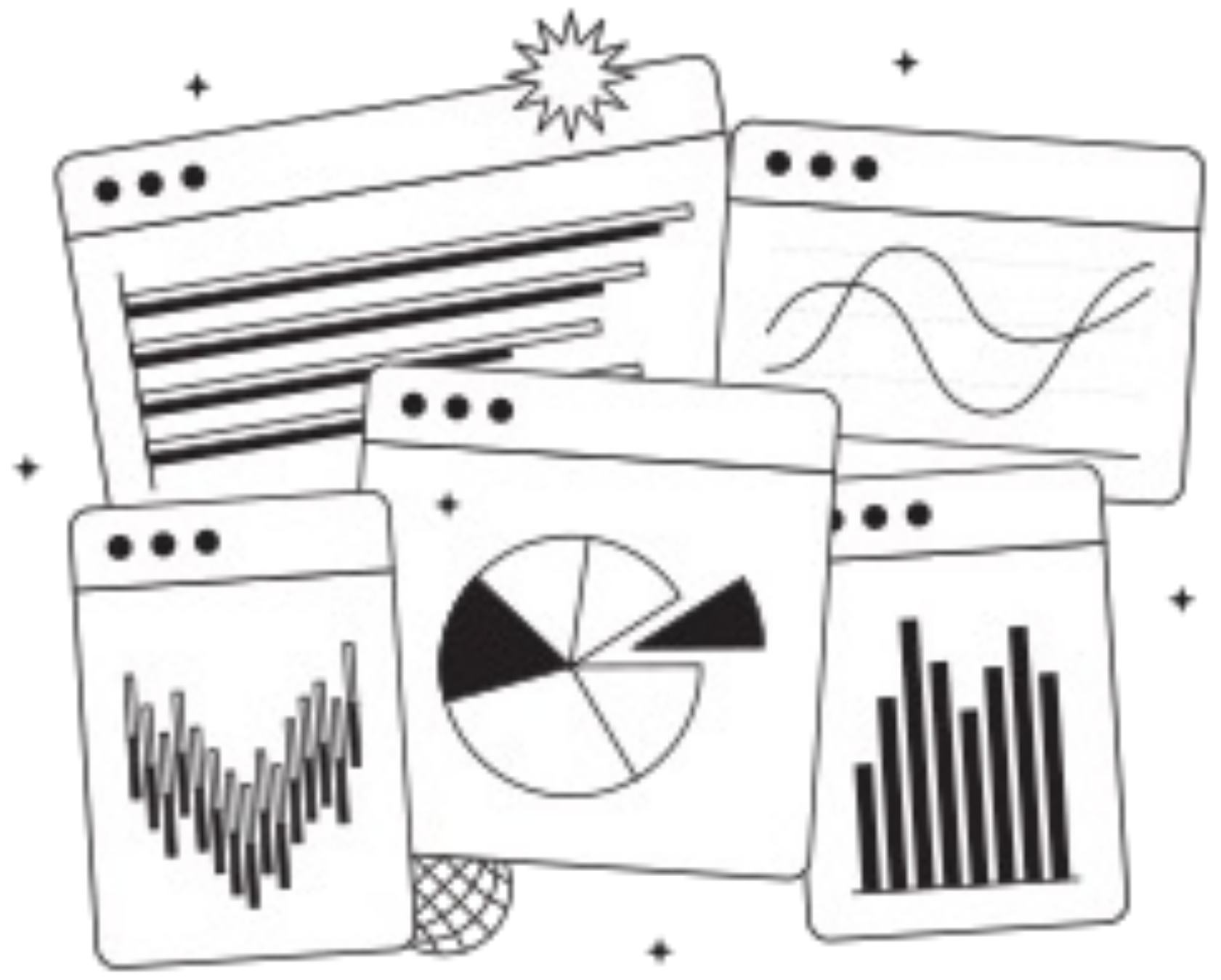

Kimola'nın makine öğrenimi teknolojisi ile migren hastalığı ve tedavi süreçleri hakkında soru soran kişilerin verileri 4 ana kategori altında analiz edildi:

- Tedavi Öncesi
- Tedavi Süreci
- Tedavi Sonrası
- Hastalık Deneyimi

Ana Kategorilerin Dağılımı

Toplanan tüm veriler dört ana kategoride sınıflandırılmıştır. Migren hastası olan veya semptomlarını gözlemleyen kişilerin tedavi öncesi dönemle ilgili verileri %56 oranında bir hacme sahipken, tüm verilerin dörtte birinden fazlası halihazırda hastaların tedavi süreci ile ilgili sorularından oluşmaktadır.

Migrenin hastanın günlük yaşamına yansıyan psikolojik ve fiziksel etkilerine ek olarak sosyal yaşamlarını da kapsayan "Hastalık Deneyimi" kategorisi %13'lük bir paya sahipken hastaların tedavileri hakkında sonuç aldıkları "Tedavi Sonrası" kategorisi en az veriye (%4) sahiptir.

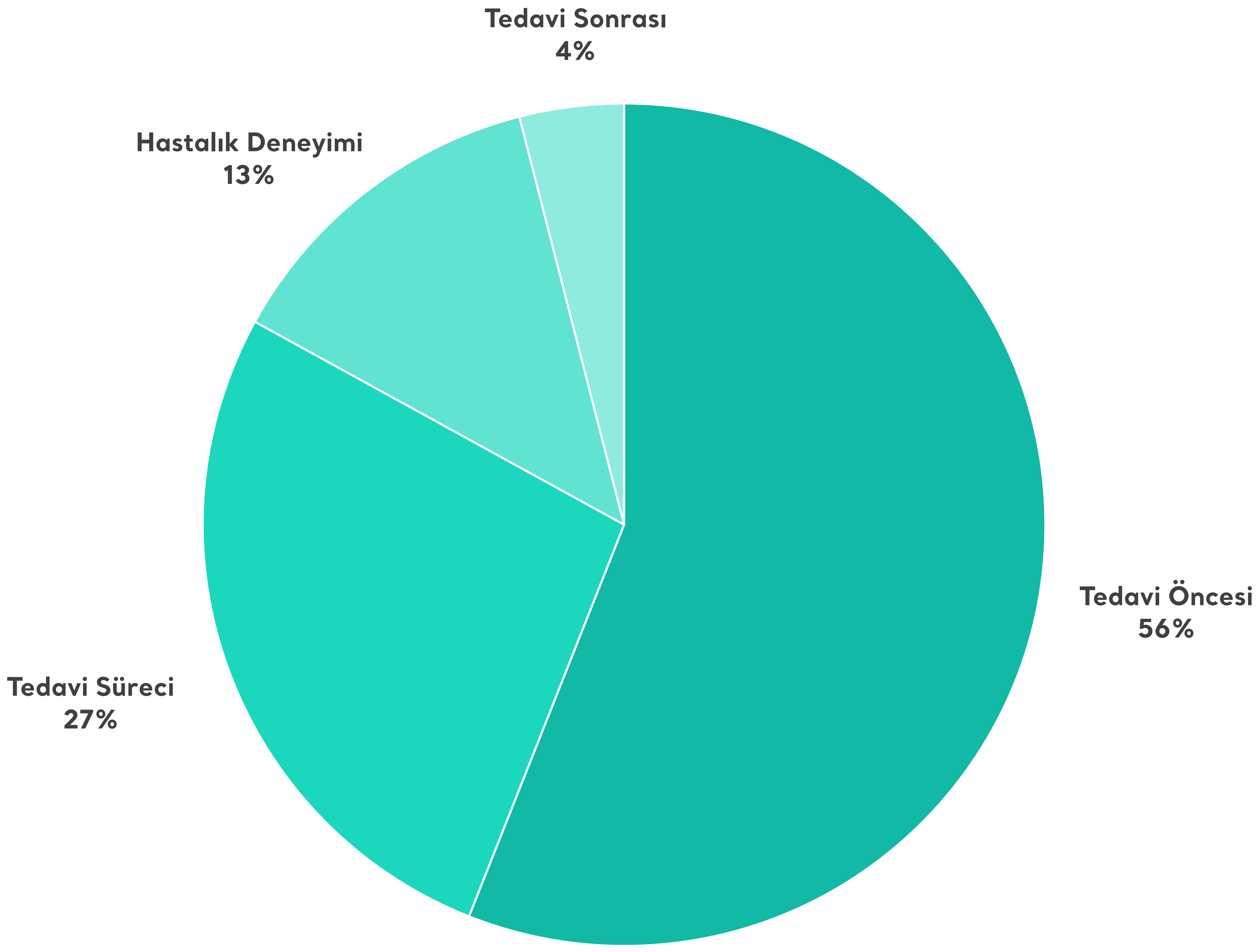

Hasta ve Hasta Yakını Dağılımı

Toplanan tüm veriler dört ana kategoride sınıflandırılmıştır. Migren hastası olan veya semptomlarını gözlemleyen kişilerin tedavi öncesi dönemle ilgili verileri %56 oranında bir hacme sahipken, tüm verilerin dörtte birinden fazlası halihazırda hastaların tedavi süreci ile ilgili sorularından oluşmaktadır.

Migrenin hastanın günlük yaşamına yansıyan psikolojik ve fiziksel etkilerine ek olarak sosyal yaşamlarını da kapsayan "Hastalık Deneyimi" kategorisi %13'lük bir paya sahipken hastaların tedavileri hakkında sonuç aldıkları "Tedavi Sonrası" kategorisi en az veriye (%4) sahiptir.

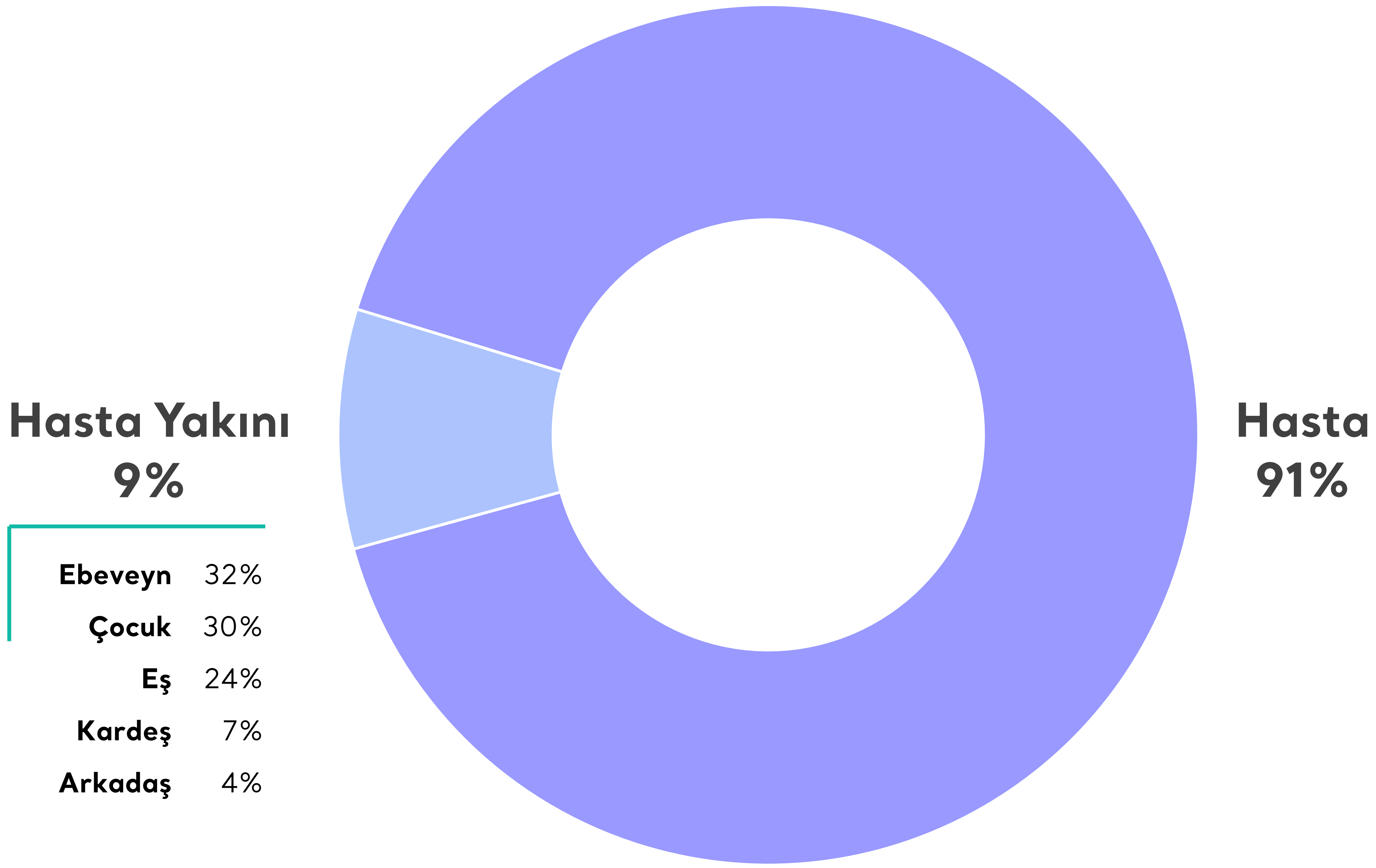

HASTANIN YOLCULUĞU

Kendilerinde veya yakınlarında hastalık semptomlarını fark eden kişiler, hastalık ve tedavi arama sürecine başlayarak en kısa sürede tedavi için en uygun doktora ve ilaca ulaşma sürecini deneyimliyorlar.

1 Semptomlar

Migren hastaları, ışığa ve sese hassasiyet, mide bulantısı ve görme bozuklukları ile birlikte yoğun baş ağrıları yaşarlar. Stres ve belirli yiyecekler gibi tetikleyiciler, günlük aktiviteleri ve yaşam kalitesini etkilemeye başlar ve ağrının sıklığı ile şiddeti artar.

2 Araştırma

Potansiyel hastalar hem arama motorları hem de sosyal medyada kendilerinde gördükleri semptomları paylaşarak güvenilir doktorlara ulaşmak ve semptomların migren ile ilgili olup olmadığı konusunda araştırma yaparlar.

3 Teşhis

Profesyonel desteğe ihtiyacının farkına varan hasta, ilk aşamada hekimine danışmaya karar verir. Doktor, migren teşhisi koyabilmek adına hastanın tıbbi öyküsünü ve semptomlarını göz önünde bulundurarak kapsamlı bir muayene yapar. Gerekli MR ve kan testlerine girerek teşhis prosedürüne devam eden hastayı doktor, test sonuçları doğrultusunda ilaç veya diğer tedavi yöntemlerine yönlendirir.

4 Tedavi

Ardından doktorun yönlendirmesiyle tedaviye başlayan hasta, tedavi sürecinde yaşadığı atakların sıklığı, ilacın etkinlik süresini ve vücudunda oluşabilecek olası yan etkileri takip etmeye başlar. Bu süreçte hasta doktoru ile iletişimini sürdürerek hastalık ile ilgili gelişmeler konusunda bilgilendirir ve tedavinin devamı konusunda doktorundan bilgi alır.

5 Adaptasyon

Teşhisin ardından tedavi süreciyle devam eden hasta, kendi yaşamında deneme yanılma yöntemleriyle belirli gıdalar, çevresel faktörler veya hormonal değişiklikler gibi tetikleyicileri tanımlayarak günlük rutinlerinde, diyetlerinde, egzersiz alışkanlıklarında ve uyku düzenlerinde karşılık gelen değişiklikleri yapar.

6 Yan Etkiler

İlaçların yan etkileri hastaların tedavi süreci ile ilgili en büyük endişesini oluşturmaktadır. Gözlemledikleri veya karşılaşabilecekleri yan etkiler, ilaç kullanımının beyinlerine ya da vücutlarına kalıcı zarar verme olasılığı gibi konular hakkında hastalarda endişeye neden olmaktadır.

7 İyileşme

Yaşam tarzı ayarlamaları, önleyici ilaçlar ve sağlık uzmanlarıyla devam eden iletişimin bir kombinasyonu sayesinde, hasta migrenlerin sıklığında ve şiddetinde önemli bir azalma elde eder, sonuçta normallik duygusunu yeniden kazanır ve genel refahını iyileştirir.

Tedavi Öncesi

Tedavi Öncesi

Ana Kategori

1,379 veri

Migren hastaları veya belirtileri gözlemleyen kişilerin %71'i, tedavi yöntemleri, ilaçların olası yan etkileri ve hastalık belirtileriyle ilgili araştırma yapmaktadır. Her 10 kişiden neredeyse 7'si, gözlemledikleri belirtilerin migrenle ilişkili olabileceğini düşünerek doktorlara danışmaktadır.

Muayene olan hastalar arasında, tedaviye devam etmeden önce MR ve diğer test sonuçlarını kendi doktorları yerine internet üzerindeki doktorlara danışma eğilimi yaygın olarak görülmektedir. Ayrıca, konulan teşhislerin doktorlar tarafından teyit edilmesini isteyenler, Tanı kategorisindeki verilerin üçte birini oluşturmaktadır.

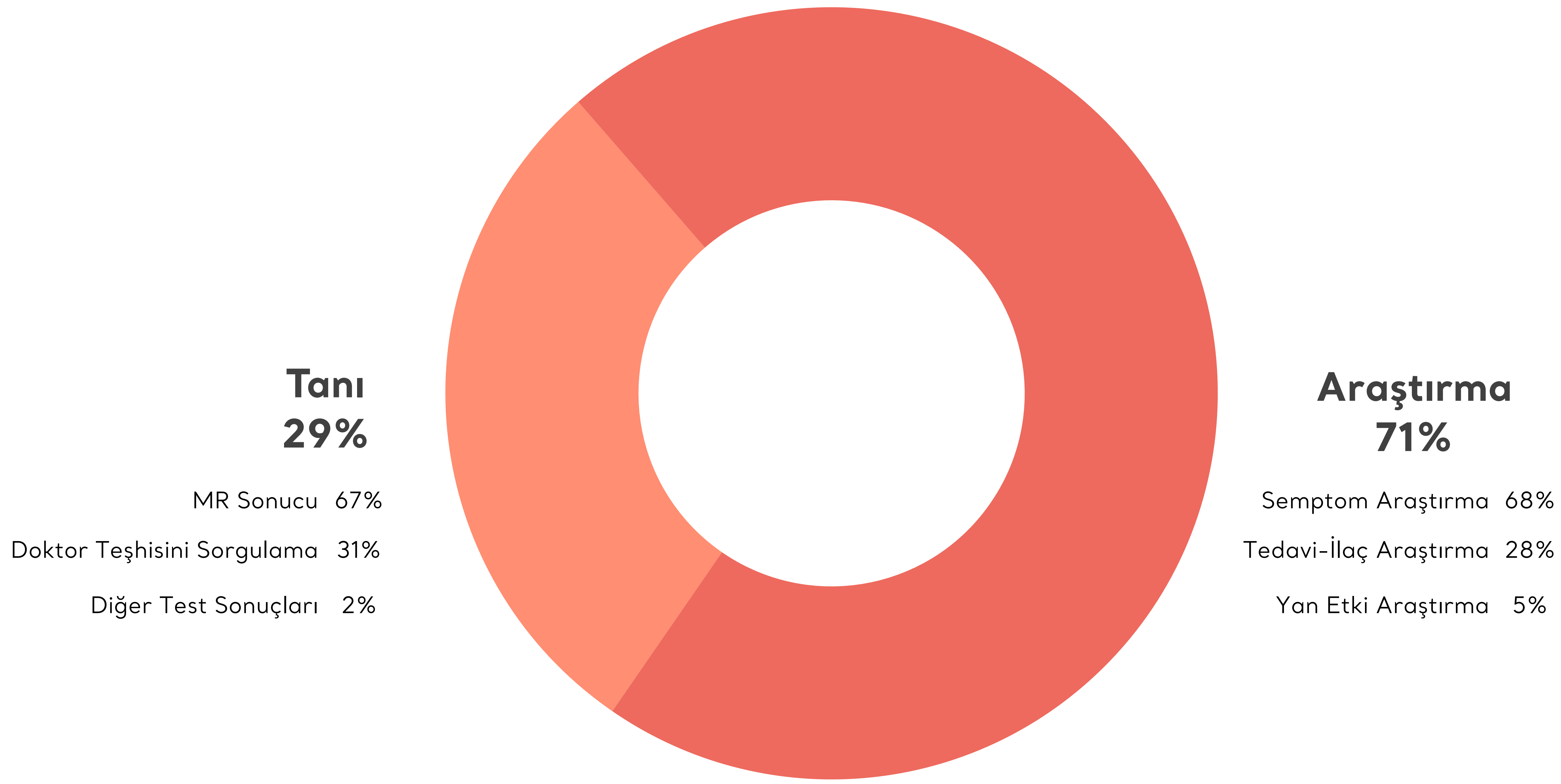

%1'den az hacme sahip alt kategoriler yukarıdaki tabloda gösterilmemiştir.

Araştırma

Alt Kategori

979 veri

Potansiyel hastalar, fiziksel olarak muayene olmadan önce, ilk etapta internet üzerinde araştırma yapıyorlar.

Bu süreçte, kendilerinde gözlemledikleri semptomları araştıranlar analizi yapılan verinin üçte ikisini kapsarken, ne yapacağı konusunda belirsizlik yaşayan ve olası yan etkilerden endişe duyan hastalar ise verinin geri kalan %32'sini oluşturmaktadır.

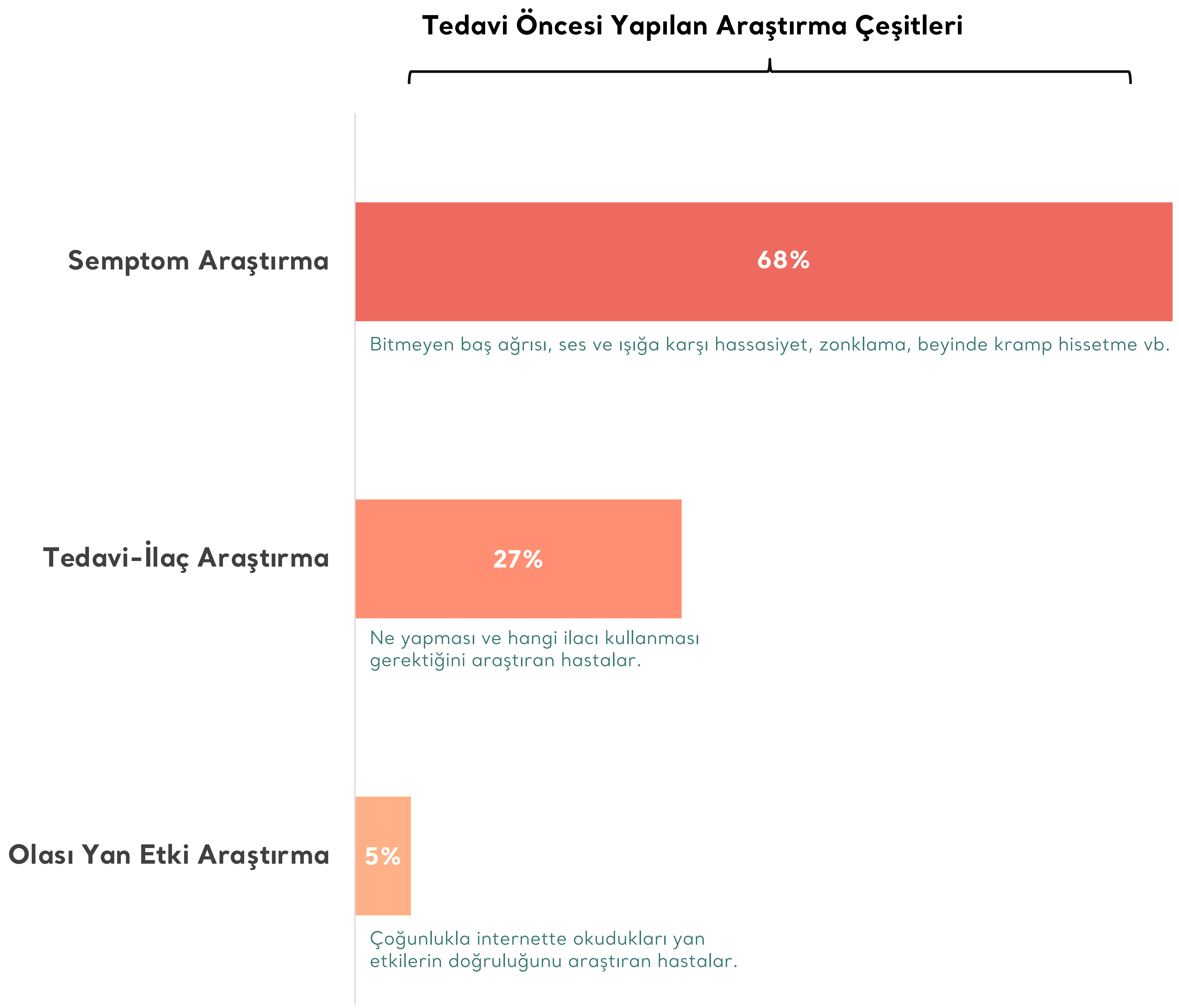

kimola

içgörü

Klasik baş ağrısı ile karıştırılıyor.

Araştırma sonuçlarına göre, migren hastaları klasik baş ağrısı ile migren arasında karışıklık yaşıyor. Hastalar, **migren mi yoksa kısa sürede geçen bir ağrı** mı yaşadıklarını anlamakta zorlanıyor. Bu belirsizlik, migren belirtilerini tanımlamada ve baş ağrısı türleri arasındaki farkı anlamada zorluk çekmelerine neden olabilir. Hastalar kendi başlarına çözüm aramadan önce doğru tanı ve tedavi için uzmanlara başvurmadan önce **daha fazla bilgiye ihtiyaç duyuyorlar**.

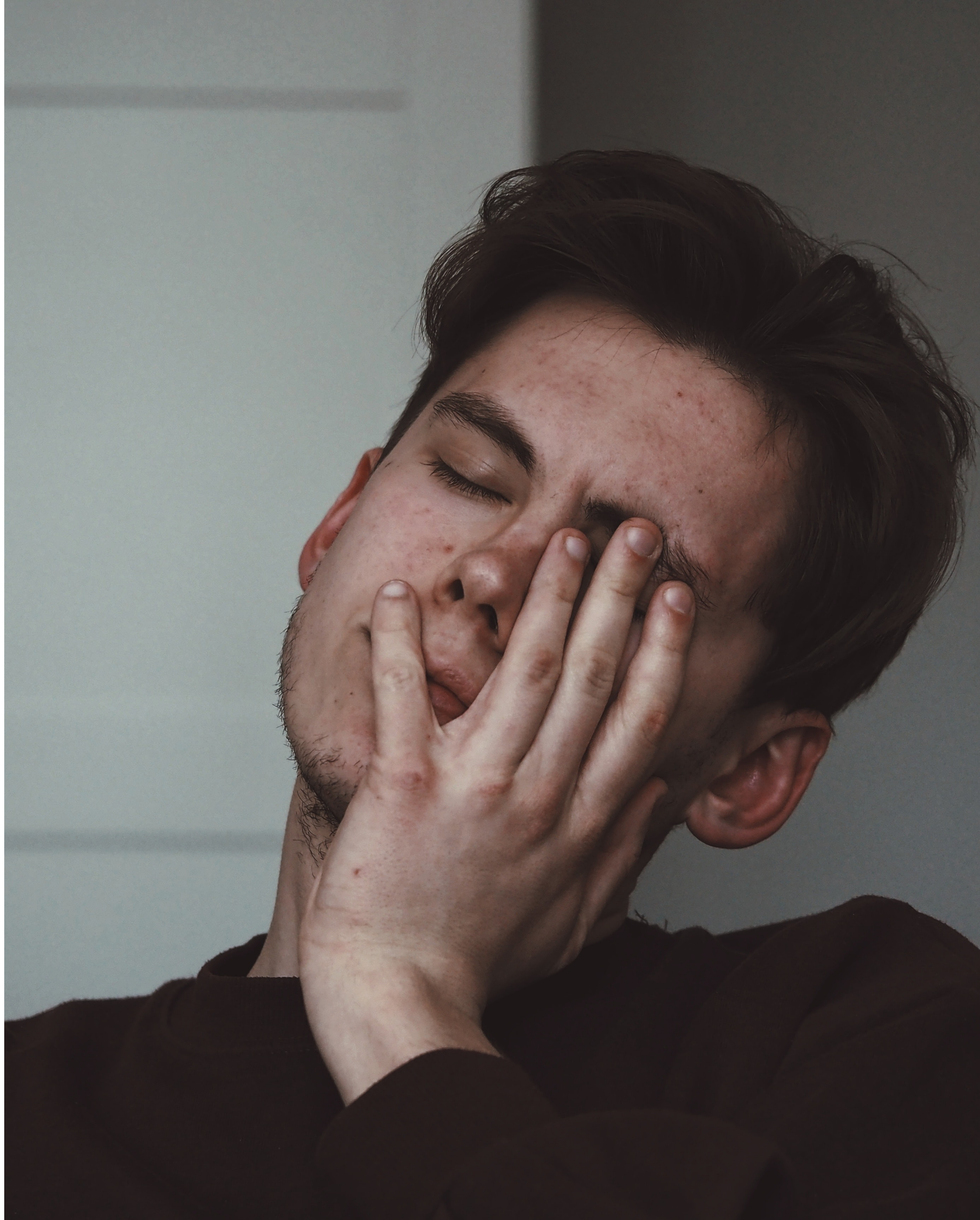

kimola

içgörü

Hastalık mı, semptom mu?

Migrenin başlı başına bir hastalık olup olmadığı potansiyel hastaların kafa karışıklığı yaşamasına neden oluyor. Zaman geçtikçe hastalar, migrenin **başka büyük bir problemin işareti/belirtisi** olmasından endişe duyuyorlar.

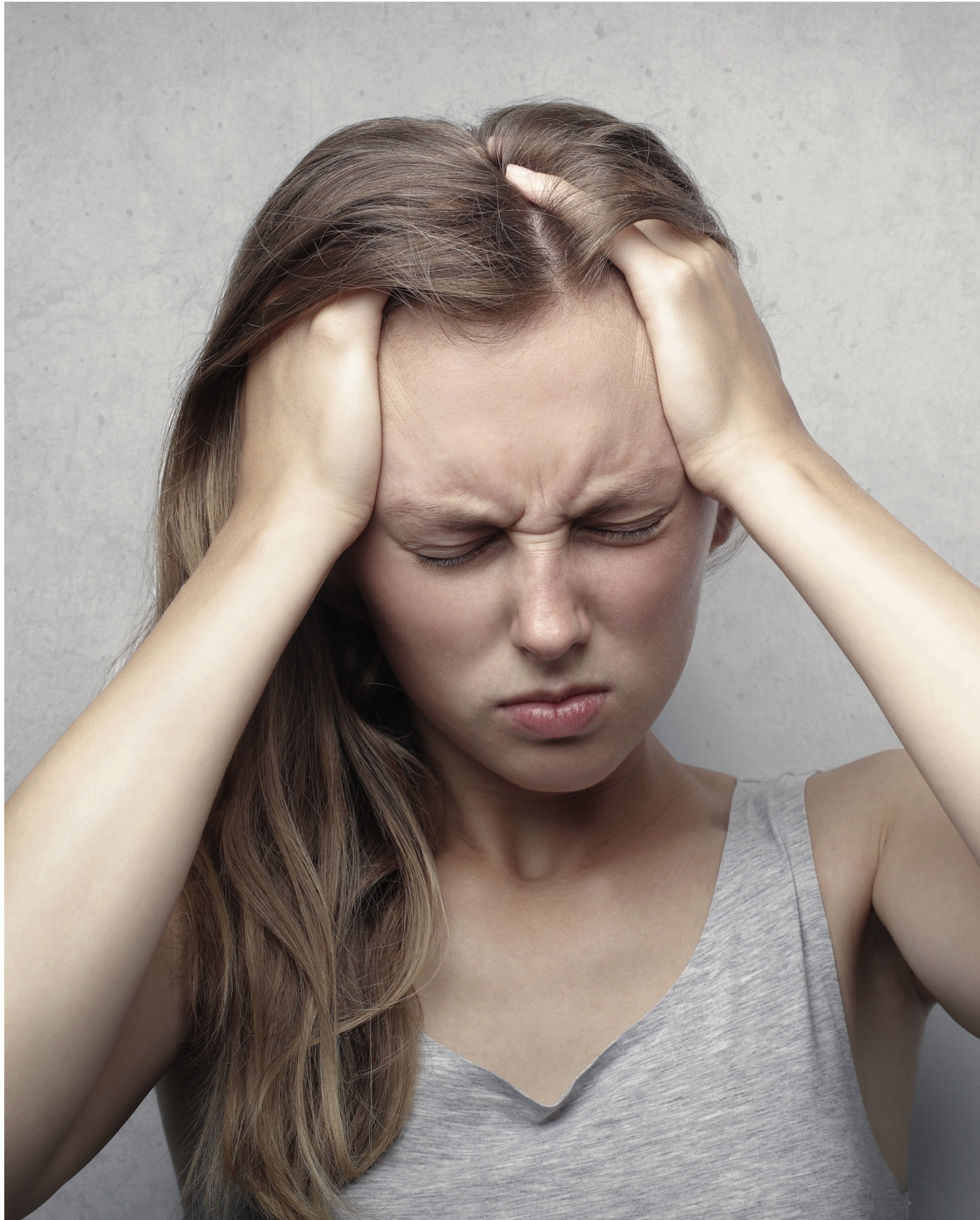

kimola

içgörü

“Erkekler de migren olur mu?”

Migren ile ilgili ilk kez araştırma yapan kişilerde, migrenin **cinsiyete göre değişkenlik gösterdiği ve/veya kadınlarda daha sık görülen bir hastalık** olma ihtimaline yönelik düşüncelerin olduğu görülüyor. Migren konusunda temel bilgilerde eksiklik olan hastalar, önerilen **tedavi yöntemlerine karşı da mesafeli yaklaşıyor** olabilirler.

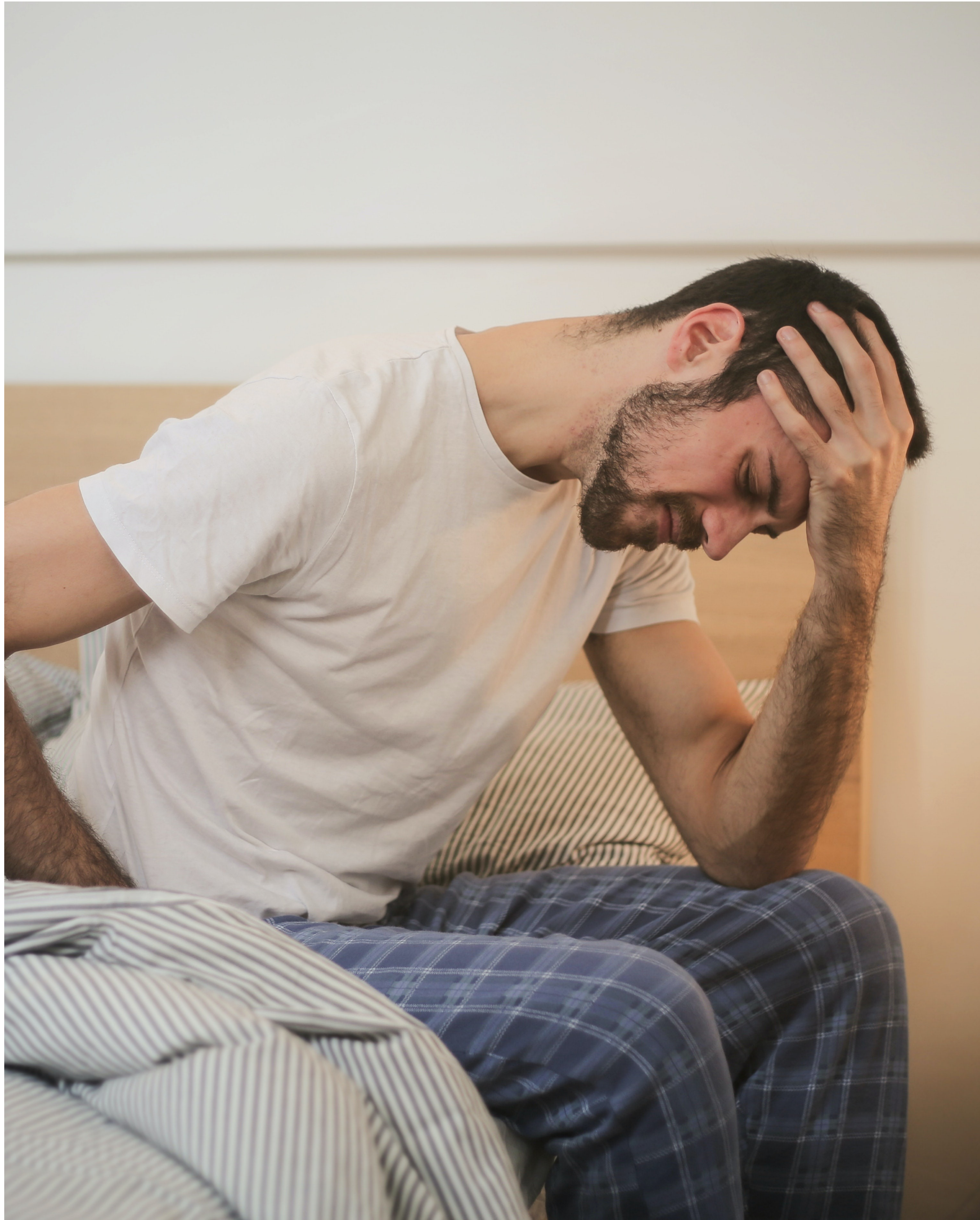

içgörü

Migrenin kalıtsal olma ihtimali.

Aile hastalık öyküsünde migren bulunan hastalar, kalıtsal olarak kendilerininde migren hastası olma ihtimallerini araştırıyorlar. Bundan endişe duyan hastalar arasında migren kadar yoğun olmayan bir başağrısının da migrenin işaretçisi olabileceği düşüncesi yaygın olarak gözlemleniyor.

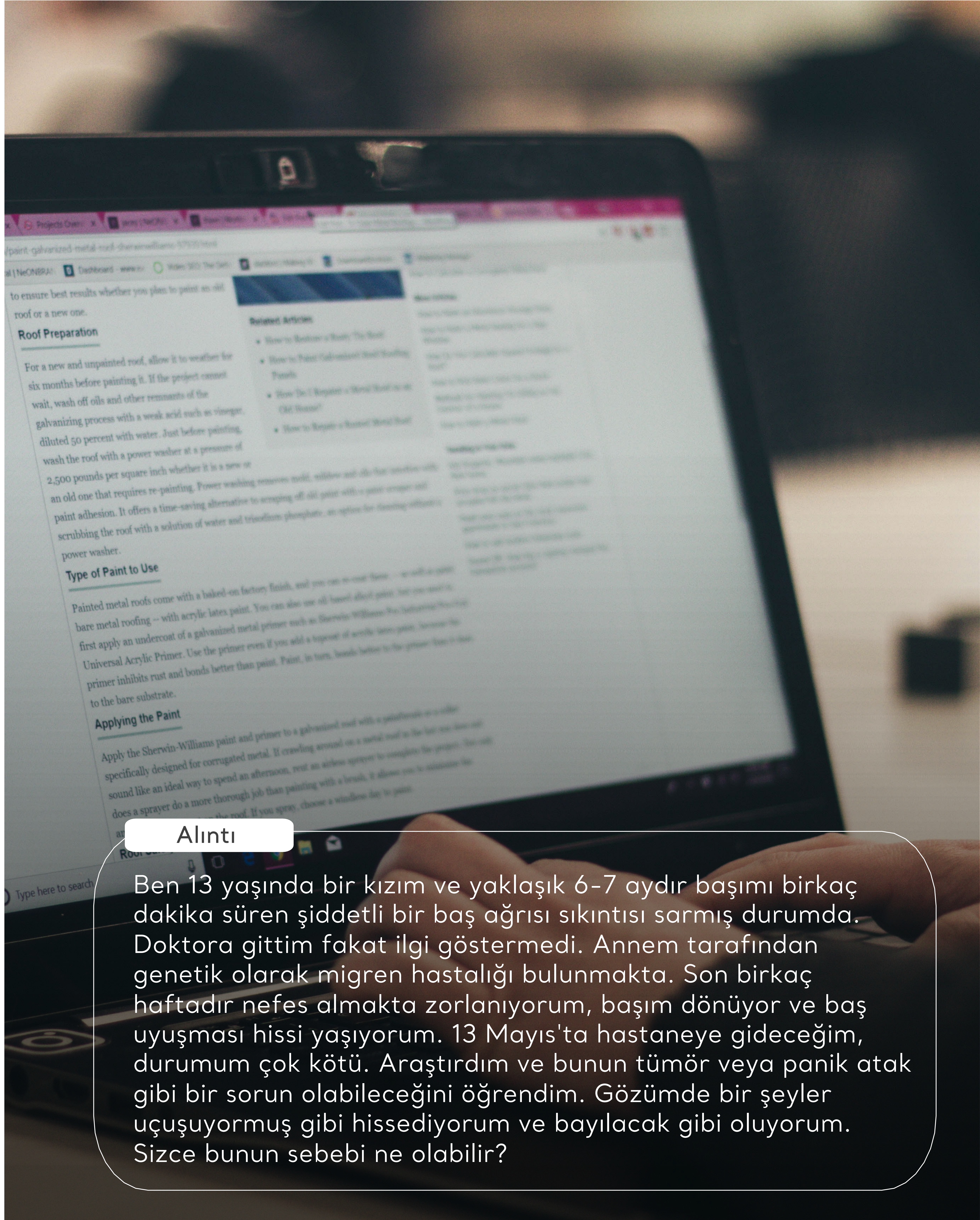

Alıntı

Ben 13 yaşında bir kızım ve yaklaşık 6-7 aydır başımı birkaç dakika süren şiddetli bir baş ağrısı sıkıntısı sarmış durumda. Doktora gittim fakat ilgi göstermedi. Annem tarafından genetik olarak migren hastalığı bulunmakta. Son birkaç haftadır nefes almakta zorlanıyorum, başım dönüyor ve baş uyuşması hissi yaşıyorum. 13 Mayıs'ta hastaneye gideceğim, durumum çok kötü. Araştırdım ve bunun tümör veya panik atak gibi bir sorun olabileceğini öğrendim. Gözümde bir şeyler uçuşuyormuş gibi hissediyorum ve bayılacak gibi oluyorum. Sizce bunun sebebi ne olabilir?

kimolq

içgörü

Yan etkilerinin farkında olarak kullanmak istiyorlar.

Migren hastalığının **uzun vadede bedende yaratacağı etkiler** ve migren ilaçları kullananlar da **sıklıkla gözlemlenen yan etkiler** konusunda araştırma yapan hastalar, tedaviye başlamaya bu süreçte karar veriyorlar.

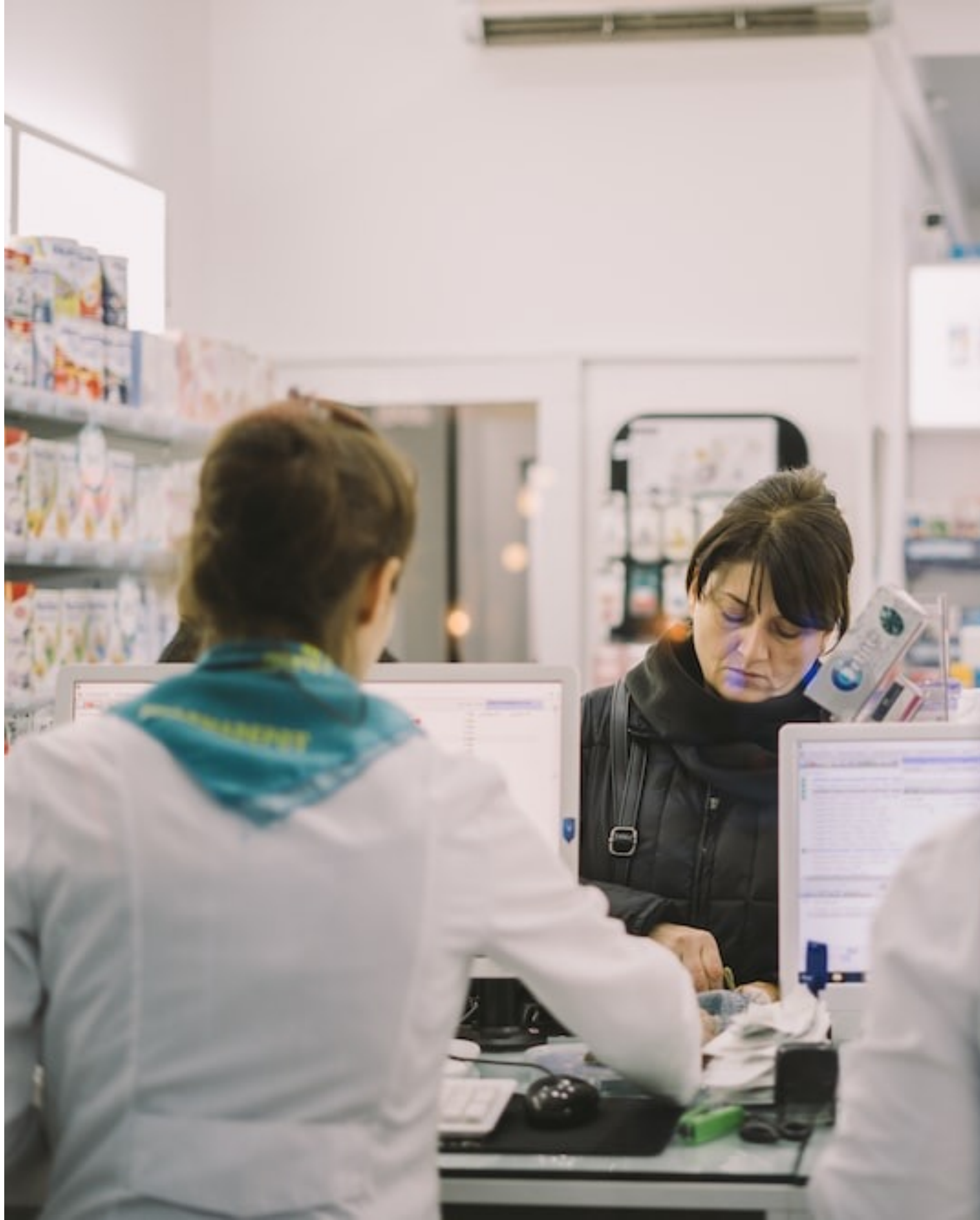

kimolq

içgörü

Beyin hasarı ihtimali ile ilgili söylentiler bariyer oluşturuyor.

Migren tanısı konmuş fakat henüz ilaç tedavisine başlamamış ve araştırma yapan hastalar, internette karşılaştıkları ve migren ilaçlarının **beyinde önemli ölçüde hasar yarattığına dair iddialar** ilaç kullanmalarına karşın önemli bir bariyer oluşturuyor.

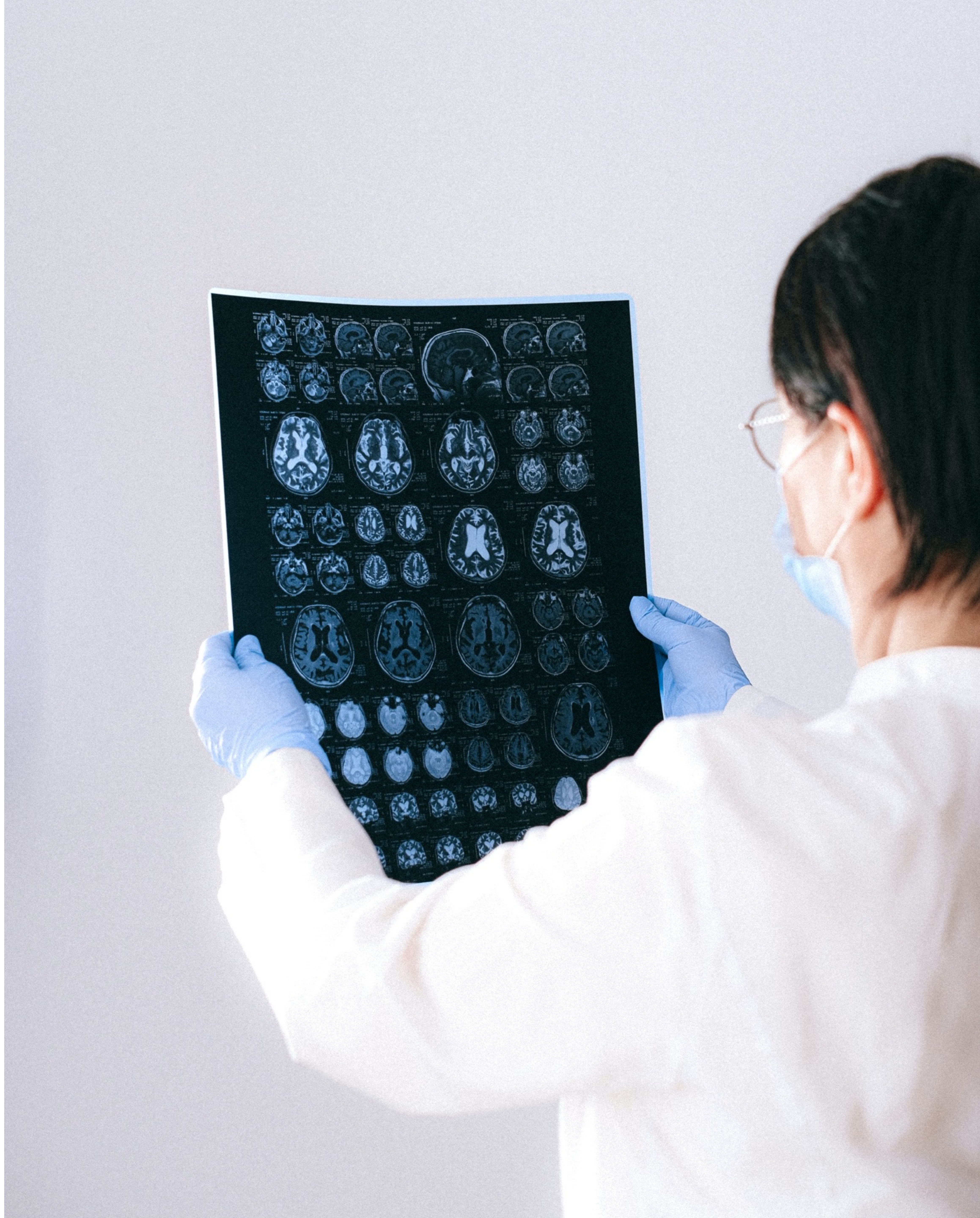

içgörü

Kilo alma önemli bir bariyer.

Migren hastalığının yanında kilo problemleriyle de mücadele eden hastalar, ilaçları kullanmaya başlamadan önce kilo aldırma ihtimallerini araştırıyorlar. Hastalar arasında migren ilaçlarının **ağrıyı geçirse de, kilo alımına neden olduğu algısı** sıklıkla görülüyor.

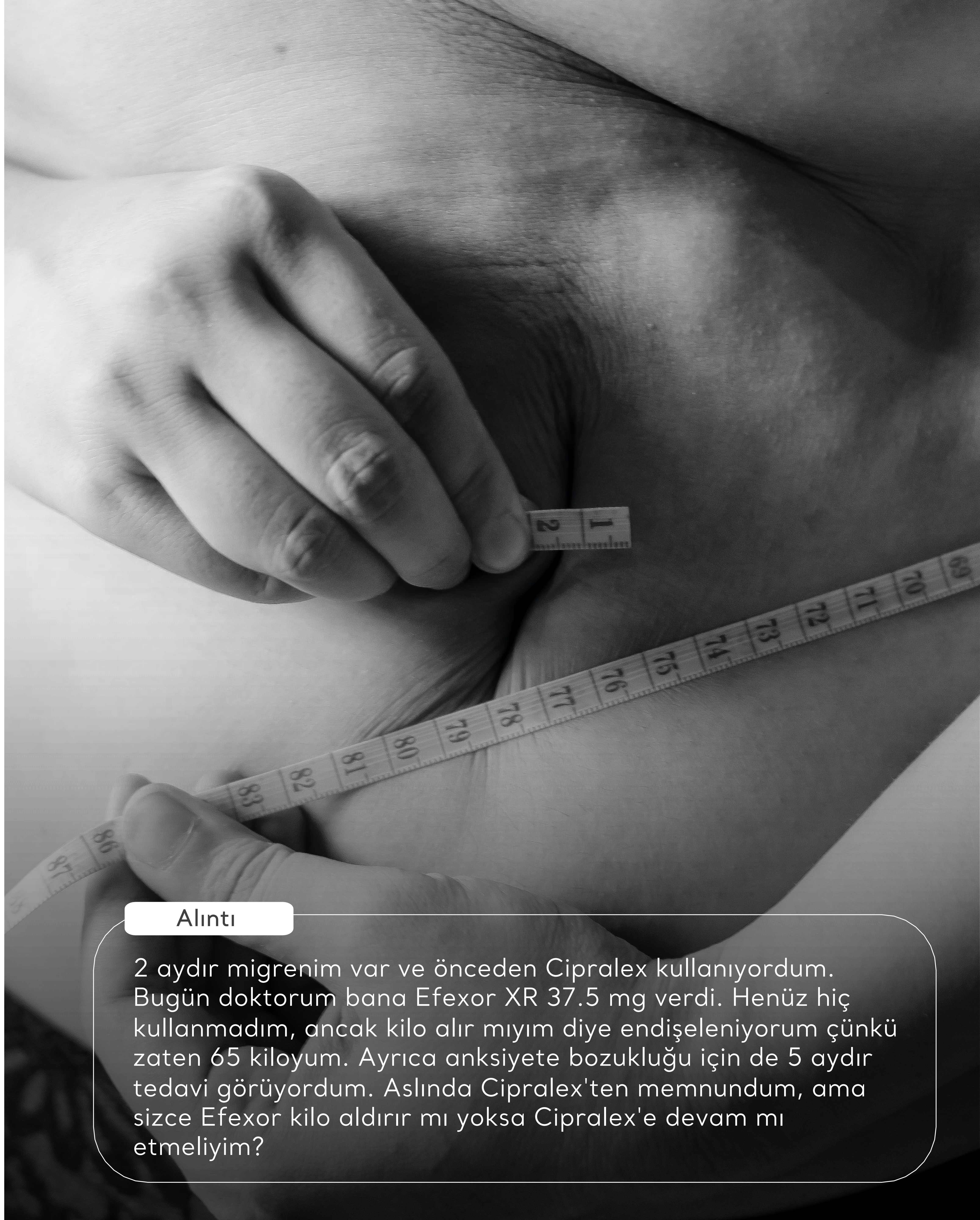

Alıntı

2 aydır migrenim var ve önceden Cipralex kullanıyordum. Bugün doktorum bana Efexor XR 37.5 mg verdi. Henüz hiç kullanmadım, ancak kilo alır mıyım diye endişeleniyorum çünkü zaten 65 kiloyum. Ayrıca anksiyete bozukluğu için de 5 aydır tedavi görüyordum. Aslında Cipralex'ten memnundum, ama sizce Efexor kilo aldırır mı yoksa Cipralex'e devam mı etmeliyim?

kimola

içgörü

“Doktora gitmeye korkuyorum.”

Migrenin beyindeki sinirler ve kan damarlarında oluşan değişiklikler sonucu ortaya çıkan bir ağrı tipi olması, potansiyel hastaların beyinleri ile ilgili ciddi bir hastalıkla yüzleşmekten korkmalarına neden oluyor olabilir.

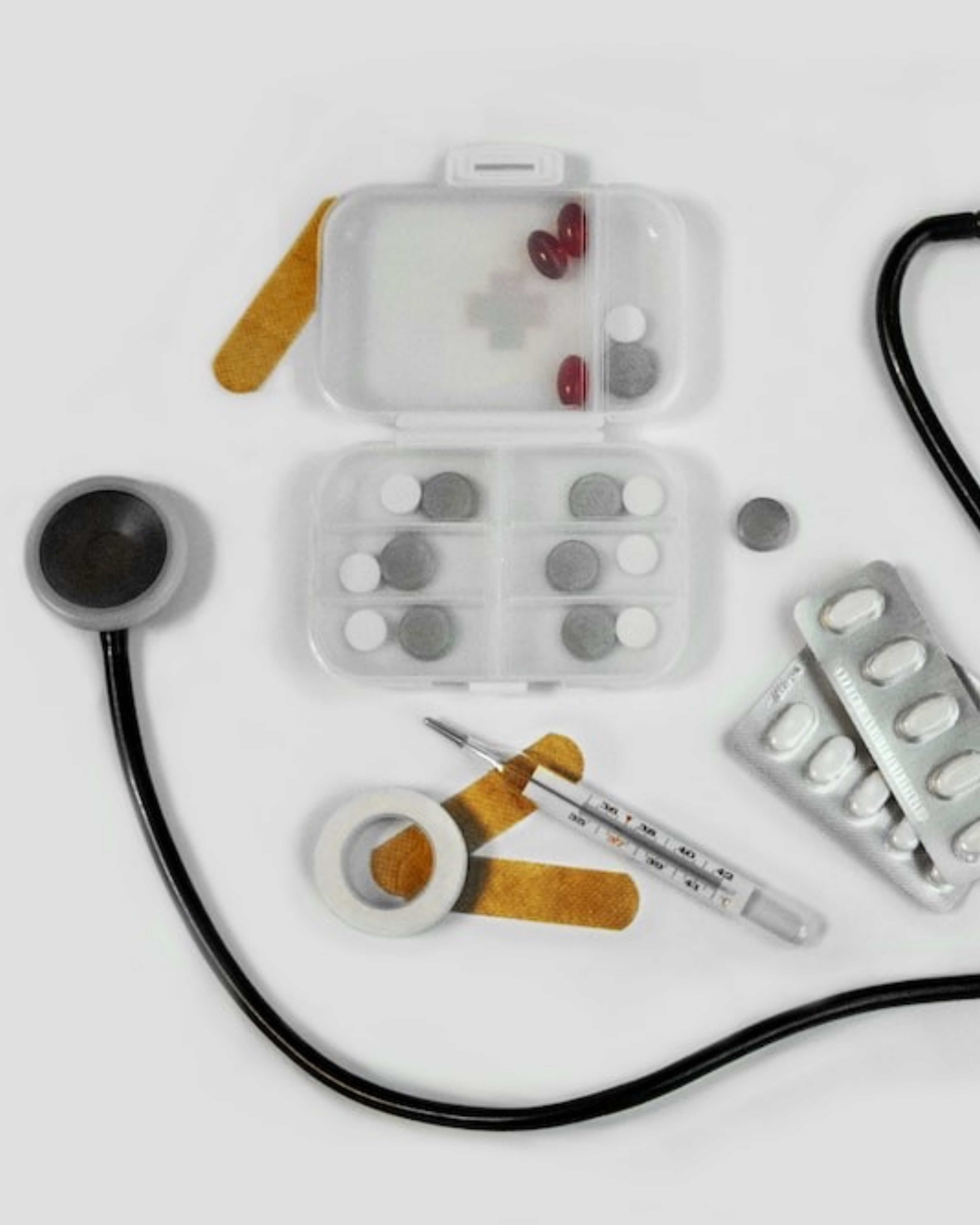

içgörü

Dayanamamayacak noktaya gelmeyi bekliyorlar.

Doktora gitmek ve doktorun istediği incelemeler/testler ile uğraşmak potansiyel hastalar için bir bariyer oluştururken, hastalar gerek ilaç tedavisine başlamak gerekse doktor muayenesi için **hissettikleri acı ve ağrının dayanılamayacak noktaya gelmesini bekliyorlar**.

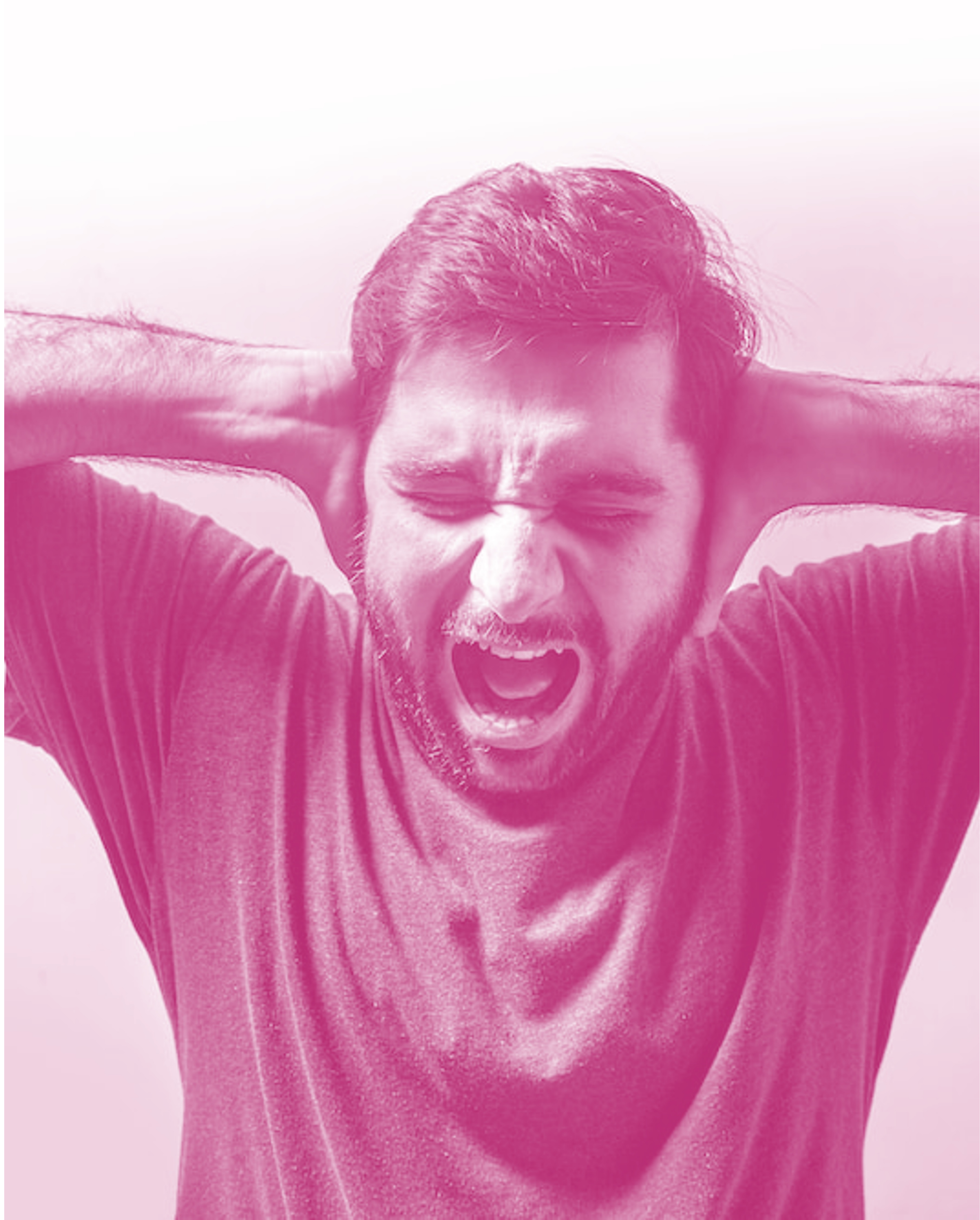

kimola

içgörü

Muayene için ikna edilmeleri gerekiyor.

Migren semptomlarını gözlemleyen hastaların çoğunluğu, bir doktora başvurup fiziksel bir muayene yapılması gerektiği konusunda ikna edilmek istiyor. Hastalar, doğru teşhisin ve etkili bir tedavinin sağlanabilmesi için **doktorlarına güven duymak istiyorlar**. Muayene olmak, semptomların migrene özgü olduğunu doğrulayarak hastaların endişelerini gidermeye yardımcı oluyor.

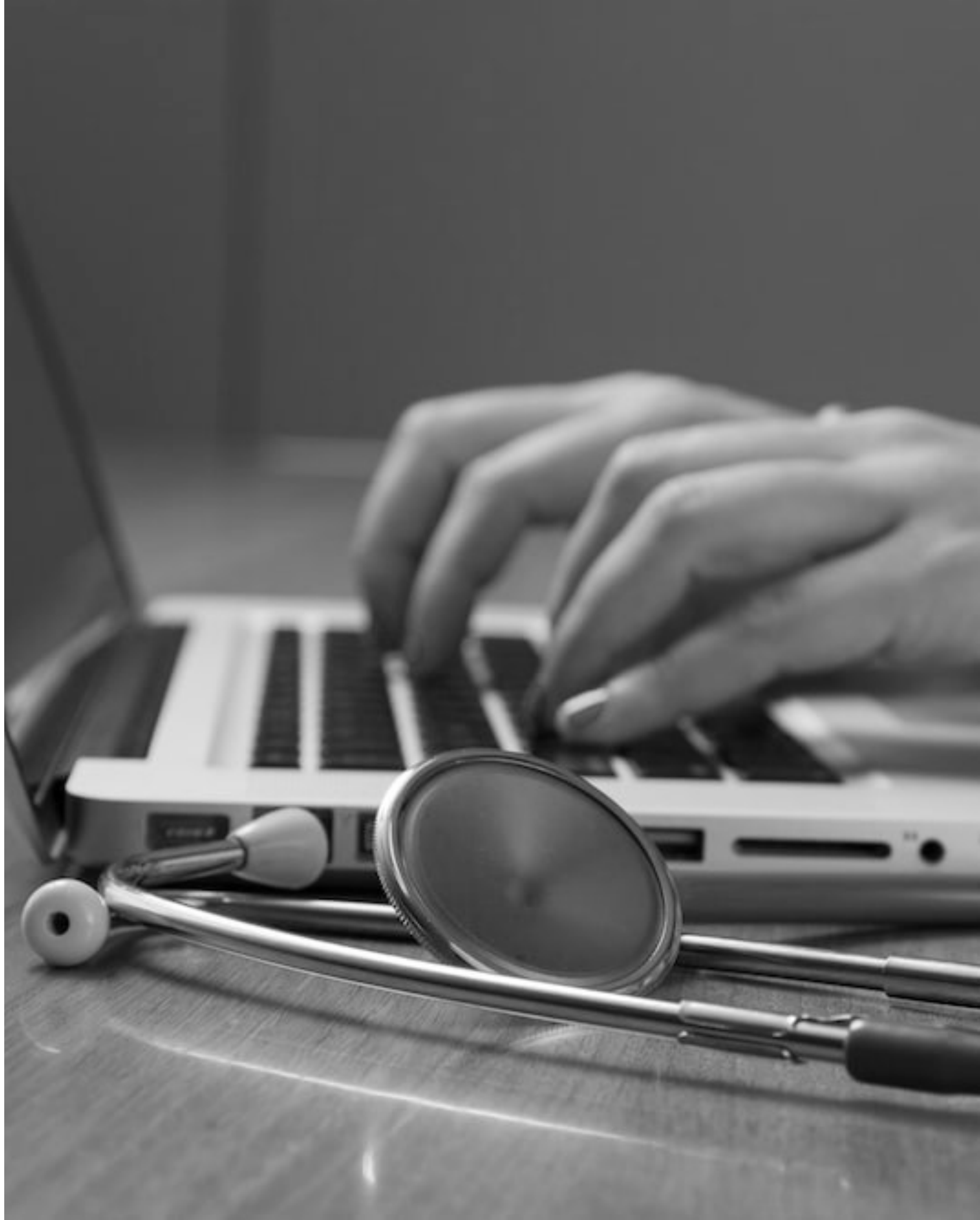

kimola

içgörü

Auralı migren ile göz bozukluğu karıştırılıyor.

Bulanık görme, siyah lekeler görme, ışık hassasiyeti gibi hem **göz numarasının yükselmesinin de belirtisi olabilecek durumların** migreni mi yoksa göz bozukluğunu mu işaret etmesi potansiyel hastalar tarafından araştırılıyor.

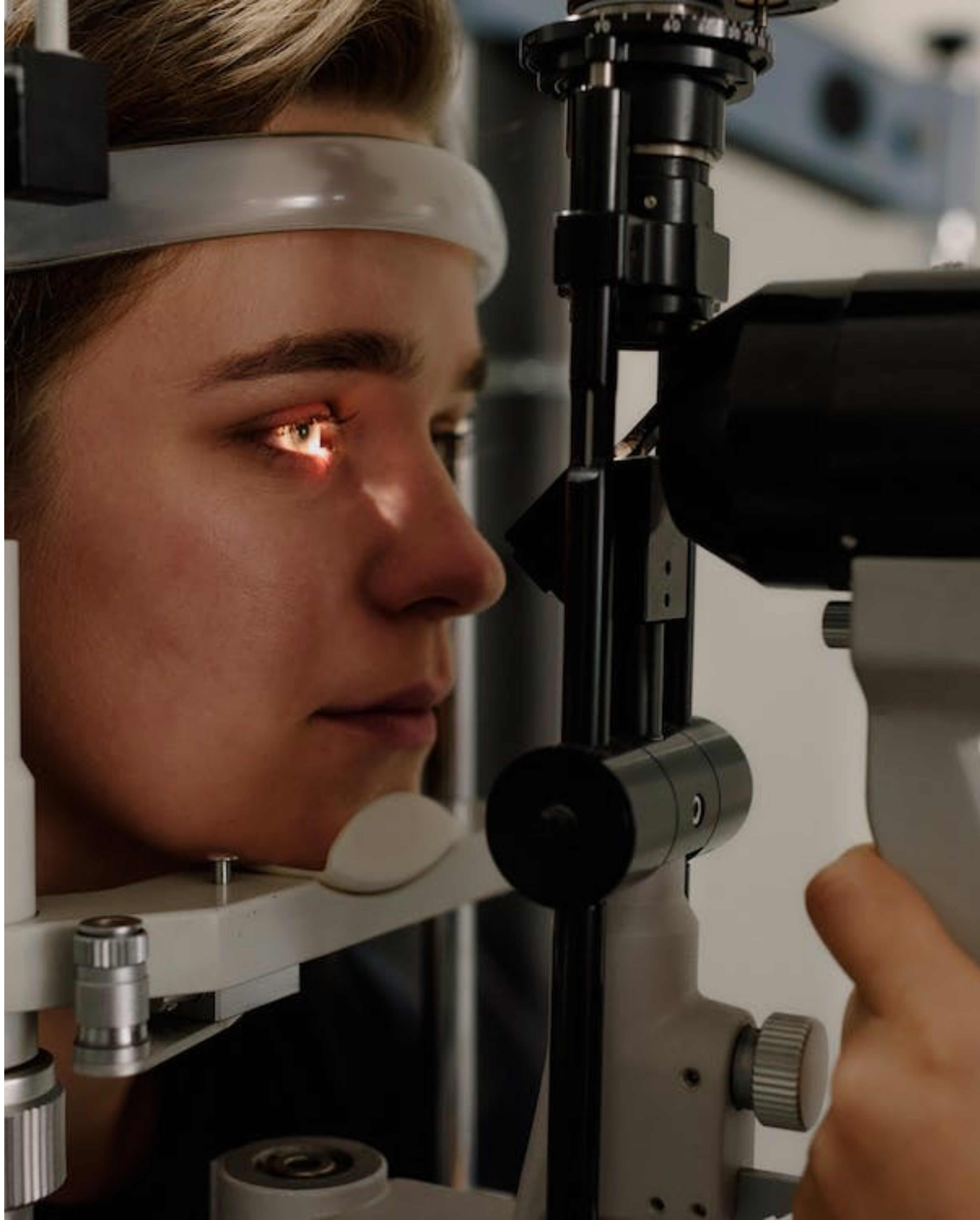

Tanı

Alt Kategori

400 veri

Potansiyel migren hastalarının üçte ikisi, internet üzerinden danıştıkları doktorlardan MR sonuçlarını incelemelerini talep ediyorlar.

Muayene oldukları doktora gitmeden önceden başka bir doktordan da fikir almak isteyen hastaların yanı sıra, muayene oldukları doktorların "migren teşhisinin" doğruluğunu sorgulayan hastalar da %31'lik bir orana sahiptir.

Tanı Alt Kategoriler

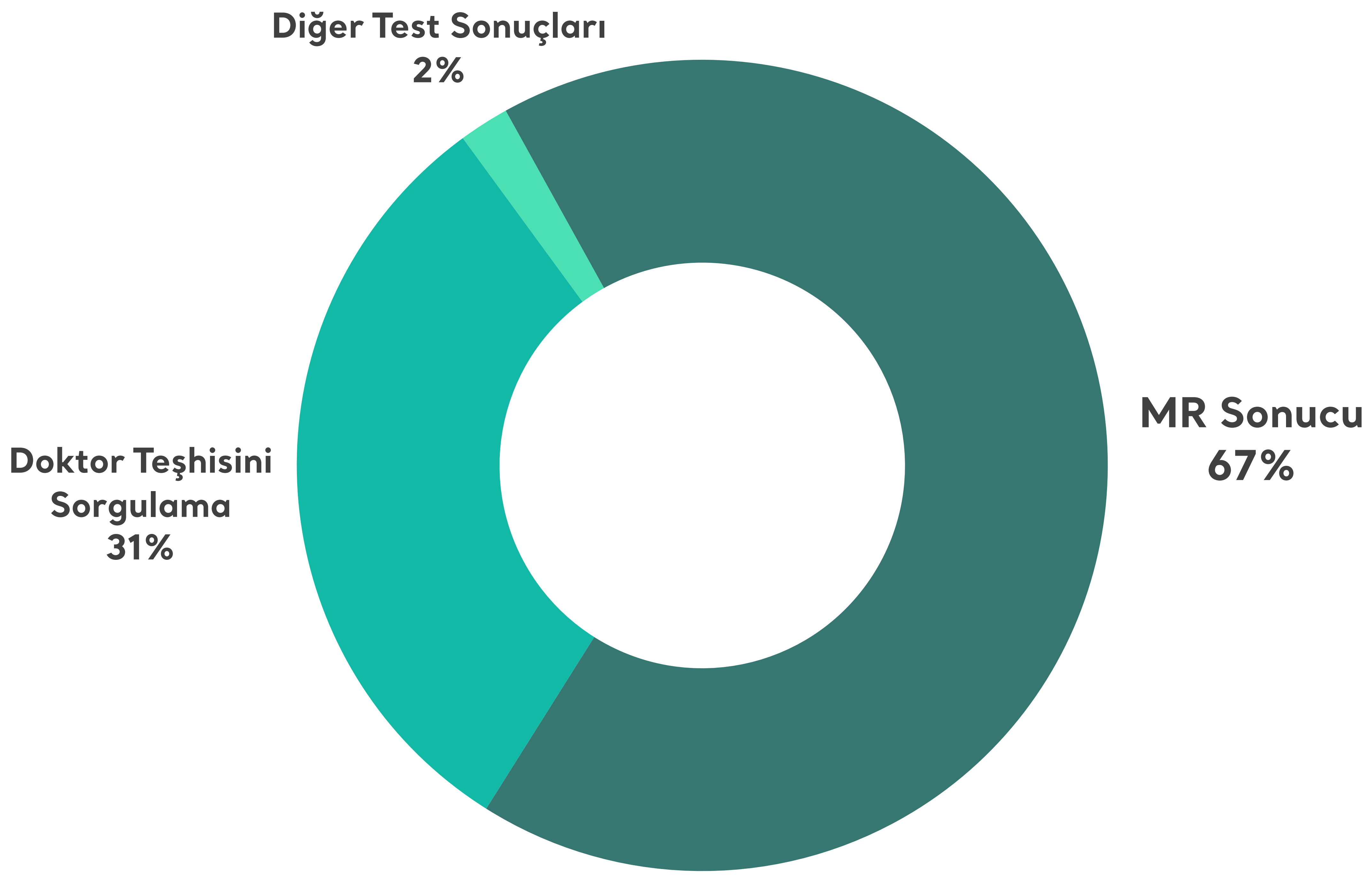

kimola

içgörü

İnternetten alınan test sonuçlarının teyit edilmesi.

Hastaların e-Devlet ve hastanelerin internet siteleri aracılığıyla erişebildikleri MR ve migrenle ilişkili diğer test sonuçlarını, muayene oldukları doktora göstermeden önce internet üzerinden başka bir doktora sorma eğiliminde oldukları görülüyor. Bu durum, hastaların test sonuçlarının yorumlanması için **uzun bir bekleme süresini tercih etmemelerinden kaynaklanıyor** olabilir.

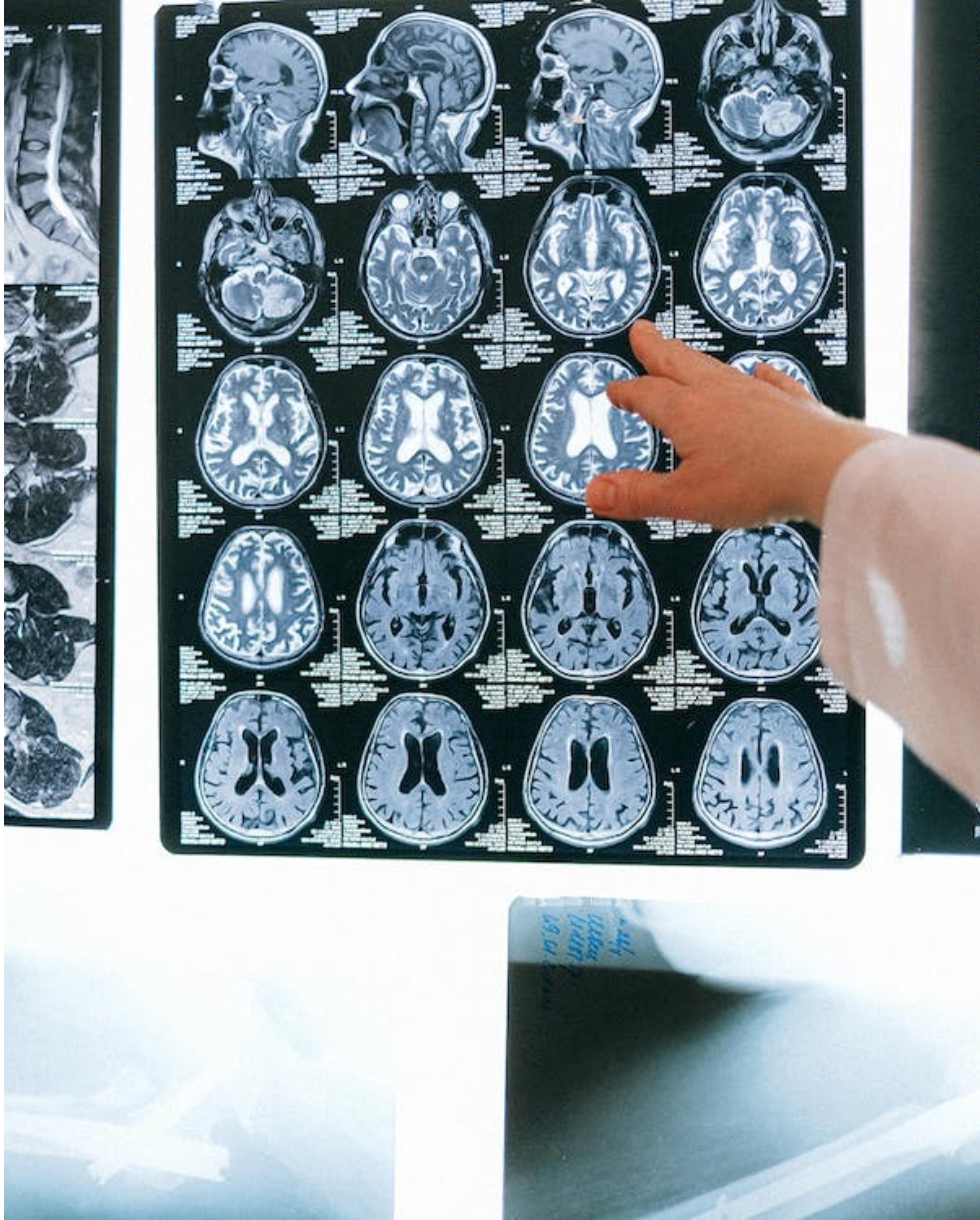

kimolq

içgörü

“Doktorumun teşhisi doğru mu?”

Büyük bir çoğunluk için internetin doğru bilgiye erişim ile ilişkilendirildiğini söylemek mümkün. Bu doğrultuda, doktoru tarafından migren teşhisi konan hastaların, **doktorları tarafından teşhis konulsa bile** internette semptom ve tedavi yöntemleri ile ilgili araştırma yapmaya devam ettikleri görülüyor.

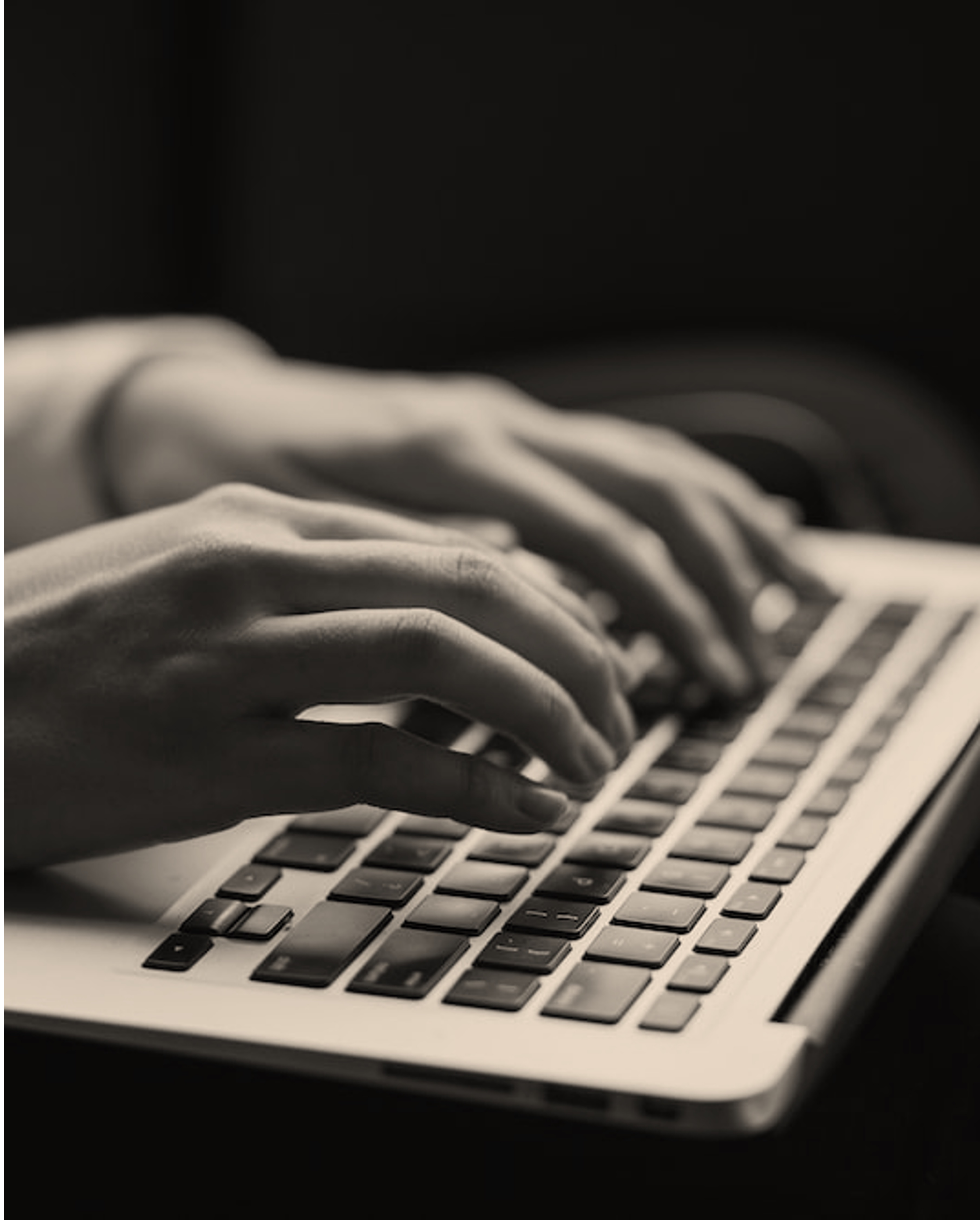

kimolq

içgörü

Migren teşhisinin bir geçiştirme olduğu düşünülebiliyor.

Beklenen fazla migren semptomu yaşayan bazı hastalar, deneyimledikleri durumun migrendense "daha sert başka bir hastalık" olabileceğinden fakat doktorun migren teşhisi koyarak geçiştirdiğinden şüpheleniyorlar. Migren hastalarının **migreni genellikle hafife alındığını veya yanlış teşhis edildiği** düşünmelerinin, doktorlara olan güvenlerini sarsmakta ve tedavi sürecine olan inançlarını etkilemekte olduğunu söylemek mümkün.

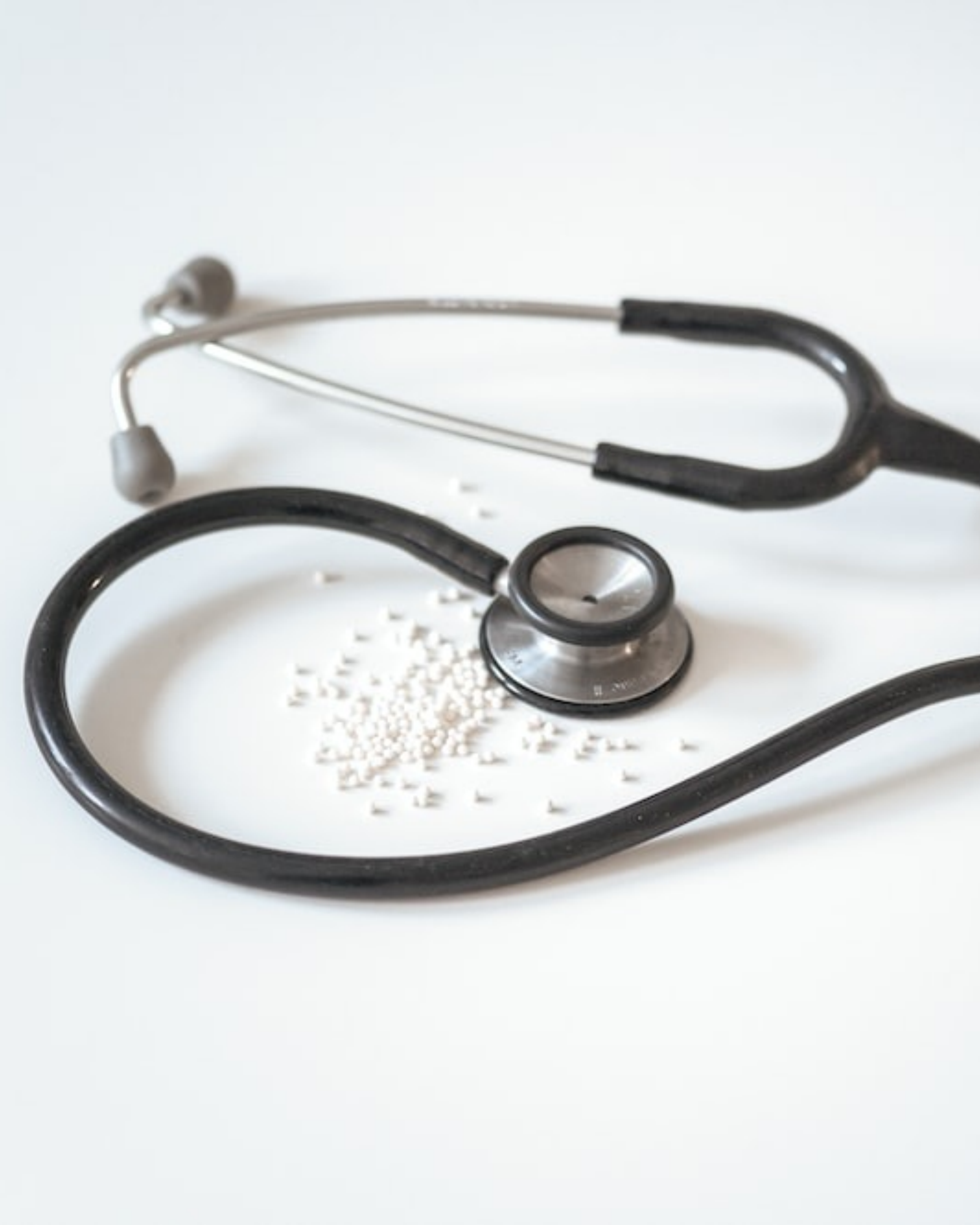

kimolq

içgörü

Boyun fıtığı mı migren mi?

MR testi sonucu alan kişiler, sonuçlarının boyun fıtığı ve boyun düzleşmesi mi yoksa migreni mi işaret ettiğini öğrenmek istiyorlar. Hissettikleri yoğun ağrıların nedenini öğrenmelerinin tedavi yöntemlerine olan güvenleri açısından önemli bir yere sahip.

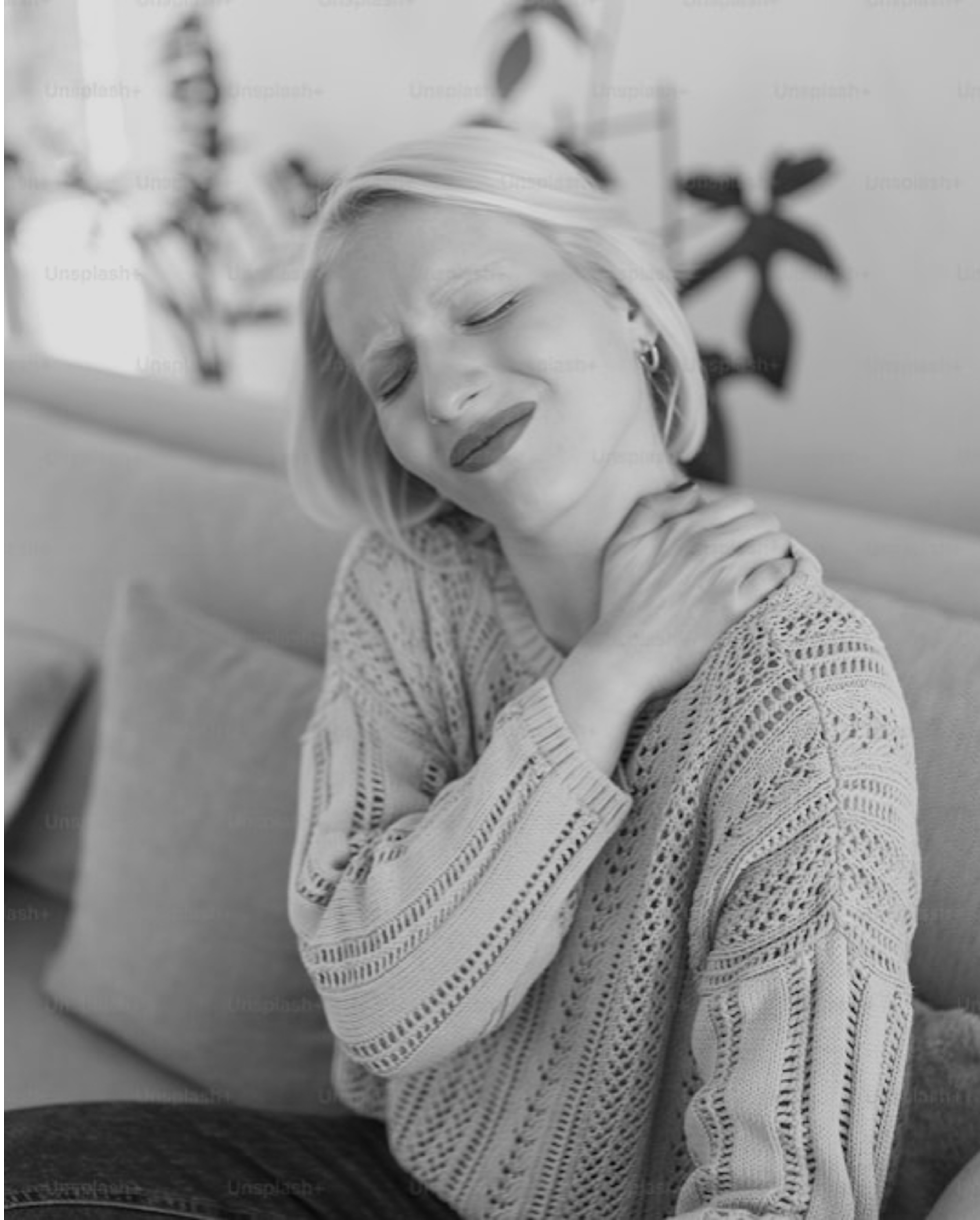

Tedavi Süreci

Tedavi Süreci

Ana Kategori

648 veri

Tedavi sürecine başlayan hastaların dörtte biri kullandıkları ilacın etkinliği hakkında geri bildirimde bulunurken, yine dörtte biri gebelik ve doğum sonrası süreçte ilaç tedavisi uygulanmayla ilgili sorular yöneltiyorlar.

Halihazırda ilaç tedavisi görmekte olan hastaların olası yan etkilerden, kullanılan başka ilaçlar veya varolan hastalıklarıyla olan etkileşiminden bahsetmelerinin yanı sıra ilaç tedavisinin dışındaki alternatif tedavi yöntemleri hakkında da araştırma yaptıkları görülüyor.

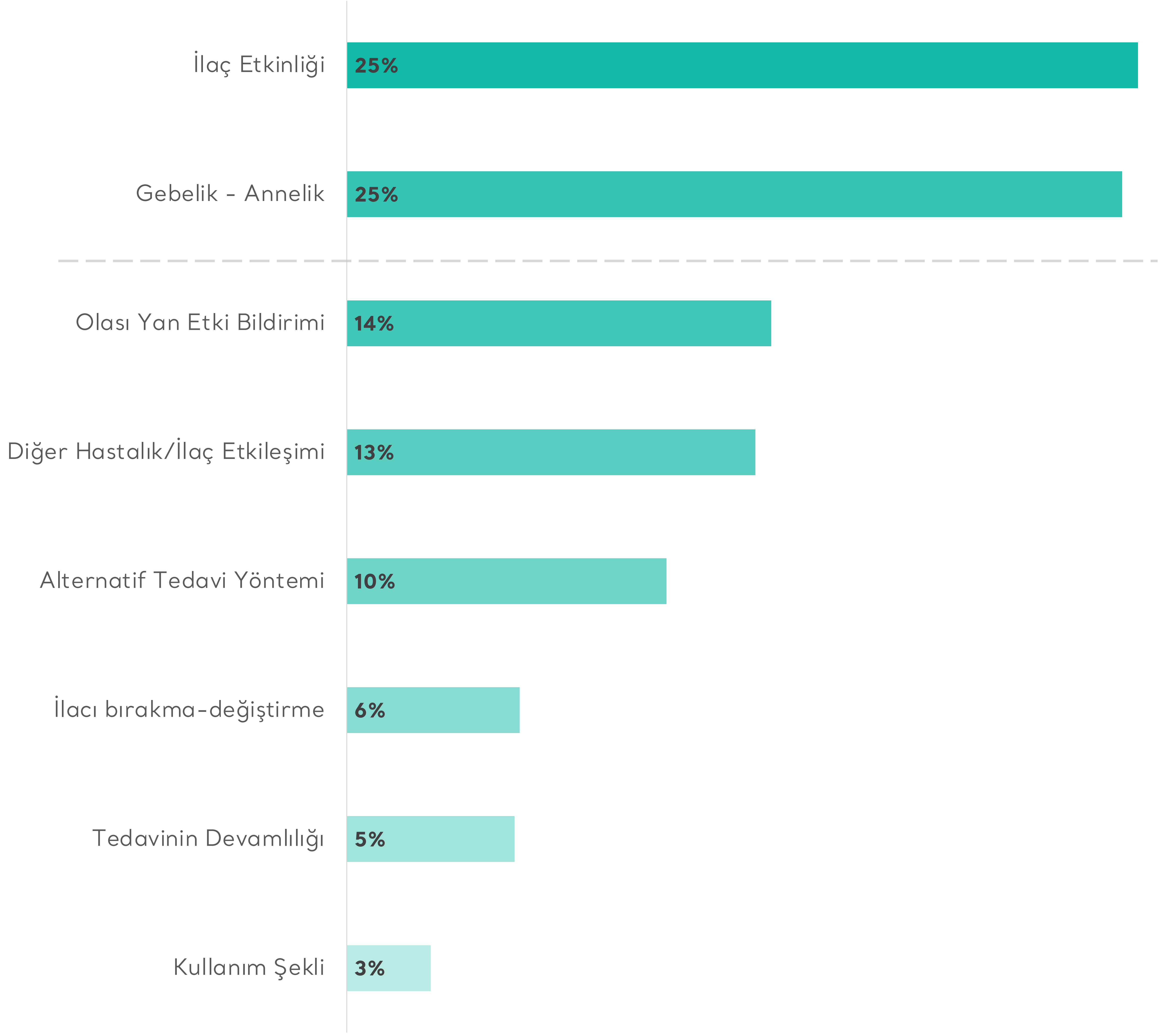

İlaç Etkinliği

Alt Kategori

158 veri

Her 10 hastadan 8'i kullandıkları ilaçların etkisini göremediklerini belirtirken, hastaların %9'u da yeterli süre ilaç kullanmalarına rağmen hala daha iyileşemedikleri yönünde düşüncelerini dile getirmektedirler.

Bir ilacın ne kadar süre içersinde etkisini göstereceği de hastaların konuştukları bir diğer başlıktır.

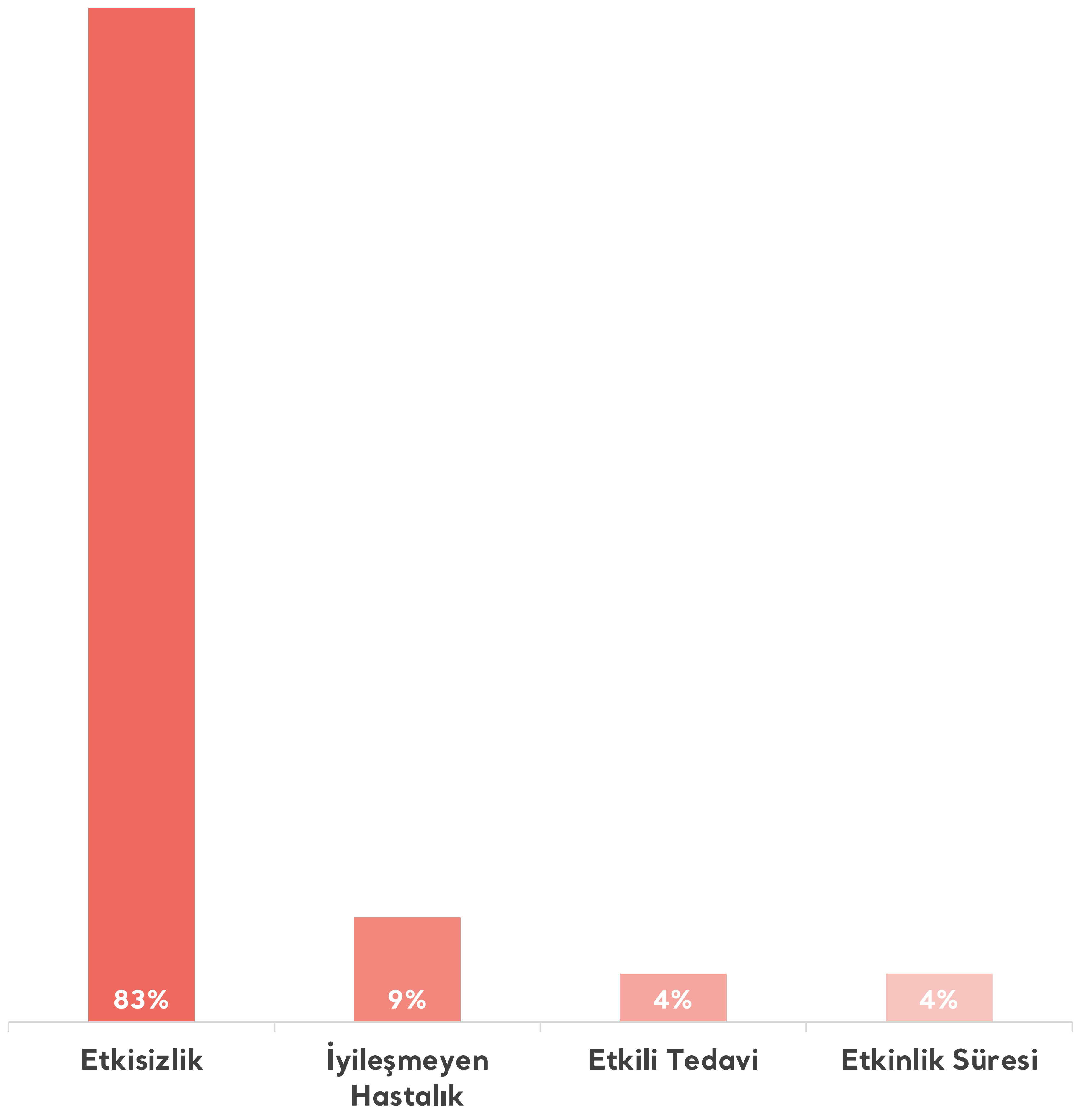

kimola

içgörü

Etkisiz kabul edilen ilacı değiştirmeye cesaret edemiyorlar.

Migren ilaçlarının aniden bırakılmasının yanlış olduğunun bilincindeki hastalar, ilaçları aniden bırakmak istemediklerini ama daha etkili bir diğer ilaca geçmek istedikleri yönünde bir ikilem yaşıyorlar. Bu sorun hastaların baş ağrılarıyla uzun süre mücadele etmesine neden olarak ilaçlara olan güveni sarsabilir.

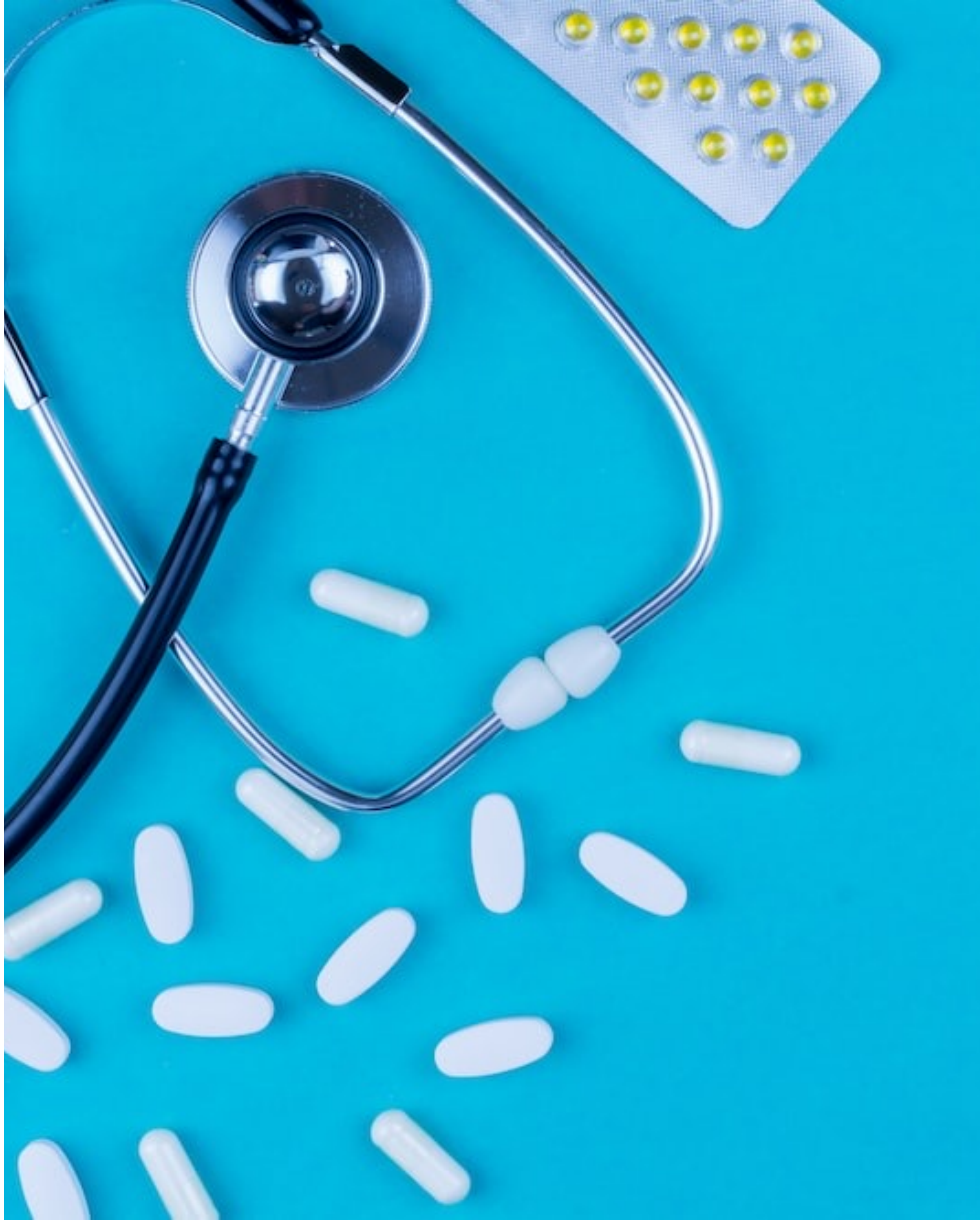

kimola

içgörü

"Yok mu bunun kesin bir çözümü?"

Hastalar düzenli olarak ilaç kullanmalarının sonucunda belli bir süre sonra migrenden tamamen kurtulmak istiyorlar. Hastalığın bir süre sonra iyileşmesini bekleyen kişiler, kesin çözümü bulana kadar ilaç kullanmak istemediklerini vurguluyorlar.

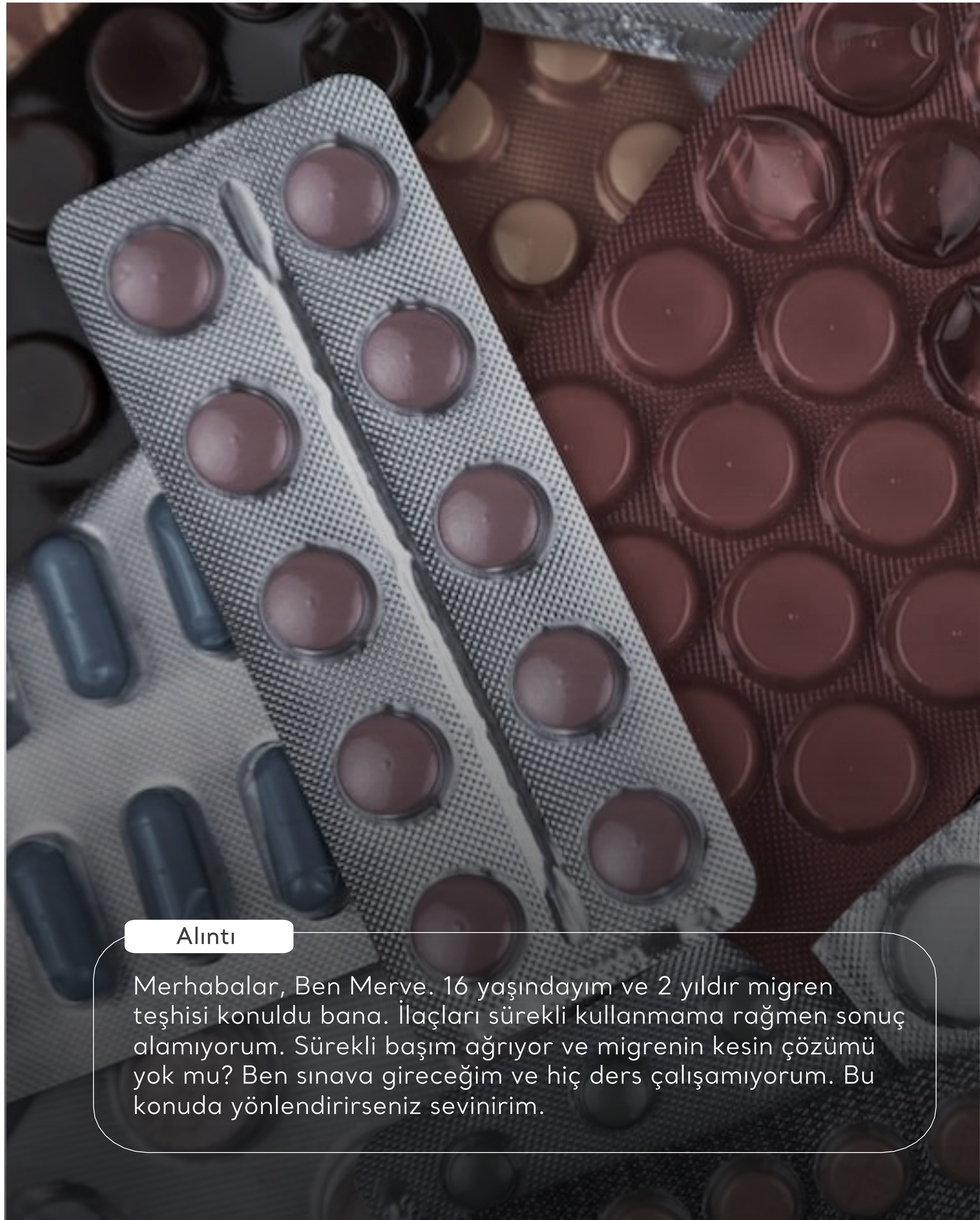

kimolq

içgörü

Doktorlar, Parol'un zararsızlığını vurguluyorlar.

Hamile ve emziren kadınlar arasında Parol kullanmanın yaygın olduğu görülüyor. Hekimlerin migren atağı geçiren bebekli kadınların Parol kullanmalarında bir sıkıntı olmadığına ve bebeklerine zarar vermeyeceklerine dair cevaplar verdikleri de gözlemlenmekte.

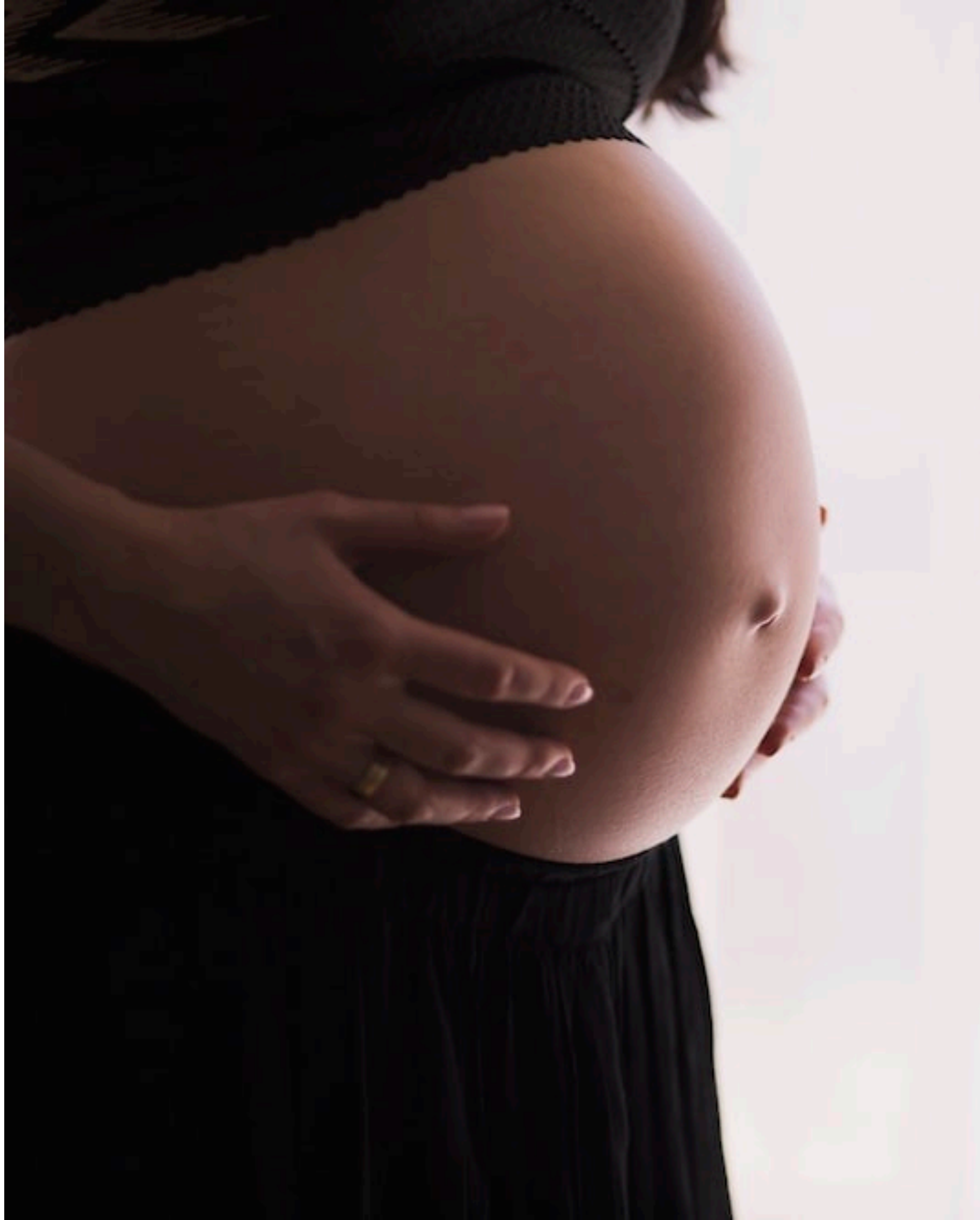

kimola

içgörü

Doğum kontrol hapları ve migren ilaçlarının etkileşimi.

Halihazırda doğum kontrol hapı kullanan kadınların migren ilaçlarıyla birlikte kullanması durumunda oluşacak olan etkileşimler ve olası yan etkileri merak ettikleri ve bu konuda endişeleri olduğu görülüyor.

Bazı kadın migren hastaları, doğum kontrol haplarını migren ilaçlarına tercih edeceklerini belirtiyorlar.

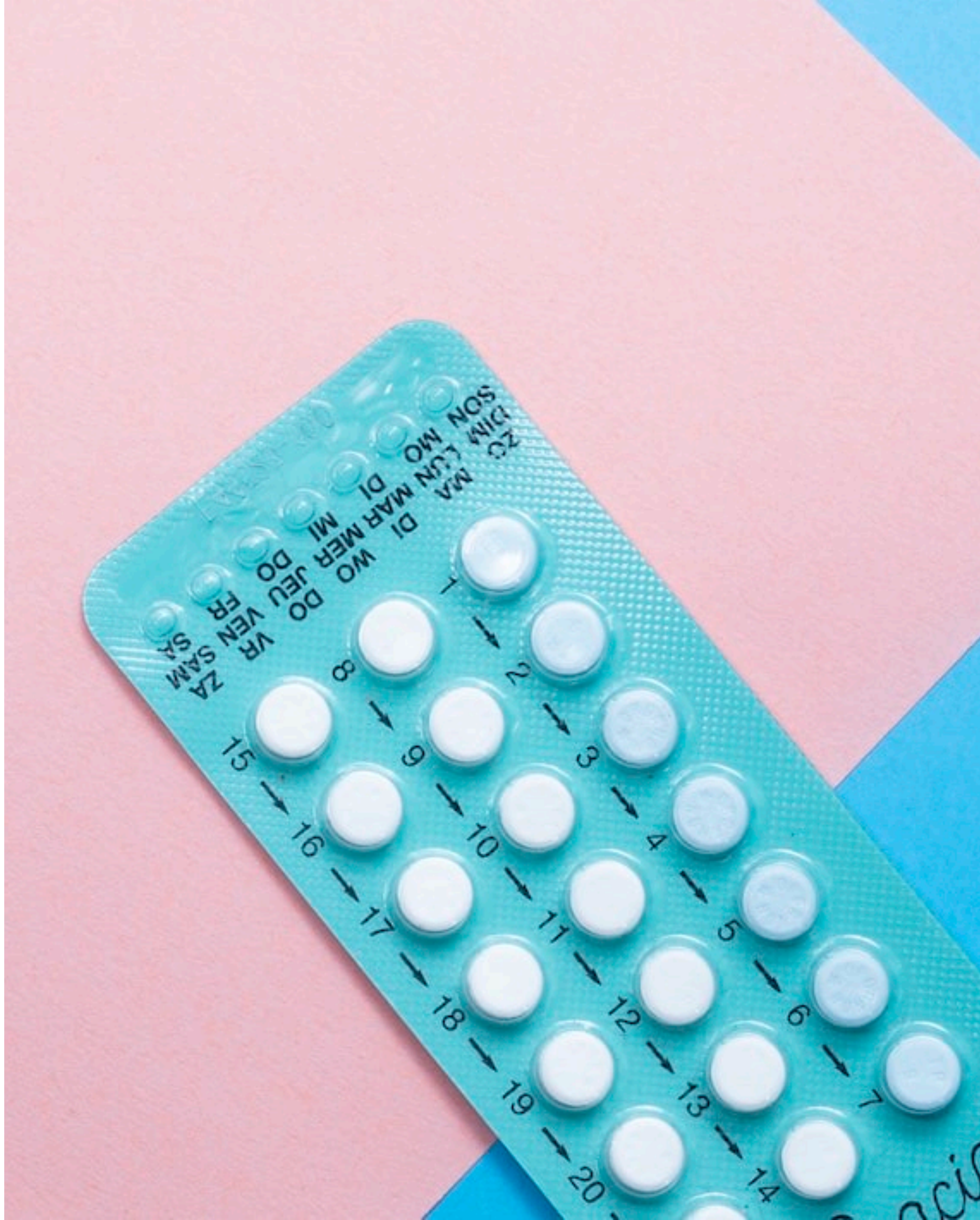

Olası Yan Etki Bildirimi

Hastalar tarafından bildirilen olası yan etkiler sınıflandırıldığında, bilişsel yeteneklerde zorlama (uyku hali, halsizlik, yorgunluk vb.), ağrı yoğunluğu ve çeşitlerinde artma, vücut fonksiyolarına etki (aşırı terleme, ağızda kuruluk vb.) başlıklarının üçte birlik oranlarda konuşulduğu gözlemleniyor. Ayrıca kilo problemleri ve vücutta uyuşma da verilerin neredeyse beşte birini kapsamaktadır.

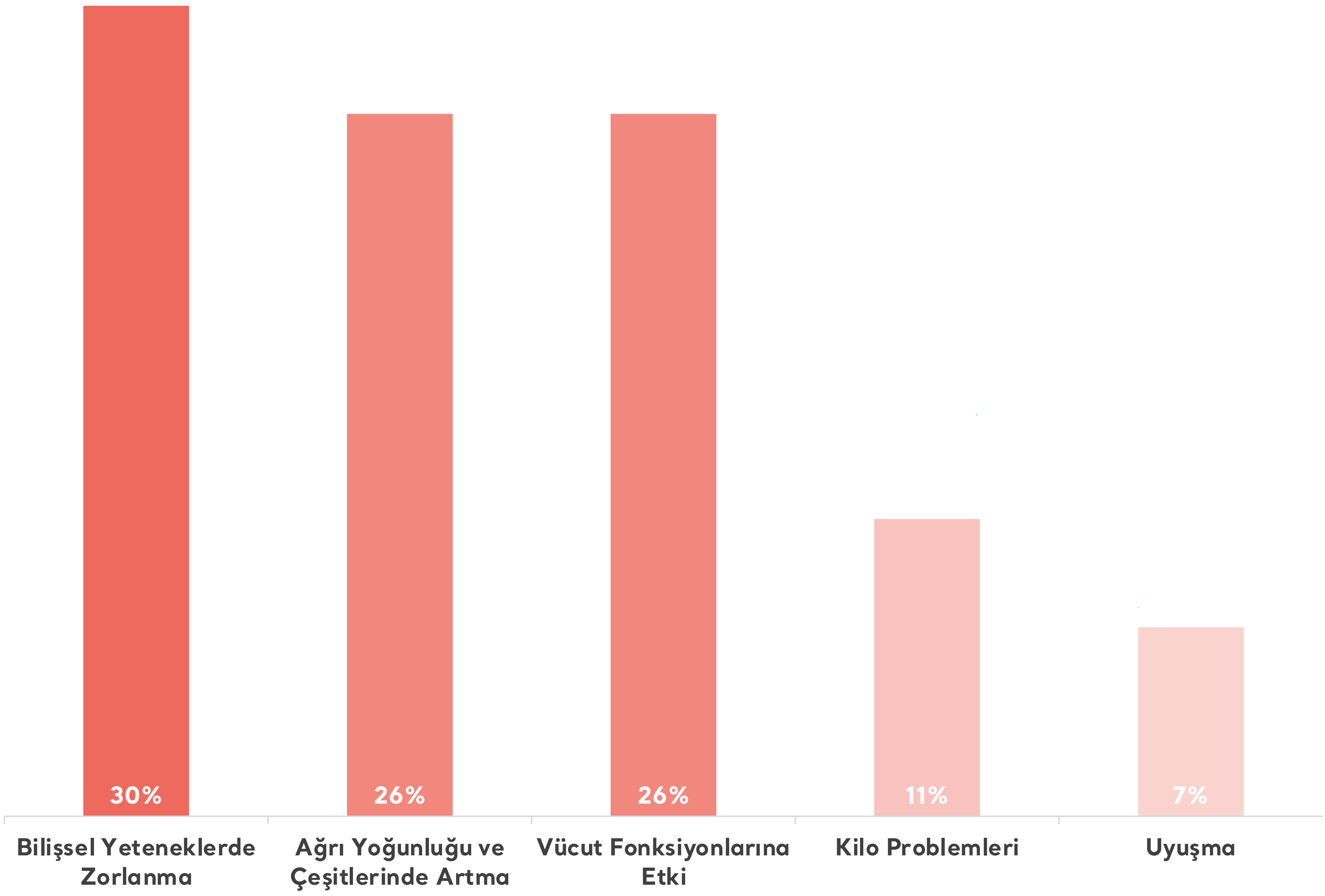

kimola

içgörü

Migren ilaçlarının ağrılarını daha da arttırması önemli bir geribildirim.

Migren ilacı kullanan hastaların bazıları gözlemledikleri olası yan etkileri, muayene oldukları doktorlardansa kısa sürede cevap alabildikleri çevrimiçi hizmet veren doktorlara sormayı tercih ediyorlar. Tedavi sürecindeki hastaların ağrılarında artış gözlemlemeleri alternatif tedavi yöntemlerine ilgiyi arttırıyor.

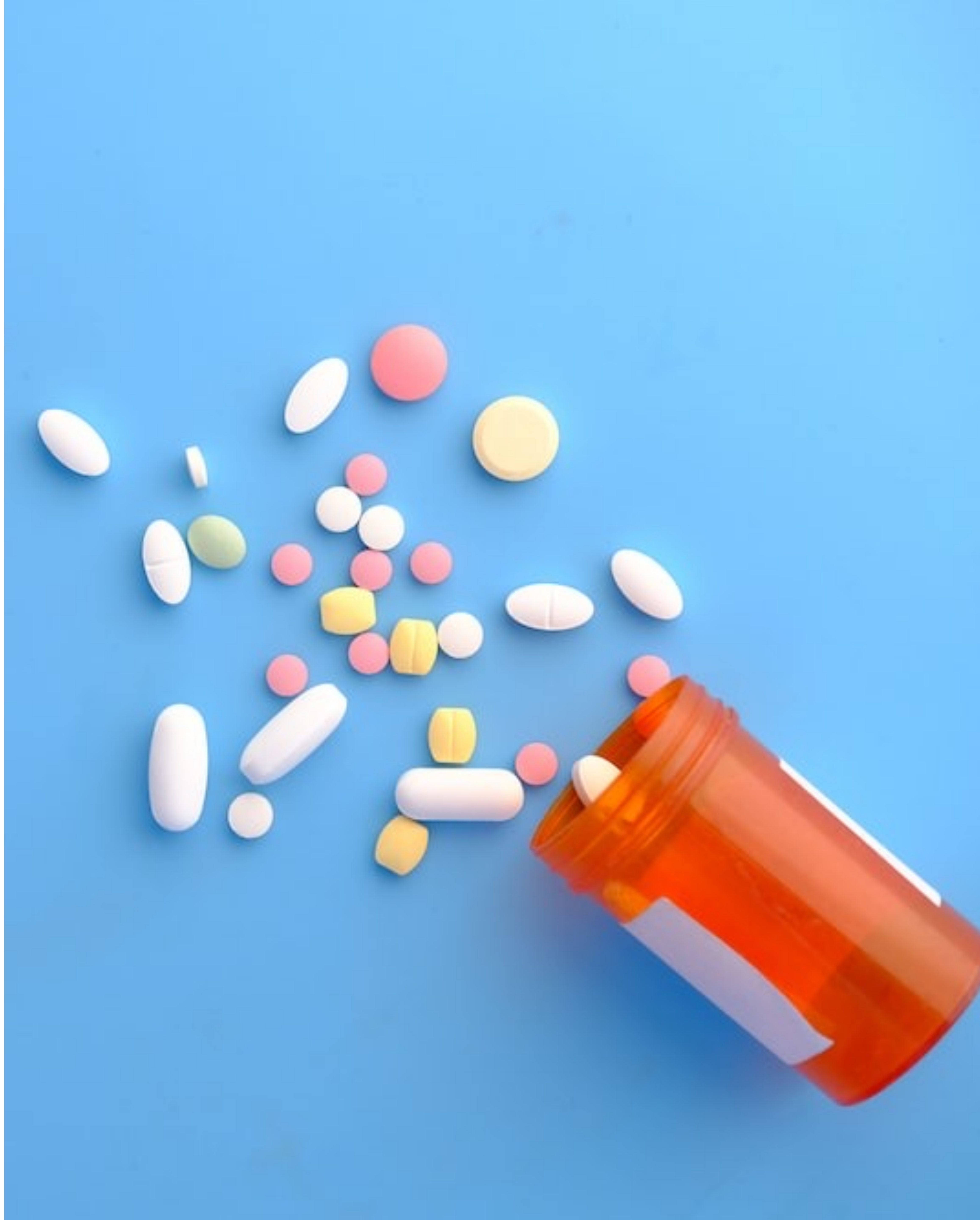

kimola

içgörü

Ağrıdan kurtulmak için bitkinliği göze almak gerekiyor.

Migren ataklarının günlük yaşamlarına oldukça fazla etki ettiğini belirten hastalar, kullandıkları ilaçların ekstra olarak halsizlik, yorgunluk ve uyuşukluk gibi odaklanma problemleri yaratabilecek yan etkilere neden olması, özellikle çalışan hastaların ilaç kullanmaya karşı negatif tutum sergilemesine neden olabilir.

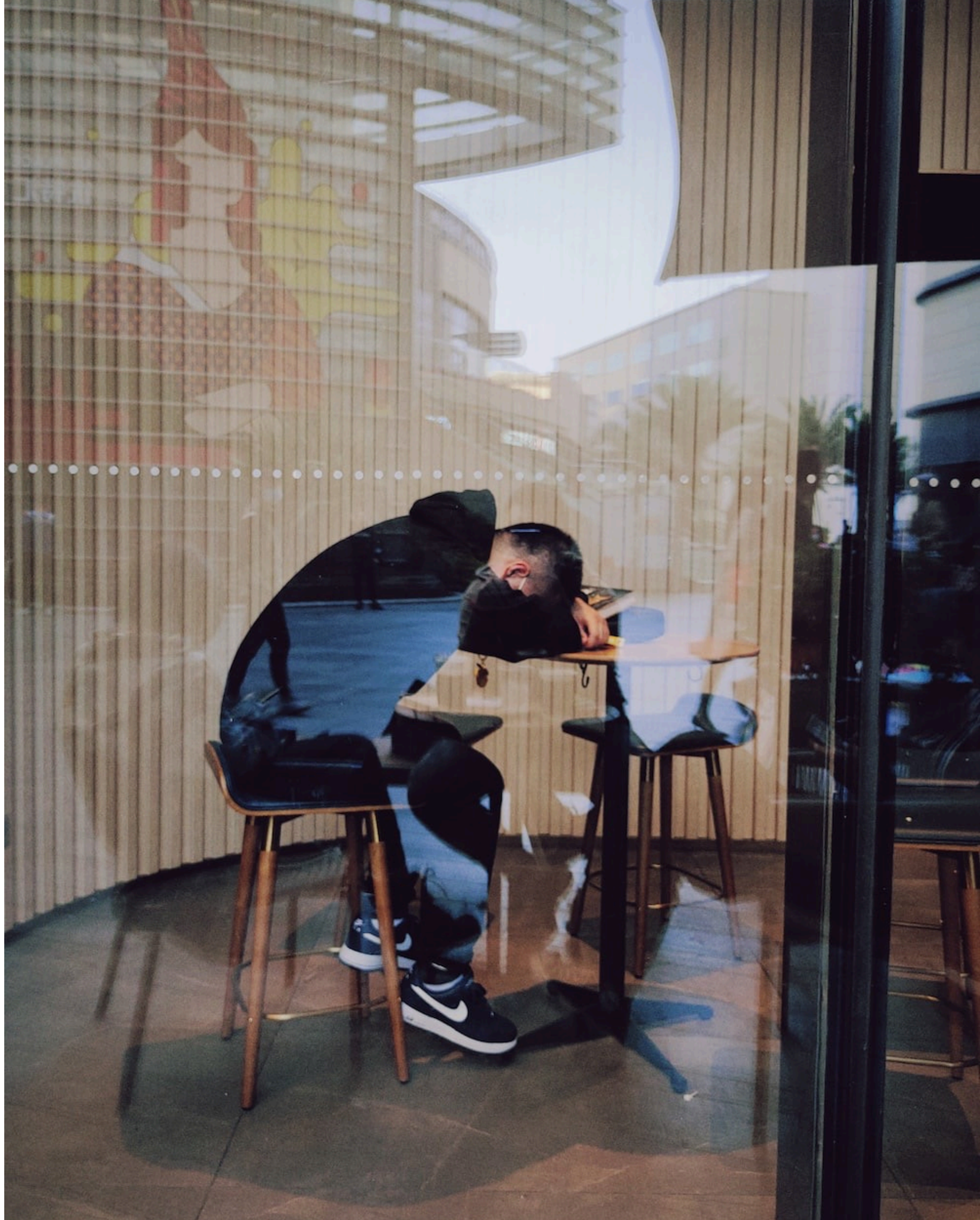

Hastalık Deneyimi

Hastalık Deneyimi

Ana Kategoriler

347 veri

Migren tanısı konmuş hastalar, günlük yaşamlarında hissettikleri psikolojik ve fiziksel etkilerin yanı sıra sosyal yaşamda yaşadıkları zorluklar hakkında konuşmaktadırlar.

Bu veriler sınıflandırıldığında verinin üçte birinden fazlası fiziksel etkileri içerdiği görülürken, psikoljik etkiler %23, sosyal yaşamla ilgili verilerse %8 orana sahiptir.

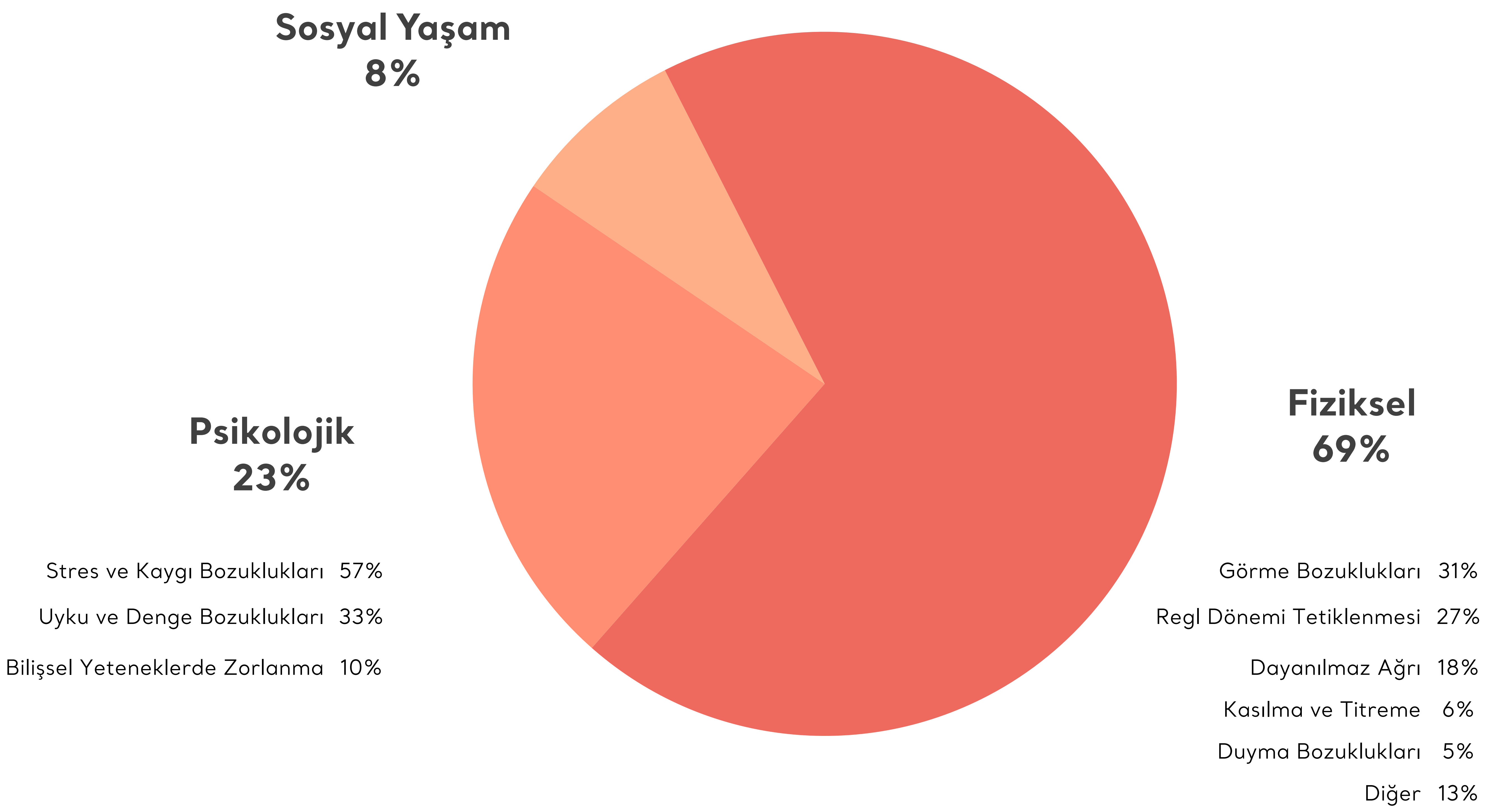

kimolq

içgörü

Auralı migren, günlük hayatı en fazla etkileyen migren türü.

Auralı migren tanısı konmuş hastaların, günlük yaşamlarında görüşlerini etkileyen semptomlar nedeniyle şikayetlerini dile getiriyorlar. Gözlerinin önünden gitmeyen lekeler, ışık çakması adı verilen parlamalar gibi durumların yaşanması, günlük hayatlarını zorlaştırıyor.

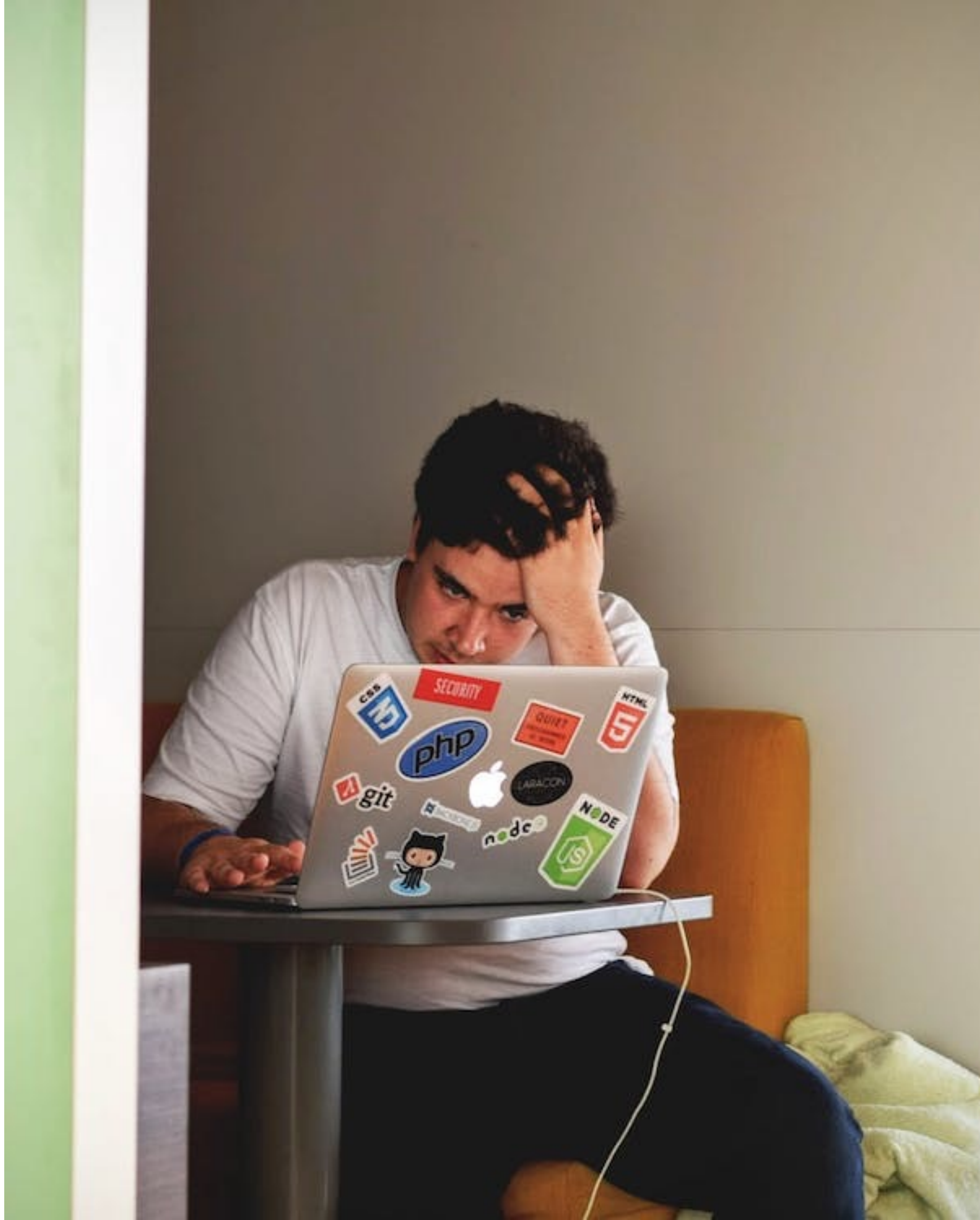

kimolq

içgörü

Menstrüel migren, kadınların en fazla danıştığı konular.

Kadınlar regl dönemlerinde klasik bir baş ağrısından ziyade uzun süren ve yoğun yaşadıkları migren ataklarına kesin çözüm arıyorlar. Hatta kesin çözüm sunmayan tıbbi tedavi yöntemleri nedeniyle alternatif tıbba da yöneldikleri görülüyor.

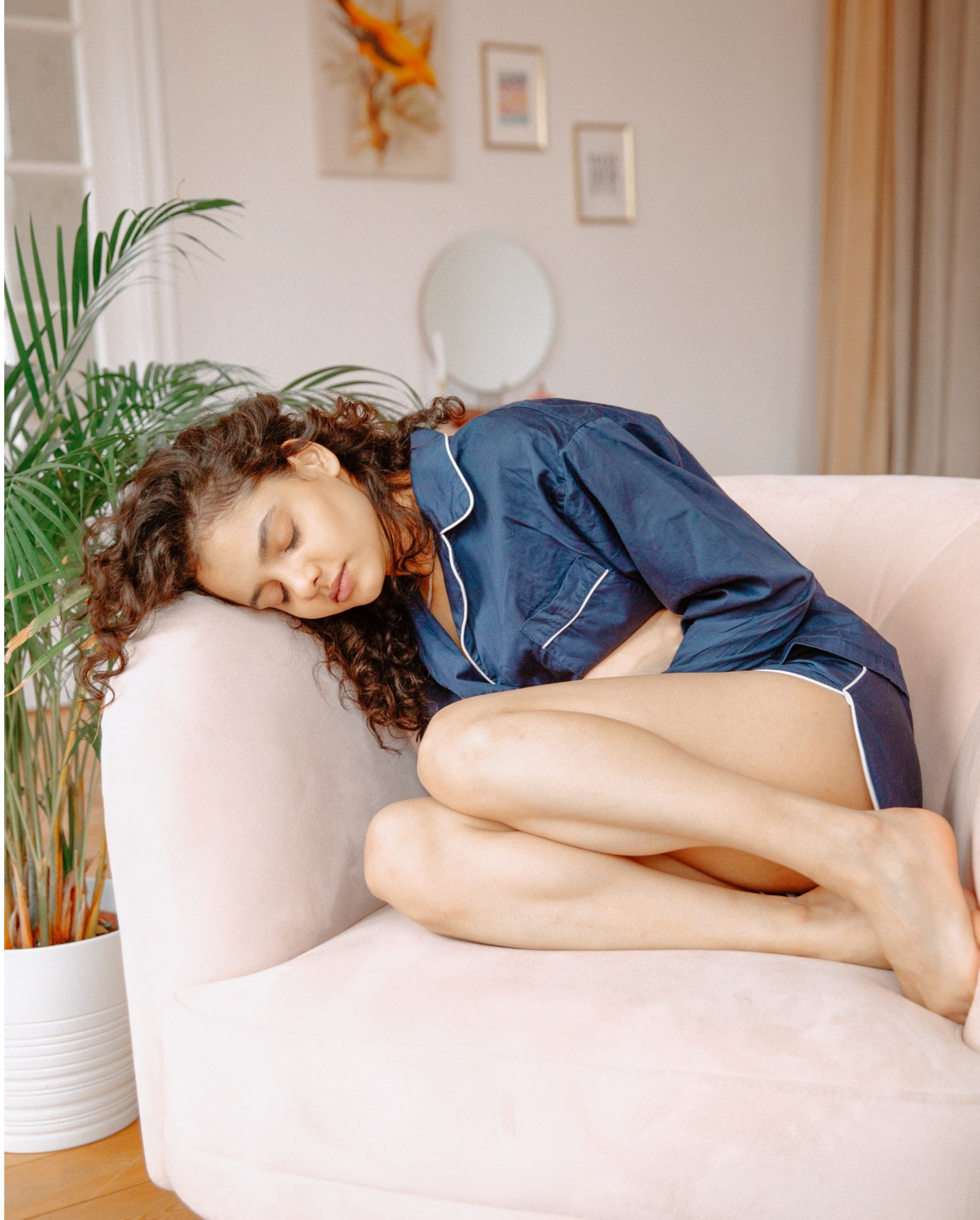

kimola

içgörü

"Felç geçirir miyim?"

Migren atakları ile birlikte kol, bacak ve boyun bölgelerinde kasılma ve titreme, beyinde zonklama gibi etkiler gözlemleyen hastalar, migrenin geçmeme durumunda felç geçirme ihtimalleri üzerinden endişe yaşamaktadırlar.

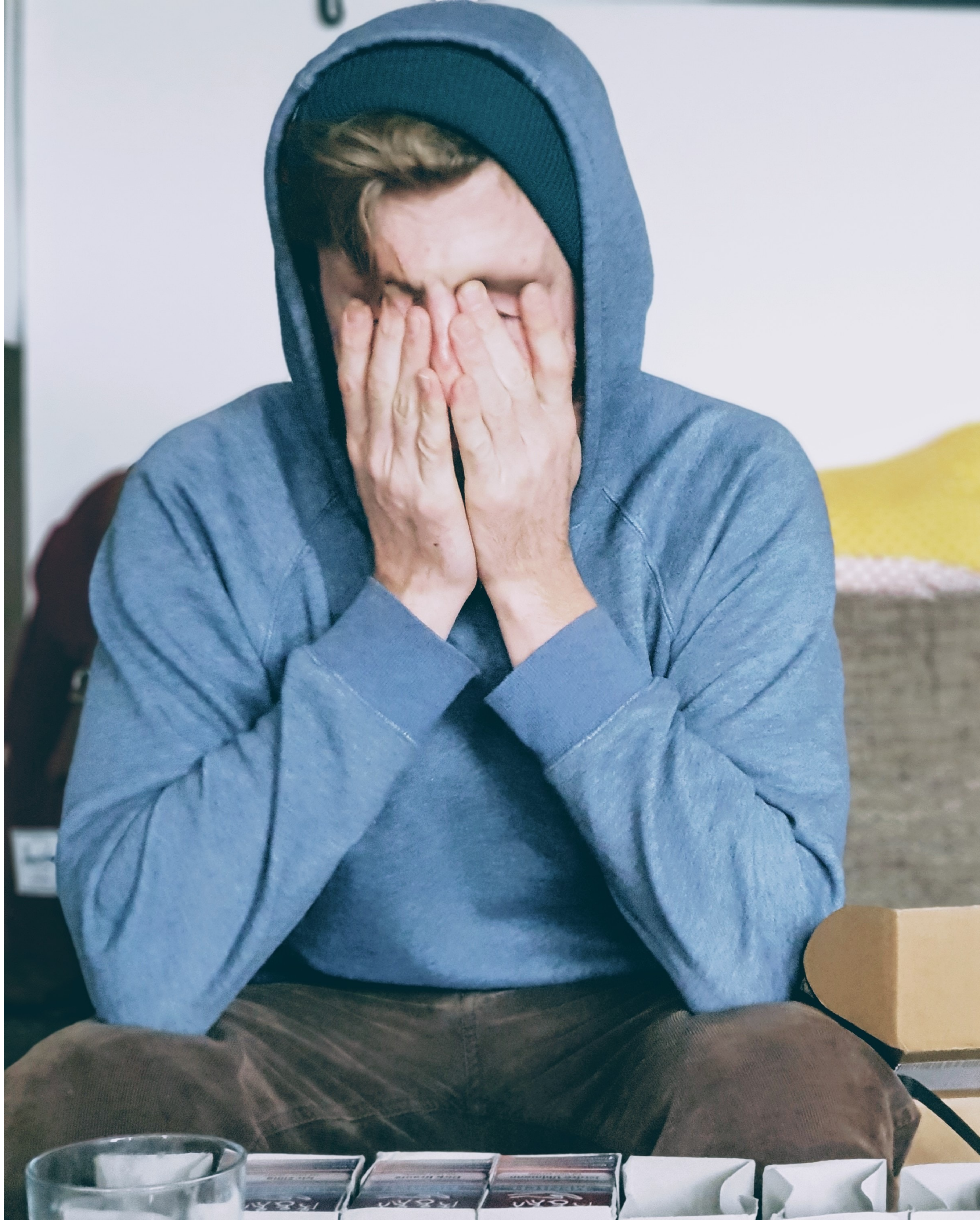

kimolq

içgörü

Sınav dönemlerinde öğrenci şikayetleri artıyor.

Sınav stresi, birçok hastalıkta olduğu gibi öğrencilerin sıklıkla migren atağı yaşadıkları bir dönem olmaya devam ediyor. Öğrenciler, hastalığın kesin tedavisini aramasalar da, en azından hazırlandıkları sınavlara herhangi bir ağrı hissetmeden ve odaklanarak girmek istiyorlar.

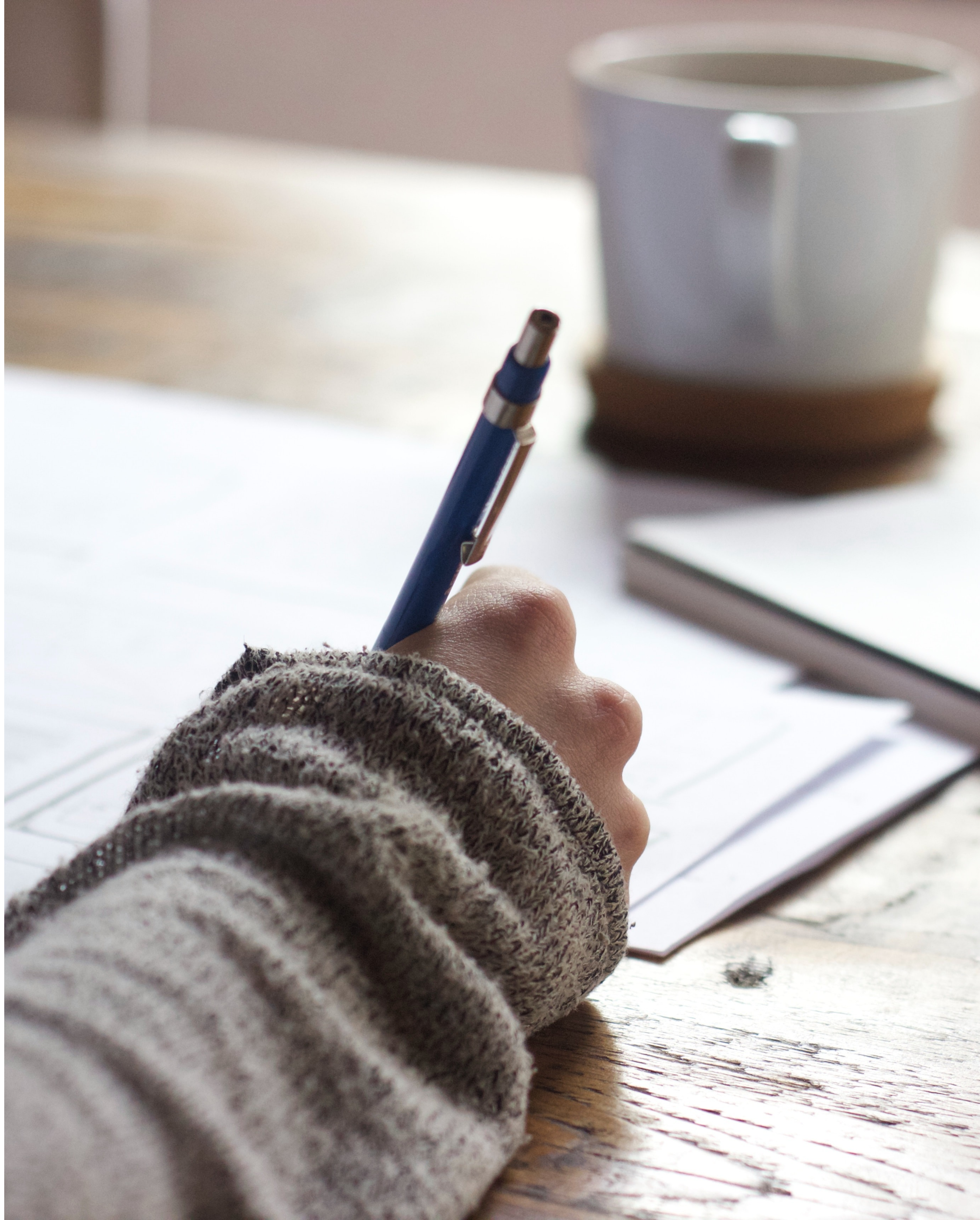

Tedavi Sonrası

Tedavi Sonrası

Ana Kategoriler

126 veri

Tedavi sürecini tamamlayan hastaların üçte ikisi, ilaç tedavisi ile tam anlamıyla iyileşmediklerini belirterek akupunktur, botoks vb. alternatif tedavi yöntemlerini araştırırken üçte biri de tekrardan gözlemledikleri semptomlar hakkında araştırma yapmaktadırlar.

%4 oranında da olsa bazı hastalar, migrenleri iyileşse de başka hastalıkları için tedavi araştırırken migrenle olan etkileşimi açısından bu durumu belirtiyorlar.

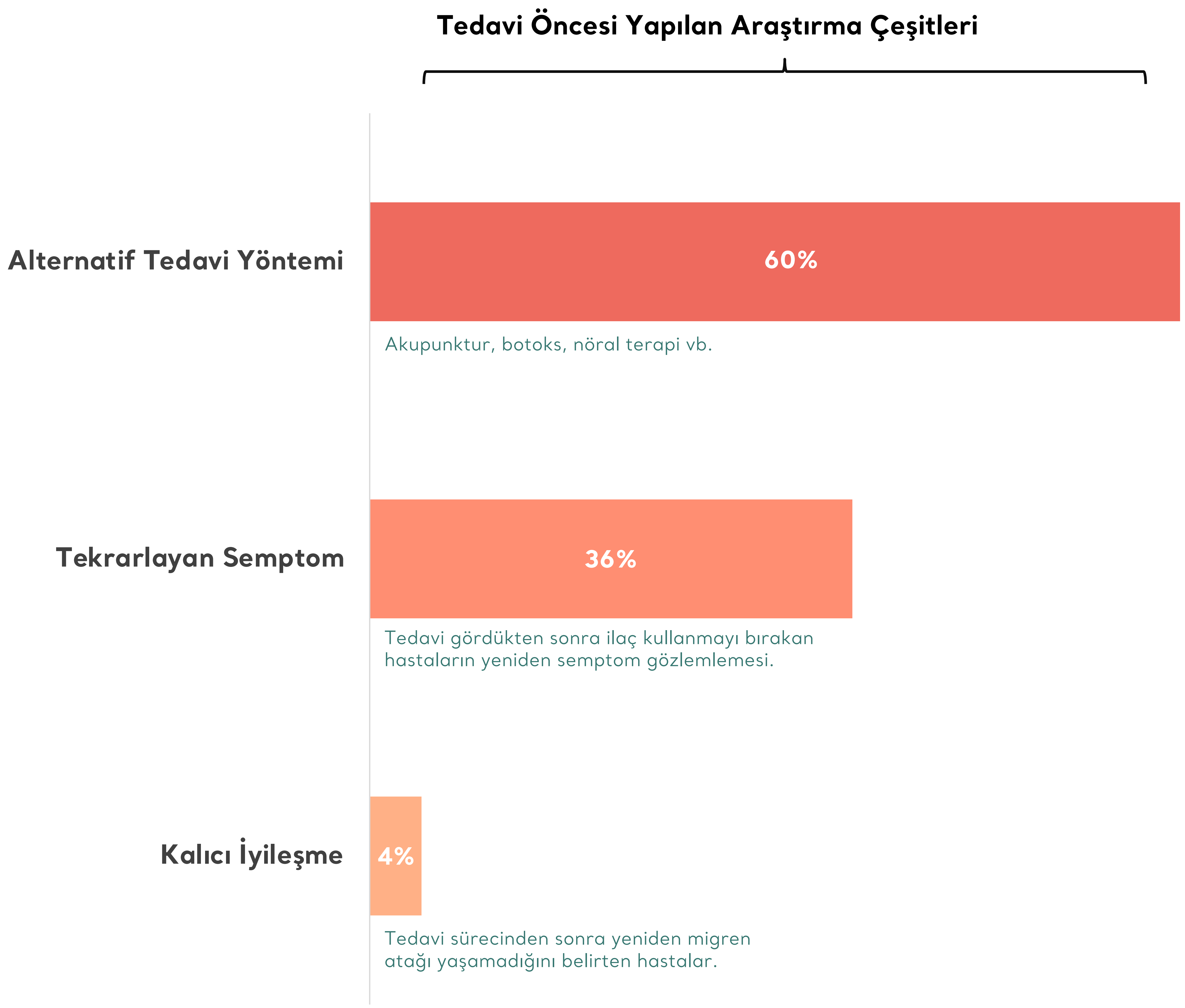

kimola

içgörü

Ağrı geçtikten hemen sonra ilacı bırakıyorlar.

İlaç tedavisi süren hastalar, hissettikleri migren ağrısının hafiflemesi ya da tamamen geçmesinin ardından ilaçlara ara veriyorlar ve ilerleyen süreçte bu semptomların tekrarladığı bir diğer ana kadar ilaç kullanmaya ara veriyorlar.

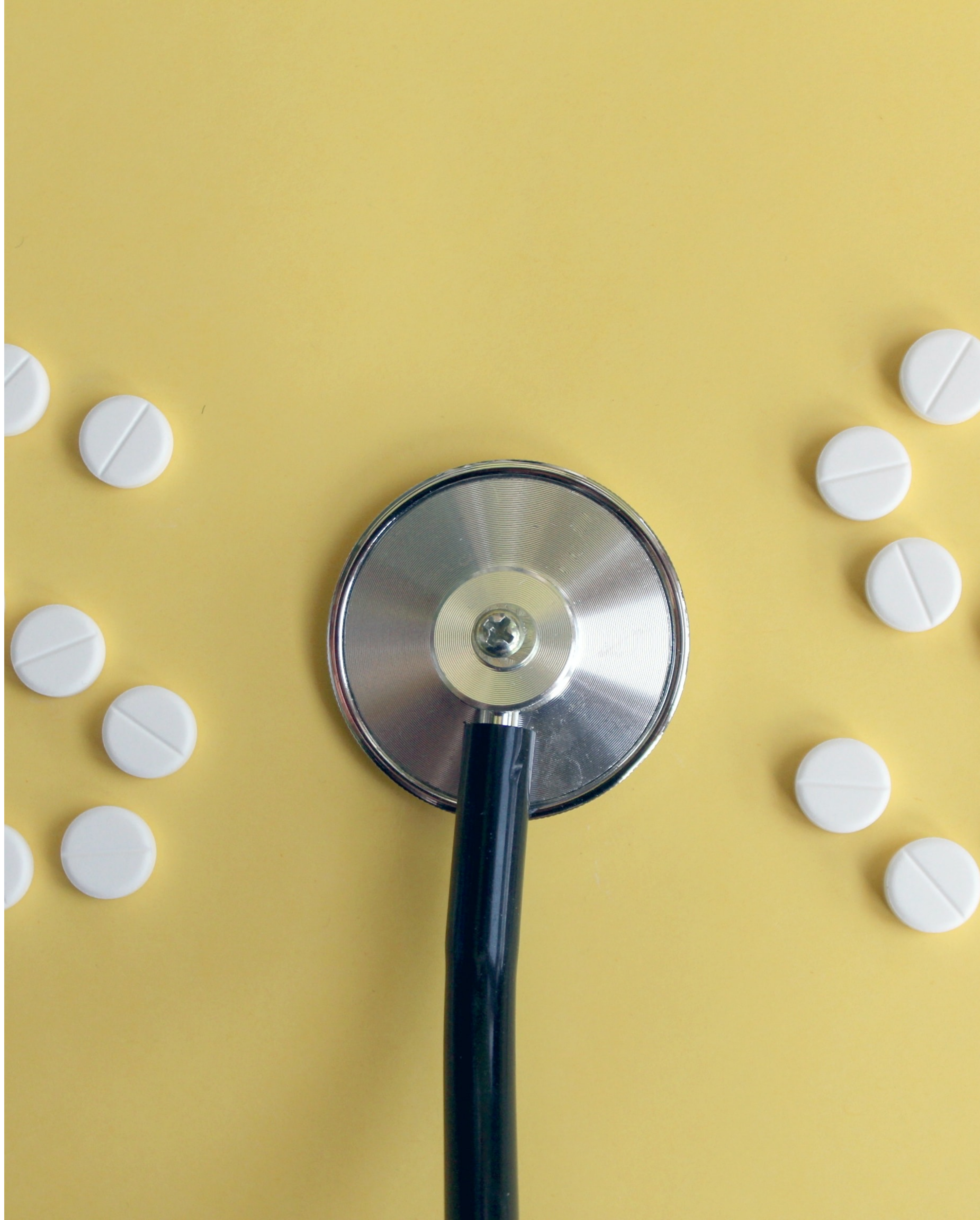

kimolq

Haziran, 2023
