# Supplementary material for: Migraine care in the digital era: social listening insights and neurologists’ perspectives from Türkiye
Source: Front Digit Health. 2026 Sep 14;8:1891640. doi: 10.3389/fdgth.2026.1891640 (PMC13617366; doi:10.3389/fdgth.2026.1891640)
Supplement: Supplementary file 2 [file Datasheet2.docx]

Supplementary Material 2

**English summary of the Kimola social listening report on migraine in Türkiye**

This supplementary document summarizes the original Turkish Kimola social listening report prepared in June 2023. It is intended to support English-language review of the source report and does not represent an independent re-analysis of the underlying raw online platform data.

# 1 Source report and scope

The source report was prepared by Kimola for a migraine-focused social listening analysis. The dataset was derived from Doktorsitesi.com, a Turkish online health platform where users can submit health-related questions to physicians. The report states that 2,500 migraine-related user-generated entries were collected across 11 medical specialties and analyzed after automated and manual filtering by Kimola’s Insight and Research Team.

Kimola classified the dataset into four predefined stages of the patient journey: pre-treatment phase, treatment process, post-treatment phase, and disease experience. The report used machine learning-supported classification and social research methods to organize patient-generated content into thematic categories. The present manuscript used the report as a pre-existing, pre-processed analytical output.

# 2 Overall dataset profile

The final dataset comprised 2,500 migraine-related user-generated entries. The largest proportion of content was related to the pre-treatment phase, followed by the treatment process, disease experience, and post-treatment phase.

| **Patient journey stage** | **Reported proportion of dataset** | **Reported entry count in source report** |
| --- | --- | --- |
| Pre-treatment phase | 56% | 1,379 |
| Treatment process | 27% | 648 |
| Disease experience | 13% | 347 |
| Post-treatment phase | 4% | 126 |

Note. Entry counts are reported as presented in the source report; minor differences from the total may reflect rounding or reporting conventions in the original output.

Most entries were posted by patients themselves (91%). Caregiver-generated entries accounted for 9% of the dataset and were primarily contributed by parents, children, spouses, siblings, or friends.

| **Contributor category** | **Reported proportion** |
| --- | --- |
| Patients | 91% |
| Caregivers | 9% |
| Parent among caregiver entries | 32% |
| Child among caregiver entries | 30% |
| Spouse among caregiver entries | 24% |
| Sibling among caregiver entries | 7% |
| Friend among caregiver entries | 4% |

# 3 Pre-treatment phase

The pre-treatment phase represented the largest category in the source report. The report separated pre-treatment content into two major domains: research/information seeking (71%) and diagnostic discussions (29%).

| **Pre-treatment domain** | **Subtheme** | **Reported proportion** |
| --- | --- | --- |
| Research/information seeking | Symptom research | 68% |
| Research/information seeking | Treatment or medication research | 28% |
| Research/information seeking | Possible adverse effect research | 5% |
| Diagnostic discussions | MRI result interpretation | 67% |
| Diagnostic discussions | Questioning the physician’s diagnosis | 31% |
| Diagnostic discussions | Other test results | 2% |

Key insights from this stage indicated that users often confused migraine with nonspecific headache, questioned whether migraine was a disease or a symptom of a more serious problem, searched for hereditary risk, and expressed uncertainty about visual or sensory symptoms. The report also described anxiety about medical evaluation, reluctance to seek care until symptoms became severe, and concerns about starting pharmacological treatment before understanding possible adverse effects.

Diagnostic discussions were often linked to online interpretation of magnetic resonance imaging (MRI) or other test results before returning to the treating physician. Some users sought confirmation of migraine diagnoses and expressed concern that migraine might be a placeholder diagnosis or that their symptoms could indicate a more serious neurological condition.

# 4 Treatment process

The treatment process category included 648 entries and focused on treatment effectiveness, pregnancy or motherhood, adverse effects, comorbidities or drug interactions, non-oral, adjunctive, or complementary treatment options, discontinuation or switching, continuity of therapy, and mode of use.

| **Treatment-process topic** | **Reported proportion** |
| --- | --- |
| Medication effectiveness | 25% |
| Pregnancy and breastfeeding | 25% |
| Adverse effect or tolerability concerns | 14% |
| Comorbidities or drug interactions | 13% |
| Treatment options | 10% |
| Treatment discontinuation or switching | 6% |
| Treatment continuity | 5% |
| Mode of use | 3% |

Within the medication effectiveness subcategory, most entries described perceived lack of effect. The source report stated that 83% of entries in this subcategory referred to ineffectiveness, while smaller proportions referred to persistent disease despite treatment, effective treatment, or questions about how long a medication should take to work. The report interpreted this as reflecting an expectation of rapid relief and a tendency to interpret delayed benefit as treatment failure.

Pregnancy and breastfeeding discussions focused on the safety of migraine medication during pregnancy planning, pregnancy, and postpartum or breastfeeding periods. Users often asked whether commonly used analgesics or migraine-related medications could harm the fetus or infant. Some entries reflected concerns about interactions between contraceptive choices and migraine medications.

Adverse effect and tolerability entries included cognitive difficulty, fatigue, sleepiness, worsening pain, effects on bodily functions, weight concerns, and numbness. The report highlighted that adverse effects affecting concentration and daily functioning may create barriers to treatment continuation, particularly among working-age individuals.

# 5 Disease experience

The disease experience category captured the broader burden of migraine beyond treatment-specific questions. It was divided into physical, psychological, and social dimensions.

| **Disease-experience domain** | **Reported proportion** | **Subthemes reported in the source report** |
| --- | --- | --- |
| Physical | 69% | Visual disturbances (31%); menstrual triggers (27%); severe pain (18%); muscle contraction/tremor (6%); sensory/hearing disturbances (5%); other (13%) |
| Psychological | 23% | Stress and anxiety (57%); sleep and balance disturbances (33%); cognitive difficulties (10%) |
| Social | 8% | Social and functional burden |

The report highlighted that aura-related visual symptoms, menstrual migraine, fear of severe neurological consequences, difficulty accepting antidepressant use for migraine, and exam-related stress among students were recurring patient concerns. These findings suggest that migraine was discussed not only as episodic pain but also as a recurring source of functional, psychological, and social burden.

# 6 Post-treatment phase

The post-treatment phase was the smallest category in the source report but provided insight into longer-term behavior after treatment exposure. The report labeled the most frequent category as alternative treatment methods and included examples such as acupuncture, onabotulinumtoxinA/Botox, and neural therapy. For clinical interpretation, this category is best understood as treatment-seeking beyond conventional oral pharmacotherapy because it includes both complementary approaches and evidence-based procedural or adjunctive options.

| **Post-treatment topic** | **Reported proportion** | **Description** |
| --- | --- | --- |
| Treatment-seeking beyond conventional oral pharmacotherapy | 60% | Entries referring to acupuncture, onabotulinumtoxinA/Botox, neural therapy, or other approaches beyond conventional oral pharmacotherapy |
| Recurrent symptoms | 36% | Entries describing recurrence after discontinuing medication following symptom improvement |
| Sustained improvement | 4% | Entries describing no recurrent migraine attacks after treatment |

The source report interpreted recurrence after stopping medication as a recurrent pattern in which patients paused treatment once migraine pain improved and resumed searching when symptoms returned. This pattern is relevant to treatment persistence, expectation-setting, and long-term disease management.

# 7 Overall interpretive summary

Across stages, the source report suggests that digital patient-generated content reflects a broad range of unmet needs in migraine care: uncertainty about symptom meaning, limited migraine literacy, diagnostic reassurance-seeking, fear of medication adverse effects, pregnancy-related safety concerns, perceived lack of treatment effectiveness, and treatment discontinuation after short-term improvement.

The report also indicates that digital environments may shape patient expectations before, during, and after clinical encounters. Patients used online physician-question platforms not only to obtain information but also to seek reassurance, compare treatments, interpret diagnostic results, and decide whether to continue, stop, switch, or seek non-oral, adjunctive, procedural, or complementary options.

# 8 Important notes on interpretation

- This supplementary file is an English summary of the source report, not a full translation of every slide or entry.
- The authors of the manuscript did not have direct access to the raw online platform dataset.
- The original preprocessing, coding, and theme-generation procedures were performed by Kimola and were not independently reproducible from the summary report alone.
- Percentages are reported as presented in the source report and should be interpreted descriptively.
- The post-treatment category was labeled as “alternative treatment methods” in the source report; however, it included heterogeneous approaches, including complementary methods and evidence-based procedural or adjunctive options. Therefore, clinically neutral wording was used in this summary where appropriate.
