# Supplementary material for: Migraine care in the digital era: social listening insights and neurologists’ perspectives from Türkiye
Source: Front Digit Health. 2026 Sep 14;8:1891640. doi: 10.3389/fdgth.2026.1891640 (PMC13617366; doi:10.3389/fdgth.2026.1891640)
Supplement: Supplementary file 3 [file Datasheet3.docx]

Supplementary Material 3

**Physician survey questionnaire and aggregated responses**

This supplementary material presents the structured physician survey questionnaire and aggregated responses from 10 neurologists with expertise in migraine and headache disorders in Türkiye. Responses are reported as percentages and, where applicable, as n/10 to improve transparency given the small sample size. Open-ended responses are presented in anonymized form.

# 1 Likert-scale questions and aggregated responses

Likert-scale response categories are presented as Strongly agree, Agree, Neutral, Disagree, and Strongly disagree. Neutral corresponds to neither agree nor disagree.

| **No.** | **Statement** | **Strongly agree** | **Agree** | **Neutral** | **Disagree** | **Strongly disagree** |
| --- | --- | --- | --- | --- | --- | --- |
| 1 | Many migraine patients in Türkiye rely on social media as a major source of information about their condition. | 30% | 40% | 20% | 10% | 0% |
|  |  | (3/10) | (4/10) | (2/10) | (1/10) | (0/10) |
| 2 | Information regarding migraine shared on social media platforms is often inaccurate or misleading in Türkiye. | 0% | 70% | 30% | 0% | 0% |
|  |  | (0/10) | (7/10) | (3/10) | (0/10) | (0/10) |
| 3 | Social media influences patients’ expectations regarding treatment outcomes in Türkiye. | 30% | 70% | 0% | 0% | 0% |
|  |  | (3/10) | (7/10) | (0/10) | (0/10) | (0/10) |
| 4 | A considerable proportion of migraine-related content on social media originates from individuals without formal healthcare training or professional authorization in Türkiye. | 40% | 50% | 10% | 0% | 0% |
|  |  | (4/10) | (5/10) | (1/10) | (0/10) | (0/10) |
| 5 | Misinformation on social media regarding migraine complicates physician–patient communication and poses a barrier to appropriate patient care in Türkiye. | 30% | 60% | 10% | 0% | 0% |
|  |  | (3/10) | (6/10) | (1/10) | (0/10) | (0/10) |
| 6 | Healthcare professionals should play a more active role in disseminating accurate migraine-related information via social media in Türkiye. | 70% | 20% | 10% | 0% | 0% |
|  |  | (7/10) | (2/10) | (1/10) | (0/10) | (0/10) |
| 7 | Social media could be an effective tool for increasing awareness about migraine as a disabling neurological disease among the general population in Türkiye. | 70% | 30% | 0% | 0% | 0% |
|  |  | (7/10) | (3/10) | (0/10) | (0/10) | (0/10) |
| 8 | There is a need for more structured and evidence-based digital education platforms for migraine patients driven by headache experts in Türkiye. | 60% | 40% | 0% | 0% | 0% |
|  |  | (6/10) | (4/10) | (0/10) | (0/10) | (0/10) |
| 9 | Dissatisfaction with conventional migraine treatments frequently drives patients to seek alternative therapies (e.g., acupuncture, homeopathy, dietary supplements). | 50% | 50% | 0% | 0% | 0% |
|  |  | (5/10) | (5/10) | (0/10) | (0/10) | (0/10) |
| 10 | Active engagement of healthcare professionals and patient organizations on social media may be an effective strategy to reduce stigma surrounding migraine. | 60% | 40% | 0% | 0% | 0% |
|  |  | (6/10) | (4/10) | (0/10) | (0/10) | (0/10) |
| 11 | Social media engagement may help address socioeconomic barriers that limit access to effective migraine treatments in Türkiye. | 10% | 90% | 0% | 0% | 0% |
|  |  | (1/10) | (9/10) | (0/10) | (0/10) | (0/10) |
| 12 | In my clinical practice, patients’ reliance on online platforms for migraine information and self-management significantly affects clinical decision-making. | 10% | 50% | 20% | 20% | 0% |
|  |  | (1/10) | (5/10) | (2/10) | (2/10) | (0/10) |

# 2 Multiple-choice questions and aggregated responses

**1. In your experience, approximately what proportion of your migraine patients are influenced by social media content in terms of their treatment expectations, adherence, or symptom management?**

| **Response option** | **Aggregated response** |
| --- | --- |
| 0–10% | 10% (1/10) |
| 11–25% | 20% (2/10) |
| 26–50% | 40% (4/10) |
| 51–75% | 30% (3/10) |
| 76–100% | 0% (0/10) |

**2. Which aspects of your patients’ migraine experience are most influenced by social media content?**

*Respondents were asked to select the top three options that apply; therefore, percentages may exceed 100%.*

| **Response option** | **Aggregated response** |
| --- | --- |
| Choice of acute treatment | 0% (0/10) |
| Choice of preventive treatment | 30% (3/10) |
| Expectation of treatment efficacy | 60% (6/10) |
| Use of non-pharmacological or alternative therapies | 100% (10/10) |
| Awareness of newer treatment options | 50% (5/10) |
| Perceptions of medication side effects | 10% (1/10) |
| Perceptions of migraine severity/disability | 10% (1/10) |
| Trust in healthcare providers | 20% (2/10) |
| Other (please specify) | 0% (0/10) |

**3. In your clinical opinion, what is the most important potential benefit of social media in migraine care?**

*Respondents could select up to two options; therefore, percentages may exceed 100%.*

| **Response option** | **Aggregated response** |
| --- | --- |
| Increasing public awareness about migraine as a serious condition | 90% (9/10) |
| Reducing stigma and misconceptions | 100% (10/10) |
| Facilitating patient–physician communication | 70% (7/10) |
| Helping patients access reliable treatment information | 80% (8/10) |
| Providing peer support and community for patients | 90% (9/10) |
| Other (please specify) | 0% (0/10) |

**4. In your clinical experience, which of the following risks related to social media use have the greatest impact on migraine patients?**

*Multiple selections were permitted; therefore, percentages may exceed 100%.*

| **Response option** | **Aggregated response** |
| --- | --- |
| Spread of misinformation regarding migraine treatments | 100% (10/10) |
| Increased use of non-evidence-based or alternative therapies | 100% (10/10) |
| Delays in seeking professional care | 100% (10/10) |
| Heightened patient anxiety and misperceptions among migraine patients | 80% (8/10) |
| Other (please specify) | 0% (0/10) |

# 3 Open-ended responses

Open-ended responses are presented in anonymized form. Minor typographic edits were applied for readability without changing meaning.

**Response 1.** Social media could be an effective tool for increasing awareness about migraine as a disabling neurological disease among the general population in Türkiye.

**Response 2.** I believe that physicians without specific experience in migraine management, particularly non-neurology specialists such as neurosurgeons, often mislead the public on this topic. The suggestion that migraine can be cured through diet modifications or vitamin supplements is especially harmful to patient management, as such messages carry high credibility simply because they come from doctors.

**Response 3.** In my opinion, this is not a problem that can be solved easily or quickly. Misinformation about migraine constantly circulates on social media, and there is a group of people who believe and spread these false claims. Unfortunately, some non-medical individuals—and even certain healthcare professionals who are not neurologists—promote unproven “alternative” methods, often supported by organizations seeking financial gain rather than patient well-being. To address this issue effectively, education and awareness for both patients and the general public are essential first steps, because in my view, the more educated a society is, the greater progress it can make in understanding health issues and resisting misinformation.
